# Is radicalization a family issue? A systematic review of family‐related risk and protective factors, consequences, and interventions against radicalization

**DOI:** 10.1002/cl2.1266

**Published:** 2022-07-20

**Authors:** Izabela Zych, Elena Nasaescu

**Affiliations:** ^1^ Department of Psychology University of Cordoba Cordoba Spain

## Abstract

**Background:**

Family‐related risk and protective factors are crucial for different antisocial behaviors, but their role in radicalization requires synthesis. Radicalization is likely to have a negative impact on families, and well‐designed and implemented family‐focused intervention programs have the potential to decrease radicalization.

**Objectives:**

Research questions were: (1) What are the family‐related risk and protective factors for radicalization? (2) What is the impact of radicalization on families? (3) Are family‐based interventions against radicalization effective?

**Search Methods:**

Searches included 25 databases and hand searches of gray literature from April to July 2021. Leading researchers in the field were asked to provide published and unpublished studies on the topic. Reference lists of the included studies and previously published systematic reviews on risk and protective factors for radicalization were scanned.

**Selection Criteria:**

Published and unpublished quantitative studies on family‐related risk and protective factors for radicalization, the impact of radicalization on families, and family‐focused interventions were eligible with no restrictions regarding the study year, location, or any demographic characteristic. Studies were included if they measured the relation between a family‐related factor and radicalization or if they included a family‐focused intervention against radicalization. For family‐related risk and protective factors, radicalized individuals needed to be compared to general population. Studies were included if they defined radicalization as support or commission of violence to defend a cause, including support for radical groups.

**Data Collection and Analysis:**

The systematic search identified 86,591 studies. After screening, 33 studies focused on family‐related risk and protective factors were included, with 89 primary effect sizes and 48 variables grouped in 14 factors. For the factors that included two or more studies, meta‐analyses with random effects were conducted. When possible, moderator analyses were performed together with sensitivity and publication bias analyses. No studies on the impact of radicalization on families or family‐focused interventions were included.

**Results:**

The current systematic review based on studies with 148,081 adults and adolescents from diverse geographic locations showed that parental ethnic socialization (*z* = 0.27), having extremist family members (*z* = 0.26), and family conflict (*z* = 0.11) were related to more radicalization, whereas high family socioeconomic status (*z* = −0.03), bigger family size (*z* = −0.05), and high family commitment (*z* = −0.06) were related to less radicalization. Separate analyses described family‐factors for behavioral versus cognitive radicalization, and different radical ideologies including Islamist, right‐wing and left‐wing. It was not possible to distinguish risk and protective factors from correlates and the level of overall bias was mostly high. No results regarding the impact of radicalization on families or family‐focused interventions were included.

**Authors' Conclusions:**

Although causal relations between family‐related risk and protective factors could not be established, it is reasonable to suggest that policies and practice should aim at decreasing family‐related risks and increasing protective factors for radicalization. Tailored interventions including these factors should be urgently designed, implemented and evaluated. Studies focused on the impact of radicalization on families and family‐focused interventions are urgently needed together with longitudinal studies on family‐related risk and protective factors.

## 1 PLAIN LANGUAGE SUMMARY

### 1.1 Family factors are important for radicalization, but only limited evidence exists

This systematic review focuses on family‐related risk and protective factors for radicalization, the impact of radicalization on families, and family‐based interventions against radicalization. The review finds that parental ethnic socialization, having extremist family members and family conflict increase the risk of radicalization, whereas high family socio‐economic status, bigger family size, and high family commitment are protective factors.

### 1.2 What is this review about?

Radicalization to violence is extremely harmful to social groups and the society as a whole, and has been found to be related to terrorism. Terrorism is a significant threat in 21st century societies, and countering radicalization to violence has become a national and international policy priority and a crucial public safety issue worldwide.

There is reason to believe that families can be crucial to radicalization. Group influence on individual action is a well‐known phenomenon, and families are the most important social groups for many individuals. Transmission of antisocial behavior from parents to children has been confirmed in several studies, mostly explained by the fact that children learn by observing and imitating their parents. Parenting styles are also known to have short and long‐term impact on children's lives.

Thus, family‐related factors could be crucial to explain radicalization, but most of the empirical studies in the field include a limited number of participants and variables.

Families are also likely to be negatively impacted by radicalization and, given the importance of families for individuals and societies, family‐focused prevention and intervention programs against radicalization could be especially effective.

This Campbell Collaboration systematic review focuses on family‐related risk and protective factors for radicalization, impact of radicalization on families, and family‐based interventions against radicalization. The review examines evidence based on 89 effects from 33 studies.

### 1.3 What studies were included?

The review includes 33 studies on family‐related risk and protective factors, but there are no included studies on the impact of radicalization for families or family‐based interventions.

The review includes 14 family‐factors for radicalization. This is a broad set of factors, although the number of studies per factor was limited.

### 1.4 What are the main findings of this review?

The number of high‐quality studies on radicalization is relatively low. Given that high‐quality interventions against radicalization are urgently needed, it is crucial to inform practitioners, policy makers and researchers about possible components to be included in those interventions.

If risk and protective factors against radicalization are discovered using rigorous scientific methods, interventions could focus on decreasing risks and increasing protective factors. It is also crucial to identify the impact of radicalization on families so that this could be mitigated.

We found that parental bias and mistrust towards other cultures, having extremist family members and family conflicts were related to more radicalization. High family socio‐economic status, bigger family size and family commitment were related to less radicalization.

The review also describes family‐related factors separately for cognitive and behavioral radicalization, and for different radical ideologies such as Islamist, right‐wing and left‐wing. The results of this systematic review confirm the importance of families for radicalization, although they should be interpreted with caution, taking into account a relatively low number of studies per analysis. More studies on family‐related risk and protective factors for radicalization are needed.

### 1.5 What do the findings of this review mean?

Some family‐related factors seem to be crucial for understanding and preventing radicalization, but evidence is still limited. Family factors are among the most important predictors of delinquency in general, and this also seems to be true for radicalization.

Research on the impact of radicalization is urgently needed, and it is crucial to design, implement and evaluate family‐focused interventions against radicalization. These interventions should be evaluated through robust scientific designs, especially randomized controlled trials.

### 1.6 How up‐to‐date is this review?

The review authors searched for studies up to July 2021.

## 2 BACKGROUND

### 2.1 The problem

Radicalization to violence is a complex socio‐psychological process through which people acquire a series of extreme beliefs, attitudes, and ideologies, justifying the use of violence to achieve their goals and promote these ideologies (Borum, 2012; Doosje et al., 2016). Radicalization to violence is harmful to social groups and the society as a whole, in particular because of its association with terrorism (Dugas & Kruglanski, 2014). Terrorism is one of the most important threats faced by the 21st century societies. Thus, countering radicalization to violence has become one of the most important national and international policy priorities and a crucial public safety issue worldwide.

Several studies (e.g., Neumann, 2013; Schmid, 2013) suggest that radical thinking and attitudes do not necessarily imply violent behavior. Purely cognitive radicalization is not problematic *per se*, and radical beliefs are a part of any healthy democratic society, only becoming a problem if they are expressed through violent actions (Neumann, 2013). Radical violent behaviors are usually displayed only by a small number of radicalized individuals. Based on the two‐pyramids model (McCauley & Moskalenko, 2017), radicalization of opinion should be distinguished from radicalization of behavior. Although radical beliefs are not necessary or sufficient for becoming a terrorist (Schuurman & Taylor, 2018), it is usually assumed that individuals who engage in terrorism and radical violence show radical thinking first. For example, the staircase theory states that the process leading to terrorism is similar to a narrowing staircase where radical ideas appear before terrorist acts that occur “at the top of a building” (Moghaddam, 2005; p. 161). Thus, radical thinking can escalate to radical violence employed to achieve ideological, political, religious, social, or economic goals. This becomes a security threat because radical violent behaviors are justified by some individuals and groups as a way to promote extremist attitudes and ideologies (Doosje et al., 2016). It is therefore important to reduce both radical thinking and radical behavior. In this systematic review, we use the term radicalization to refer to a cognitive or behavioral process resulting in either extremism, or terrorism, all involving support for or the use of violence to defend a cause, including support for radical groups and terrorism.

Some of the efforts to describe and understand radicalization focus on family as “potentially being risky, as well as potentially being a source of protection and rehabilitation” (Spalek, 2016; p. 46). The role of the family often differs considerably from case to case. While some families might provide protective factors by their resources, positive parenting or developing resilience towards radicalization (Radicalisation Awareness Network, 2017; Spalek 2016), other families might provide risk factors by their poor resources and relationships or a direct undesirable ideological influence (King et al., 2011; Speckhard & Akhmedova, 2005). Family might not only facilitate and support radical and violent extremism activities (King et al., 2011), but more importantly, it might have a key role in preventing young people from radicalization and recruitment to violent extremist groups (RAN, 2017). Thus, families play an important role in radicalization, but empirical findings on the topic are inconclusive and a comprehensive research synthesis could clarify the role of family factors in radicalization.

The purpose of this systematic review is to provide a comprehensive synthesis of existing empirical studies on family‐related risk and protective factors for radicalization, the impact of radicalization on families, and the effectiveness of family‐related interventions against radicalization to build evidence‐based knowledge and guide future research, policy and practice. A comprehensive synthesis of family‐based intervention programs will make it possible to discover what is already being done and what works best. Discovering and understanding family‐related risk and protective factors for radicalization, and consequences of radicalization for family will advance knowledge of the etiology and impact of radicalization which will contribute to the improvement of prevention and intervention programs. Thus, this systematic review has a three complementary objectives of reviewing both risk and protective factors, consequences, and interventions focused on families and radicalization. A research synthesis can provide a global panorama of the field that cannot be obtained through singular empirical studies given the limited number of participants and variables that can be included in each project.

### 2.2 Family‐focused risk and protective factors for radicalization to violence

This systematic review includes studies focused on family‐related risk and protective factors for radicalization. Strictly speaking, a risk or a protective factor refers to a variable that associates with and precedes an outcome that should be compared between the affected population and general population free of the outcome of interest (Kraemer et al., 1997). Nevertheless, we anticipated that many of the included studies would be cross‐sectional and therefore measure theoretically defined risk and protective factors.

In this systematic review, family‐related risk factors for radicalization were defined as variables related to childrearing, family structure, family violence, and similar family‐related variables, that increase the risk of radicalization of opinion and behavior (e.g., corporal punishment by a parent, bullying by siblings). To be considered a risk factor, the associations should be tested by comparing radicalized individuals to a non‐radicalized group or high/low family‐factor in association with high/low radicalization.

Protective factors refer to variables that relate to low probability of negative outcomes (Lösel & Farrington, 2012). Family‐related protective factors are defined as variables related to childrearing, family structure, family relationships, and similar family‐related variables, that decrease the risk of radicalization of opinion and behavior. Some specific examples could include parenting practices, parental warmth and involvement, marital status, among other family‐related variables. To be considered a protective factor, the associations should be tested by comparing radicalized individuals to a non‐radicalized group or high/low family‐factor in association with high/low radicalization.

Family factors can be crucial for radicalization based on several theories and research findings. Among them, Sageman (2004) found that social networks, including families, were important in explaining terrorist actions, attributing this fact to group influence on individual actions that is a well‐known phenomenon in social psychology. Moreover, parents guide behaviors of their children and explain the standards of behaviors considered appropriate (Bandura, 1991). A study by Zych et al. (2020) showed that parental induction of moral disengagement, where children are told that immoral actions can be justified, was related to violent behaviors in children. Thus, some parenting practices and expression of radical ideas by parents could induce their children to adopt radical attitudes and behaviors. On the other hand, other parenting practices, or expressions of ideas against radicalization could be protective.

Intergenerational transmission of antisocial behaviors was confirmed in several studies (Farrington et al., 2009). This is usually explained by social learning theories according to which children treat parents as models and imitate their behaviors. It can also be true for the relations with other family members. A qualitative study based on interviews with violent extremists showed that children raised in extremist families are at higher risk of becoming violent extremists themselves (Schils & Verhage, 2017). Moreover, some structural factors such as unemployment relate to radicalization (Siedler, 2006) as these issues can potentially make it harder for families to be informal social control handlers. Although reviewing a broad range of structural factors not directly related to family factors (e.g., neighborhood poverty, country unemployment rates) is beyond the scope of this systematic review, many of them could explain why families should be studied in relation to radicalization.

Family‐related risk and protective factors could also be related to radicalization in an indirect way. During child development, parents have a crucial role in promoting emotional health and well‐being, including a positive sense of self, skills to cope with stressful situations, regulate emotions, control fears, or accept frustrations. Also, parents' ability to encourage children's sense of belonging is crucial for their early development which could also decrease radicalization. Several studies indicated that, among other factors, low sense of belonging makes young people more vulnerable to engage in violent and non‐violent radicalization (Borum, 2004; Ventriglio & Bhugra, 2019). Moreover, research suggests that low parental support, supervision, inconsistent parenting, or contact with family members with radical views enhance young people's vulnerability to radicalization (Sikkens, Sieckelinck et al., 2017; Van Bergen et al., 2016). Parenting is therefore related to mental health, individual and social well‐being which can become risk or protective factors for radicalization.

Although family‐focused risk and protective factors were found among the strongest and most robust predictors of different antisocial behaviors including delinquency (Zych et al., 2021), there are still pressing gaps in knowledge related to family and radicalization. The existing studies suggest that there are different family‐related variables, including risk and protective factors, that relate to radicalization (Harris‐Hogan, 2014; Schmid, 2013). Among them Nivette et al. (2017) focused on the relation between parental involvement in adolescents' everyday life and violent extremist attitudes, Siedler (2006) examined whether parental unemployment during childhood and parental leaning towards right‐wing extremists were related to right‐wing extremism in their children, Jasko et al. (2017) studied if having a family member involved in radical activities was related to the commitment of ideologically motivated crimes, and Dhumad et al. (2020) focused on the relation between being convicted for terrorism and family factors such as an authoritarian father, disintegrated family or having a family member that had been murdered.

Sikkens, Van San et al. (2017) also explored the influence of family and the socialization context on radicalization. In a qualitative study, Sikkens, Sieckelinck et al. (2017) conducted a study aimed at examining parents' reaction when children developed radical ideology and concluded that parents often lack skills and strategies to cope with the situation. It is therefore possible that parental lack of reaction and response could facilitate children's radicalization. Thus, there are some empirical studies that identify family factors related to radicalization, but they provide inconsistent evidence regarding risk and protective factors. Most of the empirical studies provide evidence focused on specific risk and protective factors in specific contexts, and a research synthesis is needed to establish what is known and unknown.

### 2.3 Impact of violent radicalization on families

The family has a crucial role as a socialization context that can provide emotional support and influence social identities of its members. Nevertheless, family can also have an undesirable influence on its members (Zych et al., 2020). Radicalization of a family member could negatively impact other family members, but empirical studies focused on the impact of radicalization on families are inconclusive. In this systematic review, family‐related consequences are defined as variables related to the psychological, physical, and structural impact of radicalization on families (e.g., divorce, mental health issues of family members).

Radicalization might have damaging psychological and social effects on the family (Guru, 2012a, 2012b). Some studies suggest that families of radicalized individuals are victimized by others as they may become socially isolated (Gielen, 2015; Guru, 2012a, 2012b). Regarding consequences of radicalization for families, research shows that family members of radicalized individuals are frequently shamed, blamed and socially rejected which can be related to mental health issues (Guru, 2012a, 2012b). Labeling is a well‐known phenomenon in social sciences, according to which individuals start to behave according to labels given to them by others (Scheff, 1974). Labeling was found to be related to intergenerational transmission of crime (Besemer et al., 2017), and it is possible that family members of radicalized individuals are labeled. Labeling could be one of the mechanisms through which radicalization impact family members.

Family members of radicalized individuals can suffer internalizing problems such as anxiety and depression (Guru, 2012a, 2012b). This might cause a polyvictimization process that could increase the risk of radicalization of the previously non‐radicalized family members over the lifespan. Moreover, secondary victimization may occur when a victim suffers additional harm, being treated in an unfair way, including victim‐blaming attitudes (Williams, 1984). It is possible that family members of radicalized individuals suffer an indirect harm through secondary victimization.

It has been suggested that radicalized individuals focus on specific goals and sometimes “family and relationships are forgotten” (Kruglanski et al., 2014, p. 71). According to Sampson and Laub (1995), families are important resources to draw on during life transitions and turning points. Thus, if a family member is focused on radical goals, ignoring other aspects of life including the family, these important resources can be lost. Social capital has been defined by Coleman (1988) as social structures that facilitate certain actions within the structures, making it possible to achieve certain goals. Social capital is based on trust and there are certain norms within social structures. If a family member becomes radicalized, the whole structure is likely to be affected. Within the structures formed by radicalized family members, prosocial actions could be dissuaded, and antisocial actions could be promoted. Also, radicalization of other family members could become a goal. Thus, negative consequences of radicalization for family members are likely.

### 2.4 Family‐focused interventions for countering radicalization

#### 2.4.1 The intervention

There are some family‐based interventions that have been implemented to decrease radicalization. For example, an 18‐month‐long pilot project “Ending Terrorism Through Youth Service Action Locally” (ETTYSAL) in Tunisia funded by the U.S. State Department and implemented by Creative Associates International (n.d.) focused on the importance of the family as a protective factor against radicalization. In this program, 100 Tunisian young people were evaluated for vulnerability to join extremist groups based on 12 risk factors (antisocial tendencies, poor parental supervision, family radicalization, critical life events, impulsive risk‐taking, neutralization of guilt, deviant behaviors, peer influence, peer radicalization, religious extremism and social vulnerability). The intervention was individualized and focused on family counseling and group activities. ETTYSAL was evaluated to discover if this family‐centered intervention approach reduced the risk factors for radicalization to violent extremism after the program and check if family radicalization was an important risk factor that contributed to this decrease.

Effectiveness of family counseling was studied also in programs carried out in Europe. In Germany, *Hayat* (means “life” in Turkish) is a prominent program based on family counseling (Koehler, 2013). It aims at reducing violent and non‐violent radicalization at any stage. Despite some encouraging results, the effectiveness and impact of this intervention program against radicalization still needs to be quantitatively assessed.

According to the Radicalization Awareness Network, evaluation of the effectiveness and impact of the intervention programs is one of the pressing gaps in the literature regarding radicalization (Pisoiu & Ahmed, 2016). Thus, it is imperative to evaluate the existing family‐related intervention programs against radicalization through a rigorous methodology (Feddes & Gallucci, 2015). There is still much to be addressed and learned about how to better design, implement, and evaluate effective intervention programs against radicalization. A systematic review could help better understand the state‐of‐the‐art and identify which family‐related components of intervention programs showed evidence to be effective against radicalization.

In this review, family‐based interventions refer to any activity, strategy, technique, training, and program that involves family as a recipient of the intervention or to the interventions related, focused, or targeted on family‐related risk and protective factors to decrease radicalization. In this systematic review, we planned to synthesize knowledge regarding family‐based interventions to prevent radicalization, and also interventions focused on deradicalization. Interventions could focus on increasing protective factors and decreasing risk. Studies focused on interventions are different from studies on protective factors because they include an explicit manipulation of independent variables by the intervention providers.

#### 2.4.2 How the intervention might work

Family‐based interventions may be effective if they focus on family related risk and protective factors associated with radicalization. If family is one of the most influential groups for individuals, and groups influence individual's behaviors including terrorist acts (Sageman, 2004), family‐based interventions could promote desirable goals and deradicalization. Given that parents guide children's behaviors (Bandura, 1991), it is possible that family‐based interventions promote desirable parental influence. Interventions can also improve social capital based on families and provide resources against radicalization (Koehler, 2015). Moreover, interventions could improve family's capacity to promote self‐control, which is especially important because low self‐control is related to antisocial behavior, and its level is influenced by family (Gottfredson & Hirschi, 2020). According to social control theory (Hirschi, 1969), antisocial behavior is inhibited by strong and long‐term bonds with others, including parents who may or may not have undesirable opinions and beliefs. Thus, family could be key to understanding and preventing radicalization, but more research is needed to confirm this.

### 2.5 Why it is important to do this review

Despite a growing body of research on radicalization, studies focused on family‐factors are still in their early stages. A family‐specific focus, including parents, siblings, children, spouses, and extended family members could fill pressing gaps in knowledge that would make it possible to understand family impact on radicalization, including its cognitive and behavioral components. In our systematic review, family is defined as a group related by consanguinity, adoption, marriage, and similar long‐term couple relationships.

There are several research syntheses focused on radicalization, but none of them focus specifically on family‐related variables and interventions. Among them, a systematic review focused on protective factors against extremism and violent radicalization was published by Lösel et al. (2018). This systematic review was based on comprehensive searches in 15 databases and included different individual, family, school, peer, community, and society factors related to radicalization. Among family factors, variables such as parenting style, significant others who do not use violence, and owning a house were identified as protective factors. Although this systematic review provides valuable information on the topic, family‐related search terms were not included in literature searches. Moreover, family‐related risk factors, consequences, and interventions were not reviewed. Thus, the current review differs from Lösel et al. (2018) as it also includes risk factors, consequences, interventions, and specific search terms that could locate all the studies specifically focused on family and radicalization.

A Campbell Collaboration systematic review focused on putative risk and protective factors for cognitive and behavioral radicalization (Wolfowicz et al., 2020, 2021). Some results regarding putative factors for radicalization were also published by Wolfowicz et al. (2019). The authors found that having children, being married, parental education, parental involvement and control were protective factors against radical attitudes, whereas family violence and parental abuse were risk factors. Parental involvement was a protective factor against radical behavior, and having radical family members was a risk factor. Whilst the systematic review conducted by Wolfowicz et al. (2019, 2021) represents an important contribution providing a better understanding of radicalization, the current review will specifically focus on family, including specific searches focused on family‐related risk and protective factors, consequences of radicalization for families, and family‐related interventions against radicalization. The current review differs from Wolfowicz et al. (2019, 2021) as it also includes consequences, interventions, and specific family‐related search terms that will result in locating additional studies specifically focused on families.

A systematic review and meta‐analysis on risk factors for violent radicalization in juveniles was recently published by Emmelkamp et al. (2020). Based on 6 studies and 12 effect sizes, they found that the relation between negative parenting and radicalization was not statistically significant. Again, this systematic review provided valuable information on risk factors for radicalization, but this information differs from the current systematic review. Emmelkamp et al. (2020) analyzed negative parenting, but they did not analyze other family‐related risk factors or protective factors. Moreover, they only included juveniles, and the current systematic review focused on all age groups. Contrary to the current systematic review, specific family‐related search terms were not used.

Thus, there are no existing or registered systematic reviews specifically focused on family and radicalization. None of the previous reviews focused on both risk and protective factors, included family‐related consequences, and/or family‐related interventions. Our systematic review will address important gaps in the literature providing a global panorama regarding family factors and family‐related interventions and radicalization, including extensive literature searches specifically focused on family and radicalization, which will enable solid inferences about what is known and what may work to counter radicalization within a family‐focused framework.

There are increasing efforts to describe, understand and decrease radicalization globally. Addressing radicalization and eliminating terrorism are among the most important national and international policy priorities. Although different groups become important in adolescence and adulthood, including peers, coworkers, and other social networks, families are a crucial part of the social capital of individuals (Coleman, 1988; Hoffmann & Dufur, 2018). Thus, our systematic review provides insights and contribute to a bigger picture regarding radicalization, making it possible to improve evidence‐based policy and practice.

Having a global vision and comprehensive understanding of family‐related factors and interventions will make it possible to decrease risks and increase protective factors which will potentially reduce radicalization, together with its detrimental consequences. After analyzing which family‐based programs and program components are effective to decrease radicalization, a new generation of prevention policy and practice can be designed, including components focused on the most important risk and protective factors, and consequences. Thus, policymakers will obtain valuable information that can be crucial for the design and development of a new generation policy and practice against radicalization which is not available yet. Therefore, this systematic review provides important information for evidence‐based policy and practice.

## 3 OBJECTIVES

This systematic review aimed to answer the following research questions:

1. What are the family‐related risk and protective factors for radicalization?
2. What is the impact of radicalization on families?
3. To what extent are family‐based interventions against radicalization effective?

The review aimed to answer these research questions by systematically gathering and synthesizing published and unpublished scientific literature on family‐related risk and protective factors for radicalization, the impact of radicalization on family, and studies that evaluate the impact of family‐based interventions on radicalization. Evidence permitting, this review also aimed at exploring what components of family‐based interventions are most effective for countering radicalization. Thus, this systematic review provides a global vision of scientific literature focused on family and radicalization.

## 4 METHODS

Details on methodology for this systematic review were published in a previously registered protocol (see Zych & Nasaescu, 2021). Only methods actually used in the current systematic review are described below. Methods that were planned but were not used due to study limitations are omitted and can be found in the review protocol. These unused methods refer to the treatment of studies that could have been within the scope of this systematic review but were not located or included. For example, methods for synthesizing results of family‐related interventions are not detailed in this systematic review because no family‐related interventions were identified.

### 4.1 Criteria for considering studies for this review

#### 4.1.1 Types of studies

This systematic review sought to include quantitative studies focused on family‐related risk factors, protective factors, consequences and interventions against radicalization. Empirical studies were included, and theoretical studies or editorial materials were excluded. Nevertheless, systematic reviews on risk and protective factors for radicalization were used for reference screening. Both published and unpublished studies that met the inclusion criteria were included.

##### 4.1.1.1 Objective 1: Risk and protective factors

Studies were included if they provided empirical data on the relation between any possible family‐related risk factor, protective factor, and radicalization. Family‐related factors and radicalization (including attitudes and behaviors) needed to be explicitly measured. These factors needed to focus on family structure, characteristics, attitudes and behaviors of family members, or interpersonal relations within families. These could be correlational studies, or studies that compared groups with and without the risk or protective factor. Study designs focused on family‐related risk and protective factors were expected to be cross‐sectional and preferably longitudinal. Although cross‐sectional designs are weaker than longitudinal designs, it is common to study risk factors for different problem behaviors through cross‐sectional studies on a theoretical basis. A moderator analysis was expected to be run to assess the impact of cross‐sectional versus longitudinal designs, but the number of longitudinal studies included in this systematic review was too low. In cross‐sectional studies, risk and protective factors were defined on a theoretical basis and treated as independent variables, whereas radicalization was treated as a dependent variable. In longitudinal studies, risk and protective factors preceded radicalization.

##### 4.1.1.2 Objective 2: Family impact of radicalization

Studies were expected to be included if they provided empirical data on the impact of radicalization on family and family environment. Family‐related consequences of radicalization needed to be explicitly measured. Correlational studies were included, along with studies that compared groups including radicalized versus non‐radicalized individuals in relation to family‐related consequences. Study designs focused on family consequences of radicalization were expected to be cross‐sectional and preferably longitudinal. In cross‐sectional studies, family‐related consequences were expected to be defined on a theoretical basis and treated as dependent variables, whereas radicalization was expected to be treated as an independent variable. In longitudinal studies, radicalization was expected to precede the family impact.

##### 4.1.1.3 Objective 3: Family‐focused interventions

Intervention programs were expected to be included if they were randomized controlled trials, where participants are randomly assigned to experimental (intervention) or control conditions (without an intervention), or quasi‐experiments with robust designs (non‐randomized experimental vs. control group including a pre‐test and a posttest measures, and matched designs). Given the emerging nature of the extant literature, one group pretest posttest intervention studies were expected to be included for a descriptive purpose only, but not included in any meta‐analyses.

#### 4.1.2 Types of participants

##### 4.1.2.1 Objective 1: Risk and protective factors

The systematic review sought to include international research conducted with any type of population if family‐related risk or protective factors, and radicalization in at least one family member were measured. Family referred to members by consanguinity (i.e., mother, father, children, siblings, cousins, aunts, uncles, and grandparents), and family members by marriage (i.e., husbands, wives, and long‐term partners). There were no restrictions regarding study location or any characteristic of the participants. Thus, the review included participants of any age, gender, ethnicity, socioeconomic status, and family structure. Populations from any part of the world, including low‐middle‐ and high‐income countries were expected to be included. Only studies that compared radicalized individuals with non‐radicalized general population or high/low family‐factor in association with high/low radicalization (i.e., correlational studies) were included. Studies that compared violent and non‐violent radicals (e.g., LaFree et al., 2018), recidivist terrorists with non‐recidivists (e.g., Altier et al., 2021), or extremists with gang members (e.g., Pyrooz et al., 2018) were excluded.

##### 4.1.2.2 Objective 2: Family impact of radicalization

This systematic review was not restricted to any specific study location or any characteristic of the participants regarding family impact of radicalization if radicalization was measured in at least one family member together with its impact on at least one family member. Thus, this systematic review sought to include participants of any age, gender, ethnicity, socioeconomic status, and family structure. Also, populations from any part of the world, including low‐middle‐ and high‐income countries were expected to be included. Again, family referred to members by consanguinity and marriage. Radicalized individuals were expected to be compared to general population.

##### 4.1.2.3 Objective 3: Family‐focused interventions

For family‐focused interventions, this systematic review intended to include any type of population with at least one radicalized family member (by consanguinity and marriage) or at least one family member labeled as at‐risk of radicalization. Again, no restrictions regarding study location or characteristics of the participants were used. Participants of any age, gender, ethnicity, socioeconomic status, and family structure, from any part of the world, were expected to be included.

#### 4.1.3 Types of risk and protective factors

A family‐related risk factor was defined as any factor related to families that increased the risk of radicalization in a family member. A family‐related protective factor was defined as any factor related to families that decreased the risk of radicalization in a family member. In longitudinal studies, these factors preceded radicalization. In cross‐sectional studies, these factors were conceptualized as risk and protective factors, and they were treated as independent variables. Individuals or groups could be exposed to these factors at any moment of their lives. Family‐related factors were variables that described relationship styles, bonding, characteristics of families or family‐members, and circumstances within families. These could include, but were not limited to, parenting styles, marital status, divorce, parental behavioral problems, involvement, and unemployment. Studies or variables that focused on family and other social groups (e.g., questions about family or friends being involved in violence, Cragin et al., 2015) were excluded because participants could be answering thinking about groups other than the family.

#### 4.1.4 Types of variables associated with family‐related consequences

A family‐related consequence was defined as any variable that supposed an undesirable consequence of radicalization for family members. In longitudinal studies, radicalization needed to be measured first, and consequences needed to be measured afterwards. In cross‐sectional studies, consequences were expected to be conceptualized on a theoretical basis and treated as dependent variables.

#### 4.1.5 Types of interventions

This systematic review pretended to include any intervention that aimed to modify family‐related factors to decrease cognitive or behavioral radicalization. Specifically, interventions to be included were expected to:

1. Include family‐related risk or protective factors among the intervention components, or
2. Include families as the recipients of the interventions, or
3. Include specific components to prevent or buffer family‐related consequences.

These interventions were expected to include, for example, family counseling and individual or group interventions focused on family‐related risk and protective factors for radicalization. Any intervention modality could have been included, such as individual or group, face‐to‐face and online, manualized and unstructured interventions. Different types of programs including therapeutic, educational, and other types were expected to be included. Programs could have been implemented by researchers, educators, independent program developers and other providers. Interventions were expected to be focused on both family members and family‐related risk and protective factors. Interventions with a family component combined with other components not relevant to this systematic review were expected to be included in the systematic review, but they were planned to be excluded from the meta‐analysis if they did not provide a specific evaluation of the family component. Moderator analyses were planned to be performed to check if these different characteristics of the interventions influenced the results.

#### 4.1.6 Types of outcome measures

##### 4.1.6.1 Objectives 1 and 3: Risk and protective factors and family‐focused interventions

This systematic review included a broad range of outcomes related to radicalization, including radicalization to violence, extremism and terrorism including their different types such as right‐wing, left‐wing, religious extremism, and any other type of radicalization or extremism measured in the primary studies. Although these terms are frequently used interchangeably, there are certain differences regarding their definitions:

1. Extremism is defined as ideas that are opposed to mainstream fundamental social values such as democracy, liberty and respect (HM Government, 2015).
2. Violent extremism is defined as beliefs and actions of individuals who engage in violent acts or support the use of violence to achieve goals related to their extreme ideas (Canada Centre for Community Engagement and Prevention of Violence, 2018).
3. Radicalization is a process through which individuals support and engage in activities that violate social norms shared by other members of the society (Kruglanski et al., 2014). Radicalization has also been defined as a process of adopting extreme views that differ from the mainstream beliefs and have a strong ideological basis (Bartlett & Miller, 2012). Although these beliefs *per se* are not necessarily a threat, according to the European Commission (2005a), they could lead to terrorism.
4. Radicalization to violence is defined as a process through which people acquire a series of beliefs, attitudes and ideologies, justifying the use of violence to achieve social goals and promote their ideas (Doosje et al., 2016). Radicalization to violence can include a cognitive component defined as attitudes and ideas that support violence as a means to promote these radical ideas, also including intentions to perpetrate these acts of violence. It also includes a behavioral component which consists of committing acts of extremist violence to promote radical ideas.
5. Terrorism has been defined as a commission of a terrorist act or joining a group to contribute to terrorist offences (European Commission, 2005b).

Based on the two‐pyramids model (McCauley & Moskalenko, 2017), outcomes were measured taking into account both radicalization of opinion and radicalization of behavior. Radical and extremist attitudes were measured mostly with self‐reported measures such as questionnaires. Radical behaviors were measured with self‐reports, but they were also measured with official records of radical behaviors including violent extremism and terrorist acts.

Included studies could focus on any of these outcomes as long as the outcome was defined as support or commission of violence to defend a cause, including support for radical groups (e.g., neo‐Nazi) and terrorism. Studies that did not meet this definition were excluded. For example, Rico and Jennings (2016) focused on self‐identifying as Spanish or Catalan, Saini and Vasudeva (1977) defined radicalization as conservatism regarding traditional education and women´s role in the society, Moss and Peter (2020) focused on political correctness and white identarian attitudes, Baier et al. (2010) mixed right‐wing extremism with xenophobia and classified participants as radicals if they responded yes to either of these two, and several other studies focused on voting for radical parties. None of these were included in the current review.

##### 4.1.6.2 Objective 2: Family impact of radicalization

Consequences of radicalization for families were also planned to be studied. These outcomes were expected to focus on any negative impact of radicalization on families that could include, but were not be limited to, social exclusion, broken families, negative impact on wellbeing, mental health issues, etc. These outcomes were expected to be measured taking into account both self‐reported and other‐reported measures regarding family‐related impact of radicalization. In longitudinal studies, radicalization was expected to be measured first and these negative consequences were expected to be measured afterwards. In cross‐sectional studies, consequences were expected to be defined on a theoretical basis and treated as dependent variables. Family impact of radicalization was expected to be measured mostly with self‐reports and other‐reports measures such as questionnaires.

#### 4.1.7 Duration of follow‐up in family‐focused interventions

Studies reporting any duration of follow‐ups were eligible for inclusion. Studies were hoped to be grouped according to the duration of the follow‐up periods in categories such as: studies with a short follow‐up (0–3 months), studies with a medium follow‐up (between 3 and 6 months), and studies with a long‐term follow‐up (more than 6 months).

#### 4.1.8 Types of settings

There were no search limitations regarding the year, language, or geographical area. Thus, studies that met the inclusion criteria were included regardless of their settings. Searches were conducted in English, but studies in any language were included if located. Google Translate was used for languages not understood by the authors of this systematic review (other than English, Spanish, French, Italian, Polish, Portuguese, and Romanian), and colleagues were asked for help if languages were not well translated by Google Translate (e.g., from Arabic, Aotaibi, 2020).

### 4.2 Search methods for identification of studies

Systematic literature searches were performed from April to July 2021 on an extensive range of search locations to ensure that published as well as unpublished studies were located.

#### 4.2.1 Electronic searches

For the identification of eligible studies, we searched titles, abstracts, keywords and/or subject/indexing terms with a combination of search terms using the Boolean operators “AND” and “OR.” These terms were combined with the following terms searched in titles, abstracts, keywords and/or subject/indexing terms:

radicali* OR terror* OR extremis* OR “lone wol*” OR lone‐wol* OR “foreign fighter*” OR “single issue” OR Jihad* OR Islamis* OR Salaf* OR left‐wing OR far‐left OR right‐wing OR far‐right OR neo‐nazi* OR communis* OR nationalis* OR supremacist OR anarch* OR indoctrinat*

AND

family OR families OR familial OR parent* OR siblin* OR brother* OR sister* OR father* OR mother* OR child* OR son* OR daughter* OR cousin* OR uncle* OR aunt* OR generation* OR maternal OR paternal OR grandparent*

AND

risk* OR protect* OR factor* OR correlat* OR relat* OR predict* OR caus* OR determina* OR consequenc* OR interven* OR evaluat* OR program* OR treat* OR prevent* OR experiment* OR “cross‐section*” OR longitudinal* OR regress*

Electronic searches for the identification of studies were performed in different academic databases including *Campbell Systematic Reviews* and Cochrane Database of Systematic Reviews for reviews to scan reference lists, Criminal Justice Abstracts (EBSCO*host*), Google Scholar (searches in titles only combining radicalization related terms with family related terms), ProQuest Platform (including, APA PsycArticles®‎, APA PsycInfo®‎, Health & Medical Collection‎, MEDLINE®‎‎, Periodicals Archive Online‎, Periodicals Index Online‎, ProQuest Dissertations & Theses Global, Psychology Database‎, and Publicly Available Content Database‎), Sage Journals Online and Archive, ScienceDirect, SCOPUS, Taylor & Francis Online, Web of Science (including Core Collection, Current Contents Connects, Derwent Innovations Index, Korean Journal Database, Russian Science Citation Index, SciELO Citation Index), and Wiley Online Library. For each search location, information such as search location, date of the search and exact search syntax used were recorded (see Supporting Information: Appendix 1).

##### 4.2.1.1 Searching other resources

We also searched for gray literature on the websites of different agencies and professional organizations which include studies focused on countering radicalization (including search engines and publications sections):

- Department of Homeland Security (https://www.dhs.gov/topic/preventing-terrorism)
- Global Centre on Cooperative Security (https://www.globalcenter.org/publications/)
- Global Terrorism Research Centre (http://artsonline.monash.edu.au/gtrec/publications/)
- Hedayah (https://www.hedayahcenter.org/programs/)
- Impact Europe (http://impacteurope.eu/)
- National Consortium for the Study of Terrorism and Responses to Terrorism (START, https://www.start.umd.edu/radicalization-and-deradicalization)
- National Criminal Justice Reference Service (https://www.ojp.gov/ncjrs/new-ojp-resources)
- Public Safety Canada (https://www.publicsafety.gc.ca/index-en.aspx)
- Radicalisation Awareness Network (RAN, https://ec.europa.eu/home-affairs/what-we-do/networks/radicalisation_awareness_network_en)
- Radicalisation Research (https://www.radicalisationresearch.org/)
- Royal United Services Institute (RUSI, https://rusi.org/)
- Terrorism Research Centre (http://www.terrorism.org/)

To identify more eligible studies, references of the included studies and references of the previously published narrative and systematic reviews were screened. Documents citing the included studies were also screened in Google Scholar, Scopus, and Web of Science. Moreover, hand‐searches of 2020 and 2021 volumes of the following journals were performed:

- *Behavioral Sciences of Terrorism and Political Aggression*
- *Critical Studies on Terrorism*
- *Dynamics of Asymmetric Conflict*
- *Journal of Policing, Intelligence and Counter Terrorism*
- *International Journal of Conflict and Violence*
- *Journal for Deradicalization*
- *Journal of Interpersonal Violence*
- *Perspectives on Terrorism*
- *Studies in Conflict & Terrorism*
- *Terrorism & Political Violence*

Authors of the located studies and other experts in the field were contacted and asked to provide published and unpublished studies focused on family and radicalization.

### 4.3 Data collection and analysis

#### 4.3.1 Criteria for determination of independent findings

This systematic review included each independent study in each analysis only once. All relevant effect sizes were coded, and the dependencies issue were solved at the analysis stage by selecting independent subsets for each analysis. When there were multiple effect sizes relevant to a particular analysis, they were combined to handle multiple effect sizes per study, including combining groups and combining outcomes (using CMA, based on Borenstein et al., 2006). If there were effect sizes available for the whole group and subgroups (e.g., males and females), only the effect size for the whole group was used. If effect sizes were only available for subgroups, they were combined treating data as uncorrelated according to Borenstein et al. (2006).

At the analysis stage, effect size multiplicity was dealt with by identifying and categorizing the types of multiplicities in the included studies. Then, a reductionist approach (i.e., only one effect size extracted from a primary study) was used when only one effect size was relevant according to the objectives of this systematic review and an integrative approach where effect sizes were combined was used when there was more than one conceptually similar effect size per study (López‐López, Page, Lipsey and Higgins, 2018).

Studies in the field can sometimes report multiple and similar variables using the same sample (e.g., discussed politics with mother in relation to radicalization and discussed politics with father in relation to radicalization, European Values Study, 2008), data from the same project can be published in multiple documents (e.g., a doctoral thesis and a journal article, Clemmow, 2020; Clemmow et al., 2020), and data from one project can be reported taking into account different subsamples in the same paper or in different papers (e.g., males and females, Jahnke et al., 2021).

When multiple outcomes or predictors were reported in the same paper, their identification was straightforward. These were reported as one study in the systematic review and combined in the meta‐analyses to one effect per project. Studies published by the same authors and studies with some overlap in research teams (or specific projects) were analyzed as possibly reporting findings that were not independent. When these findings were reported in different documents, methodology sections were thoroughly analyzed to check if samples were the same. When two or more documents reported the results of the same study and there were documents that did not provide any additional information, these were excluded. This was the case, for example of Kim (2017) that was based on a publicly available data set and its documentation that was analyzed by the authors of this systematic review (McCauley, 2011). If two or more reports were complementary (i.e., from the same study, but providing results on different outcomes or family‐factors), they were described as one study in the systematic review and they were included only once in each meta‐analysis. Multiple outcomes (e.g., cognitive and behavioral radicalization) were also included in moderator analyses.

When two or more effects per study were based on the same participants, they were treated as combined outcomes. Given that correlations among the combined effects were usually not known, and Comprehensive Meta‐Analysis Software (Borenstein et al., 2006) was used for the analyses, a conservative approach was taken assuming a maximum possible correlation (*r* = 1). This is the only option given by the software when combining outcomes, unless outcomes are treated as independent. A sensitivity analysis was run treating all the outcomes assuming independence to check if results would change with a less conservative approach.

#### 4.3.2 Selection of studies

After comprehensive searches in electronic databases, studies located in databases that allowed exporting references to reference management tools were imported into EndNote software (Criminal Justice Abstracts, ProQuest Platform, Sage Journals, Science Direct, SCOPUS, Taylor & Francis, and Web of Science). References located in databases that did not allow direct exporting were screened afterwards in each database (Campbell Systematic Reviews and Cochrane Database of Systematic Reviews for reviews to scan reference lists, Google Scholar, and Wiley). Records that were not already included in the full texts database and were eligible based on titles and abstracts were added. Additional searches were performed on the websites of different agencies together with hand‐searches of specialized journals.

Screening was performed in Excel spreadsheets. For all the located studies, titles were screened first and, if a study was potentially eligible based on title, abstracts were screened. Studies were considered potentially eligible based on titles and abstracts if they included terms related to radicalization and terms related to family, and did not explicitly state that they were qualitative. Finally, if a study was potentially eligible based on both title and abstract, full texts were screened according to the inclusion and exclusion criteria. All these stages were performed by two independent researchers. The agreement rate at the stage of title and abstract screening was 99.79%. Any possible doubts and discrepancies were solved through a careful analysis, discussion, and consensus.

Additionally, authors of the located studies and other experts in the field were contacted and asked to provide published and unpublished studies on family and radicalization. When received, authors of this systematic review checked if they were already among the located studies. When new records were sent by the contacted experts, they were screened for eligibility based on titles and abstracts. If eligible, they were incorporated to the database and their full texts were screened together with other located studies.

Any quantitative study that focused on family and radicalization based on titles and abstracts was considered potentially eligible and saved for a full text screening that was also performed by the two authors of this review. Full texts were screened according to the inclusion and exclusion criteria using an eligibility screening form (see Table 1). The agreement rate at the full text screening stage was 93.7%. Disagreements were solved by going back to the empirical studies, double checking their content, discussion and consensus. A PRISMA flowchart was compiled to depict the flow of studies through the selection process.

**Table 1:** Eligibility screening form

Eligibility criteria	Decision
Empirical quantitative data included	Yes
	No
	Cannot tell
	If no, stop here
Relation between a family factor and radicalization is measured and reported or there is an intervention	Yes
	No
	Cannot tell
	If no, stop here
There is a comparison group from general population (e.g., non‐radicalized, varying degrees of radicalization) or an eligible intervention	Yes
	No
	Cannot tell
	If no, stop here
Definition of radicalization is met (support or commission of violence to defend a cause, including support for radical groups or terrorism)	Yes
	No
	Cannot tell
	If no, stop here
Definition of a family factor is met (any factor related to family including relationship styles, bonding, characteristics of families or family‐members, and circumstances within families)	Yes
	No
	Cannot tell
	If no, stop here
Data set is not duplicated with another more comprehensive study without providing new information	Yes
	No
	Cannot tell
	If no, stop here

#### 4.3.3 Data extraction and management

A coding sheet including all the relevant information from each study was developed and applied (see Zych & Nasaescu, 2021). As delineated in the review protocol, there were three similar coding sheets for the three parts of this systematic review, but only the one focused on family‐focused risk and protective factors was used because no studies on family‐related consequences and interventions were included. Studies on family‐focused risk and protective factors were coded including: study, location and document type, participants, methodology, family‐related risk and protective factors (terms and instruments), radicalization‐related outcomes (terms and instruments), results (unadjusted), and results (adjusted).

Each study was coded by the two authors of this systematic review. Disagreements were solved through discussion and consensus. An agreement rate between the two coders was 98.08%.

### 4.4 Assessment of risk of bias in included studies

This review assessed the risk of bias taking into account the quality of the included studies. Although we did not exclude studies from the review based on quality, each project was assessed, and the quality of each paper explicitly reported. Moderator analyses based on the quality of the included studies were performed. This section pertains only to risk of bias for review Objective 1 (risk and protective factors), as no studies were identified for Objectives 2 and 3.

Quality of the studies was assessed through the Cambridge Quality Checklist designed by Murray et al. (2009). The checklist was applied by both authors independently, differences were discussed and resolved by consensus. An agreement rate for quality assessment was 97.64%. Table 2 shows the details regarding the application of the quality criteria to the included studies.

**Table 2:** Quality assessment according to the Cambridge Quality Checklist

Criterion	Scoring
Sampling	Studies including the whole population or a random sample were rated as high quality and studies with convenience sampling or case‐control design were rated as low quality.
Response rates	Response rates above 70% and attrition below 10% were rated as high quality, response rates below 70% and attrition above 10% were rated as low quality.
Sample size	Studies with a sample size above 400 were rated as high quality, studies with a sample size below 400 were rated as low quality.
Measure of radicalization	Measures with all reliability coefficients above 0.75 and face validity or convergent validity above 0.30, more than one instrument to measure each construct or official records were rated as high quality, other measures as low quality.
Measure of family factors	Measures with all reliability coefficients above 0.75 and face validity or convergent validity above 0.30 or more than one instrument to measure each construct, or demographic variables only were rated as high quality, other measures as low quality.
Methodology used for studying risk/protective factor	Cross‐sectional designs were scored as low, retrospective designs were scored as medium and longitudinal prospective designs (or only with fixed family‐factors) were scored as high quality.
Causal risk/protective factors	Studies with no comparison group and analysis of change were scored as very low, studies with a comparison group but no adequate control of confounders or change were scored as low, studies with no comparison group but a measure of change were scored as medium low, studies with a comparison group and a measure of change but no control of confounders were scored as medium high, studies with a comparison group statistically balanced or matched on confounders were scored as high, controlled nonexperimental studies with a measure of within‐individual change were scored as very high and randomized controlled trials were scored as excellent.

Regarding measures of radicalization and family factors, only studies where all reliability coefficients were above 0.75 were scored as higher quality. Thus, studies where some family factors had reliability coefficients below 0.75 and other family factors had reliability coefficients above 0.75 were scored as lower quality (e.g., Jahnke et al., 2021). Quality of measures in studies which measured only demographic factors such as marital status (and similar) was also rated as higher because it was not reasonable to expect reliability coefficients of such measures. Regarding methodology used for studying risk/protective factors, only studies that used longitudinal data for all the family factor‐radicalization analyses were considered longitudinal, while studies that used some longitudinal and some cross‐sectional data were considered cross‐sectional (e.g., Clemmow, 2020). Marital status and similar variables were considered as fixed factors and if they were the only family‐focused variables used in a cross‐sectional design, they were considered to necessarily precede the outcome as suggested by Murray et al. (2009). In such cases, studies were coded as prospective longitudinal (e.g., Delia Deckard & Jacobson, 2015). In relation to causal risk/protective factors, studies with no comparison group were excluded. As stated by Murray et al. (2009), studies were considered to have an adequate control of confounders or change only if confounders were measured before the risk factor, not at the same time as, or after, the risk factor.

Following Cochrane recommendations (Page et al., 2017), the assessment of overall quality was done taking into account different evaluated domains. If quality was rated as low in at least one domain, a study was rated as lower quality. The Cambridge Quality Checklist includes three domains: correlational quality (including sampling, response rates, sample size and reliability of measurement tools), methodology to determine if a factor can be considered as a risk or protective factor, and the causality domain (Murray et al., 2009). Finding studies that determined causality was highly unlikely because, according to the Cambridge Quality Checklist, these would require an analysis of change and confounders measured before the risk factors. Thus, we decided to evaluate the overall quality based on the correlational domain and risk/protective factor methodology. Causal risk/protective factors were still rated to show possible differences in this criterion among the studies, but they were not used for the overall quality assessment. Following Jolliffe et al. (2012), studies were rated as higher quality in the correlational domain if they were rated as high in at least three out of the five criteria (otherwise, they were rated as low). If authors did not provide information on an item (e.g., reliability coefficients in a measure of a family factor) the criterion was rated as “unclear.” Regarding risk/protective factor methodology, studies were rated as high only if they were scored as high (longitudinal prospective designs and fixed factors). Thus, studies were rated as of overall higher quality if they were rated as high in at least three out of five criteria in the correlational domain and high in the risk/protective factor domain. We did not include a medium quality category because we did not expect to find enough studies to include three categories in moderator analyses.

#### 4.4.1 Measures of effect

This section pertains only to measures of effect for review Objective 1 (risk and protective factors), as no studies were identified for Objectives 2 and 3. Under Objective 1, some primary studies provided unadjusted bivariate coefficients that showed direct relations such as correlations. Other primary studies provided adjusted multivariate coefficients that controlled for confounders such as statistics derived from regression models. Some primary studies provided both adjusted and unadjusted coefficients. Although there is a debate in the field on whether unadjusted or adjusted coefficients are the most appropriate for meta‐analyses (Hunter & Schmidt, 2004) both have advantages and disadvantages. Nevertheless, only adjusted coefficients were included in meta‐analyses given a great variety of factors controlled for in the primary studies that made them difficult to compare. Given the low number of studies per risk/protective factor, if only adjusted results were available in a study, results were included in the unadjusted analyses to provide the most comprehensive analysis possible. If more than one model was available, adjusted coefficients were extracted from the model with the lowest number of covariates to obtain the least adjusted coefficients possible. Sensitivity analyses showing only unadjusted results were run.

Statistics needed to calculate the effect sizes were extracted from primary studies. For unadjusted analyses, these statistics mostly included coefficients such as Pearson's *r*, means, standard deviations, and the number of participants with and without the factor in the radicalized versus non‐radicalized groups. For adjusted analyses, statistics mostly included Bs, standard errors, betas and sample sizes. If statistics necessary to calculate effect sizes were unavailable in the published studies, authors were contacted and asked to provide all the details necessary for the meta‐analysis.

All the effect size calculations were performed using Comprehensive Meta‐Analysis Software (Borenstein et al., 2006). Given that most of the studies used correlation coefficients, standardized *r* using the Fisher's Z‐*r* transformation and variance were used as the effect size. Other statistics found among the primary studies such as means, standard deviations and sample sizes commonly used in group comparisons were transformed into *z* (equivalent to *r*). Transformations were done using Comprehensive Meta‐Analysis Software based on formulas by Lipsey and Wilson (2001).

Conversion from *d* to *r* was done as follows:

$$r=\frac{d}{\sqrt{d^{2}+a}},$$

$$a=\frac{\left(n_{1}+n_{2}\right)2}{n_{1}n_{2}}.$$

For the calculation of adjusted effect sizes, the standardized regression coefficient was used together with their associated standard errors as the basis for the inverse variance weight rather than the weight based on sample size.

Where the independent variable was dichotomous (e.g., divorced parents), effect sizes were calculated as

$$d=\frac{B}{\left(\mathrm{dependent}\;\mathrm{variable}\right)},$$

where *B* is the unstandardized regression coefficient from an OLS regression model (not from other model types).

For logistic regression with dichotomous independent variables, the following equation was used (Lipsey & Wilson, 2001; p. 202):

d=ln(OR)0.5513,

where the OR was the partial OR in the model or exp(*B*).

A *z* above 0 indicated that higher scores in a factor were related to high scores in radicalization. These factors were thus considered as risk factors. A *z* below 0 indicated that low scores in a factor were related to high scores in radicalization. These factors were thus considered as protective factors. A *z* of 0 (or confidence intervals that included 0) indicated no evidence of relation between variables.

### 4.5 Dealing with missing data

Some primary studies may have missing data, such as descriptions of the results without including the statistics necessary to calculate effect sizes. It is also possible to find studies that include incomplete information with some statistics that are insufficient for the calculation of the effect sizes (e.g., means without standard deviations or other coefficients that could be used for the calculation of the effect size). It is common to find studies that only provide significant results and do not provide the coefficients if they are nonsignificant (e.g., only marking them as ns). If any of these issues were present, authors of the primary studies were contacted and asked to provide the missing results or the databases for their calculation.

When authors of the primary studies could not provide the missing results, studies that only included descriptions of the findings without numerical results and studies that included incomplete information that was insufficient for the calculation of the effect sizes were excluded from statistical analyses (e.g., Cragin et al., 2015), but included in the summary tables. One study (Hagan et al., 1999) provided information for the calculation of the effect sizes of the significant results but did not provide information for the calculation of the effect sizes of the nonsignificant results. The direction and the sample sizes were known and, following Wilson et al. (2001), it was assumed that the effect was between zero and the smallest significant effect size. This means that the midpoint value was used. Sensitivity analyses were run to confirm that the weighted effect size was not substantially affected by this imputation.

### 4.6 Assessment of reporting biases

Publication bias was assessed through trim and fill analysis focused on the funnel plot asymmetry that was identified and corrected for. In trim and fill, the small studies that caused asymmetry in the funnel plot were “trimmed,” the center of the funnel plot was estimated, and the missing studies were “filled” around the center (Higgins et al., 2019). This method provides the number of missing studies and an adjusted effect size. Also, “one study removed” analyses were run to check if any particular study was the main source of significant effect sizes.

### 4.7 Data synthesis

Data were synthesized through a meta‐analysis conducted with Comprehensive Meta‐Analysis software (Borenstein et al., 2006). Analyses were based on Lipsey and Wilson (2001). Separate meta‐analyses were conducted for different family related risk and protective factors. After a thorough analysis of the primary studies, variables that were theoretically similar were grouped in categories for the meta‐analyses. At least two primary effect sizes were required to perform each meta‐analysis.

Statistics needed for the meta‐analysis were extracted from primary studies and entered in the software. Then, statistics from each individual study were transformed to a common effect size (Fisher's *z*) that was chosen based on the most commonly used analyses among the included studies. An overall effect size was calculated for each risk factor and protective factor if at least two studies were available. If some studies to be included in the same meta‐analyses measured the relation between high scores in a family factor and radicalization, and other studies measured the relation between low scores in a family factor and radicalization, coefficients were reversed to have the same meaning. The random effects method was used for data synthesis as the studies were heterogeneous. A common forest plot was then created to depict all the computed effect sizes.

### 4.8 Subgroup analysis and investigation of heterogeneity

Cochran's *Q* and *I* ^2^ were used to assess heterogeneity. Although *Q* is the traditional test to assess heterogeneity in meta‐analyses, it can be underpowered when the number of studies included in a meta‐analysis is low. Thus, *I* ^2^ was also calculated to assess heterogeneity. *I* ^2^ shows whether the variability across studies can be attributed to real differences or to chance (Higgins et al., 2002, 2003). The test of heterogeneity with *p* value < 0.10 combined with an *I* ^2^ value of 30% or greater shows evidence of heterogeneity across studies (Higgins et al., 2019).

Although we planned to run meta‐regressions, the number of studies included in each meta‐analysis was too low. Thus, moderator analyses were performed. Moderators included the publication year, location, Muslim versus non‐Muslim participants, age group, type of radicalization (cognitive vs. behavioral), ideology (Islamist, right‐wing and left‐wing) and quality score. Among these moderators, gender, age, location, and study design were defined *a priori* and Muslim versus non‐Muslim participants (the only available ethnic‐cultural comparison), type of radicalization and ideology were defined *a posteriori*. This was done because risk and protective factors were expected to differ among different levels of each moderator.

### 4.9 Sensitivity analysis

Sensitivity analyses were performed where missing data were imputed as explained in “dealing with missing data” section. Meta‐analyses that included some adjusted coefficients were run also after excluding the studies that did not provide bivariate coefficients to compare the results.

Given that many studies included several outcomes, sensitivity analyses were run to check if treating these outcomes as correlated with a maximum possible correlation (*r* = 1) versus treating them as independent affected the results. Treating multiple outcomes as correlated with a maximum possible correlation or as independent are the only two options available in Comprehensive Meta‐Analysis Software (Borenstein et al., 2006).

## 5 DEVIATIONS FROM THE PROTOCOL

There were three minor deviations from the protocol based on the information found in the primary studies. One was related to the definition of radicalization used in this systematic review, the second deviation was related to the analysis of adjusted results, and the third was the use of some additional sensitivity analyses.

Regarding the definition of radicalization, according to the protocol (Zych & Nasaescu, 2021), we planned to include any outcome focused on extremism, violent extremism, radicalization, radicalization to violence, and/or terrorism. Nevertheless, some of the located studies measured radicalization in a way that differed greatly from the majority of the included studies and needed to be excluded. For example, Rico and Jennings (2016) measured radicalization in terms of feeling Spanish, Catalan or both, Krout and Stagner (1939) classified participants self‐identified as socialists in the radical group, and Førland et al. (2010) defined radicalism as “a mindset characterized by opposition to the established order in capitalist societies” (p. 828) including items such as being “anarchist, anti‐authoritarian, communist, hippie, Maoist, Marxist, pacifist, radical, socialist, student rebel and youth rebel” (p. 828) in the sixties. Several studies focused on voting for radical or populist parties (e.g., Coffé & Voorpostel, 2010). There were also some studies that measured radicalization mixed with other constructs such as right‐wing extremism mixed with xenophobia where participants who reported either were classified as extremists (e.g., Baier et al., 2010), religious extremism mixed with women's rights and other constructs (El‐Badayneh & ElHasan, 2017) or right‐wing extremism measured through three variables used as a proxy including readiness to use violence (in general), xenophobia and national/authoritarian attitudes (e.g., Klein‐Allermann et al., 1995). Thus, following Wolfowicz et al. (2021), only studies that defined radicalization as a use of violence or illegal action to defend a cause were included. These studies could focus on extremism, violent extremism, radicalization, radicalization to violence, and terrorism. We also included studies that measured support for groups that use violence to defend a cause such as neo‐Nazi (e.g., Siedler, 2006 who measured participation in skinhead neo‐Nazi groups) and studies that measured support for terrorism (e.g., European Values Study, 2008 where participants were asked if terrorism can be justified), assuming that terrorism as such is always violent or illegal. We excluded studies where radicalization measures were not “pure” and mixed with other constructs.

The second minor deviation from the protocol refers to the analysis of adjusted results that were planned to be meta‐analyzed separately to check if the results would hold after controlling for covariates. Nevertheless, the current study only included meta‐analyses of unadjusted results when available, adding adjusted coefficients when unadjusted coefficients were not available and running sensitivity analyses to check if the inclusion of adjusted coefficients affected the results. We decided not to run separate adjusted analyses after a careful examination of the primary studies and verifying that the number of projects that included both adjusted and unadjusted coefficients was relatively low and coefficients controlled for differed greatly among the studies, making it difficult to produce meaningful comparisons.

The third minor deviation was an inclusion of additional sensitivity analyses. Given that many studies included several outcomes that were combined, a conservative approach was used treating these outcomes as correlated with a maximum correlation possible. Sensitivity analyses were run to check if the results differed when outcomes were assumed to be independent.

Methods specified for other objectives (coding schemes developed for data extraction, assessment of risk of bias in family‐focused interventions, and measures of effect in family‐focused interventions) were not utilized as no studies were included that met the criteria for these objectives. Future updates of the review will follow the methodology specified in the protocol (Zych & Nasaescu, 2021) should studies meeting the criteria for the objectives be identified.

## 6 RESULTS

### 6.1 Description of studies

#### 6.1.1 Results of the search

A total of 121,997 studies were located through searches in databases, hand searches in journals, gray literature searches, harvesting of references from included studies, and contact with experts. When possible, references were exported to EndNote and 35,406 duplicates were eliminated before title/abstract screening. Thus, 86,591 studies were screened at the title/abstract stage.

Out of the located studies, 86,307 were screened as irrelevant and off‐topic according to our title and abstract screening. Thus, 284 studies were retained for the full text screening. Among these studies, 9 were duplicates that were not eliminated at the previous stages and were therefore excluded. Finally, 275 studies were included for full text eligibility screening.

Full text screening was conducted using the eligibility screening form (see Table 1). Among the 275 studies included for the full text screening, 117 were excluded because they did not provide empirical quantitative data, 71 were excluded because they did not measure or report the relation between radicalization and family‐related factors or intervention, 27 were excluded because they did not provide a comparator (i.e., non‐radicalized group from the general population or variation in the degree of radicalization) and did not report intervention results. Twenty‐one studies were excluded because they did not meet the definition of radicalization (support for or use of violence to defend a cause, including support for radical groups or terrorism), and four studies were excluded because they were based on a duplicated data set without providing any additional information. Thus, 35 documents (and 33 studies) were included in our systematic review, among which, 30 studies provided sufficient data for the calculation of effect sizes.

**Figure 1:**
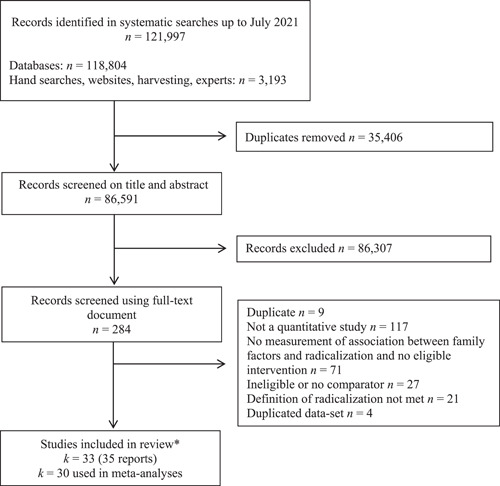
PRISMA flow diagram

Given that no studies focused on family‐related consequences of radicalization or intervention programs against radicalization were included, the following sections focus only on studies about family‐related risk and protective factors for radicalization (Figure 1).

#### 6.1.2 Included studies

A total number of 33 studies published in 35 documents was included in the systematic review. Table 3 includes detailed description of the studies (types of documents and countries where the study was conducted), participants and procedure (number of participants, age, percentage of males and females, ethnic‐cultural background, SES, recruitment strategy, settings where recruited, response rates, and design—cross‐sectional or longitudinal—and procedure for data collection). It also summarizes family factors included in each study and their measurement approach (names of the factors, description of questionnaires or items used for data collection including numbers of items, examples of items per scale, response scale, reliability data and if the instruments were self‐reports or other reports), and terms used for radicalization and their measurement approach (same information as for family factors). Only information reported in each study is included in the table, and some information is missing. For example, the table includes information about response rates in some studies, but this information was not included in other studies, and it is not shown in the table.

**Table 3:** A summary of the included studies with details on participants and procedure, family factors and their measurement tools, terms used for radicalization and their measurement tools.

Study citation, type, and country	Participants and procedure	Family factors and their measurement tools	Terms used for radicalization and its measurement tools
Acevedo and Chaudhary (2015) Journal article USA	Participants (*n* = 1050): Age: All above 18 years old (*M*_age_ = 41.12, SD = 14.62) Gender: 53.32% male; 46.68% female Ethnicity/culture: All participants were Muslim including 15.68% Shi'a, 62.24% Sunni, and 22.07% other SES: 11.10% poor, 35.10% fair, 87.45% good, 12.55% excellent Education: 6.76% less than high school, 17.73% high school diploma, 23.52% some college, 27.02% college, 24.97% postgraduate Procedure: Participants selected through random sampling of households for a national telephone survey (response rate of 79%). The study was cross‐sectional.	Marital status (no other details specified).	*Terrorism* defined as a form of politically motivated violence measured through one self‐reporting item: “Some people think that suicide bombing and other forms of violence against civilian targets are justified to defend Islam from its enemies. Other people believe that no matter what the reason, this kind of violence is never justified. Do you personally feel that this kind of violence is often justified to defend Islam?” coded as “often, sometimes, or rarely justified” (=1) versus “never justified” (=0).
Abdi (2019) PhD thesis USA, Canada	Participants (*n* = 279): Age: 18–30 years old (*M*_age_ = 24.36) Gender: 54.7% males and 45.5% females Ethnicity/culture: Somali or with at least one Somali parent. Procedure: Participants selected through convenience sampling in a community setting. The study was cross‐sectional, and data were collected through interviews in community settings conducted by a researcher.	*Family conflict* such as arguments measured through the Family Conflict Scale (Moon et al., 2009) that includes three self‐reporting items (no examples given) focused on family conflict, answered on a 4‐point response scale.	*Openness to violent extremism* understood as the support for violence to achieve political goals measured with an adapted version of Activism and Radicalization Intentions Scale (ARIS; Ellis et al., 2015; Moskalenko & McCauley, 2009), including a self‐reporting radicalization scale with five items (e.g., “I can understand someone who would participate in a public protest against oppression of her/his people even if she/he thought the protest might turn violent”). Items were answered on a 7‐point Likert scale.
Altunbas and Thornton (2011) Journal article UK	Participants (*n* = 1,440 including 1,363 from general population and 77 terrorists): Age: General population *M*_age_ = 32.84, SD = 9.71, 77 terrorists *M*_age_ = 26.35, SD = 6.26. Gender: Among general population, 49.6% were male, among terrorists, 96.1% were male. Ethnicity/culture: All participants were Muslim. Among general population, 8.64% were African, 2.31% Mixed South Asian‐White, 11.5% Indian, 56.48% Pakistani, and 10.80% other. Among terrorists, 29.9% were African, 23.4% Mixed South Asian‐White, 3.9% Indian, 27.3% Pakistani, and 3.9% other. SES: Among general population, 48.26% were employed, among terrorists, 37.66% were employed. Procedure: General population participants selected through random sampling of households and interviewed face‐to‐face. Terrorists' data extracted from documents analysis. The study was cross‐sectional.	*Marital status* recorded through an analysis of documents such as Google searches and UK Home Office reports in the case of terrorists. Self‐reporting questions from the annual British Crime Survey for general population of UK Muslim (details not specified).	Homegrown UK Islamist terrorism measured as conviction or death in an inflicted terrorist attack based on Gartenstein‐Ross and Grossman (2009), UK Home Office documents, Wikipedia, Google searches and press records documents.
Baier et al. (2016) Journal article Germany	Participants (*n* = 11,003): Age: ninth graders (equivalent to around 14 years old) Gender: 50.9% male. Ethnicity/culture: Sample representative for Lower Saxony, 5055 participants without a migration background, 390 Muslim with a migration background. Procedure: Participants selected through random selection in schools (response rate of 63.7%). A written survey conducted in schools by trained test managers. The study was cross‐sectional.	*Parental unemployment/poverty* measured with an item “father and/or mother currently unemployed or recipients of social assistance,” *parental violence* measured with 12 items (e.g., hit with an object; Baier et al., 2009), *parental care* measured with six items (e.g., mother/father praised me when I did something well, Baier & Pfeiffer, 2011). All self‐reports.	*Right‐wing extremist behavior* measured with seven items (Baier & Pfeiffer, 2011) focused on violent behavior against foreigners, migrants and left‐wing people, answered on a yes (1) no (0) scale (e.g., painted or sprayed a swastika or a word like foreigners out on the wall of a house or a toilet). *Left‐wing extremist behavior* measured with eight items on violent behavior committed against right‐wing individuals or other political opponents (e.g., hit and hurt someone for being right wing). *Islamic extremist behavior* measured with two items focused on hitting someone because they were German and destroying things because they were German (items answered only by Muslim participants with migration background). All self‐reports.
Berger (2016) Journal article UK, France, Germany, Spain	Participants (*n* = 1,627): Age: 33 years old in France, 32 years old in Spain, 37 years old in Germany, 33 years old in the UK. Ethnicity/culture: All participants were Muslim. SES: Monthly income: France: €1450–1900, Spain: €601–1200, Germany: €1450–1900, UK: £1666–2499. Procedure: Participants randomly selected in households and interviewed via telephone (UK, Germany and France) or face‐to‐face (Spain). The study was cross‐sectional.	Marital status (single), no further details were specified.	Radical political viewpoints measured with two self‐reporting questions including support for “suicide bombings against civilians in the name of defending Islam” and “confidence in Osama Bin Laden” coded as 0 (never) and 1 (other)
Bhui, Everitt, et al. (2014), Bhui, Warfa, et al. (2014) Bhui et al. (2016) Journal article UK	Participants (*n* = 608): Age: 18–45 years old Gender: 54.45% men and 45.55% women Ethnicity/culture: 46.65% Pakistani, 53.35% Bangladeshi, all of Muslim heritage living in the UK SES: 50.26% employed, 20.97% unemployed, 28.78% retired/ill/housewives. Regarding education, 19.62% had no qualifications, 50.43% < Bachelor degree, 29.95% Bachelor, Master, PhD. Income: 21.86% < 5000 GBP, 50.51% 5000–24,999 GBP, 11.07% 25,000–49,999 GBP, 6.56% > 50,000 GBP. Procedure: Households selected through quota to resemble the population. The study was cross‐sectional and surveys were conducted in a computer‐assisted format.	*Marital status* measured with a question (single, married or divorced). *Death of a partner, spouse, parent or a child*, and *separation due to marital differences* were measured as a part of adverse life events in the previous 12 months self‐reporting questionnaire with yes/no answer.	*Sympathies for violent protest and terrorism* understood as “a ‘pre‐radicalization’ phase when individuals are vulnerable to recruitment to terrorist causes” measured through a self‐reporting scale developed by authors, with 16 items where participants were asked to report their sympathy or condemnation of extreme acts (e.g., use of suicide bombs to fight injustice or commit terrorist acts) responded on a 7‐point Likert scale (*α* = 0.81). Participants were classified in three clusters through a cluster analysis, including the least sympathetic, an intermediary group, and the most sympathetic groups.
Boehnke (2017) Journal article Germany	Participants (*n* = 147 students, *n* = 147 their mothers and *n* = 147 their fathers): Age: Students: *M*_age_ = 20.5 (SD = 2.09); mothers *M*_age_ = 45.6 (SD = 5.75); fathers *M*_age_ = 48.1 (SD = 5.75); Gender: students: 80.27% females, 19.73% males; parents: 50% females, 50% males. Procedure: Convenience sampling in a university (essentially sociology and psychology) including students and their parents. This was a cross‐sectional study in which parents and students filled in questionnaires at home and returned them in an envelope.	*Parental hierarchical self‐interest* defined as the extent to which people engage in an “elbow mentality,” including constructs such as Machiavellianism, acceptance of social inequality, individualism, materialism and competitive orientation. Hierarchical self‐interest included five constructs that were summed up, with items authored by Hagan et al. (1999) and Hadjar (2004). Machiavellianism included eight items (e.g., “It is not so important how you win, but that you win”) and its *α* were =0.78 (mothers) and =0.79 (fathers). The acceptance of social inequality included three items (e.g., “The differences in rank between people are acceptable because they essentially express what you made of the opportunities you had” and its *α* were =0.65 (mothers) and =0.61 (fathers). Individualism had three items (e.g., “Everyone would be better off if everyone just took care of themselves” with *α* = 0.51 (mothers) and =0.33 (fathers). Materialism included three items (e.g., “The most important thing in life is achievement” with *α* = 0.66 (mothers) and =0.68 (fathers). Competitive orientation with five items (e.g., “To me, being successful in life means being better than others” with *α* = 0.83 (mothers) and =0.82 (fathers). Items were answered on a 5‐point Likert scale. The total *α* were =0.68 (mothers) and =0.63 (fathers). Family factors measured through self‐reports (by parents). *Parental right‐wing extremist behavioral tendencies* measured with the questionnaire described in terms used for radicalization and its measurement tools.	*Right‐wing extremist behavioral tendencies* measured with 10 items, five for right‐wing extremist behavior (e.g., listening to extremist music bands, going out with extremists, insults towards foreigners; *α* for students = 0.67, for mother/father = 0.47) and five for anti‐right‐wing behavior (e.g., defending a foreigner, participating in anti‐right protests, erasing swastika if found on a wall; *α* for students = 0.58, mothers = 0.54, and fathers 0.55). Items were answered on an 11‐point Likert scale. Factor structure confirmed through a confirmatory factor analysis. Self‐reports (by children and parents).
Boehnke et al. (1998a; 1998b) Journal article Germany	Participants (*n* = 590 in two waves): Age: Grades 7 and 9, followed up one year later Gender: proportion of girls and boys “close to equal” Ethnicity/culture: 342 from East and 248 from West Berlin, “socially heterogeneous.” Procedure: Participants recruited and surveyed in schools, response rate = 65%, attrition of 27.6%. Study conducted with a longitudinal design, with a 1‐year follow‐up. Parental control measured at wave 1, radicalization at wave 2 (1 year afterwards). Stratified random cluster sampling.	*Parental control* measured with self‐reports including four yes/no items (e.g., being allowed to meet friends after 8 p.m., staying overnight in a friend's house without asking parents); *α* = 0.66 and 0.73 for East and West Berlin, respectively. Unifactorial model confirmed through a Confirmatory Factor Analysis.	*Rightist extremist attitudes* included four items answered on a 4‐point Likert scale with “typical political slogans of the German right” considered to be neo‐Nazi (e.g., “Germany, the only true future” and “Fuhrer command, we will follow”); *α* = .70 and 0.72 for East and West Berlin, respectively. Factor structure confirmed through a confirmatory factor analysis. Self‐reports.
Cherney and Murphy (2019) Journal article Australia	Participants (*n* = 800): Age: *M*_age_ = 34.89, SD = 15.51 Gender: 49.5% female Ethnicity/culture: All Muslim with 57.9% born in Australia SES: Mean income = $56,000–$60,000; educational status *M* = 5.31, SD = 2.02 with 0 = no schooling and 10 = post‐graduate degree. Procedure: Participants recruited through a random selection in households (response rate of 18%). Muslim trained interviewers interviewed the participants in public locations (e.g., café, library). The study was cross‐sectional.	*Marital status* measured through a self‐report and coded as 0 = not married; 1 = married.	Muslims' passive support for terrorism measured with a self‐reporting question “Terrorists sometimes have valid grievances” responded on a Likert scale ranging from 1 = strongly disagree to 5 = strongly agree.
Clemmow (2020), Clemmow et al., (2020) Research degree thesis and journal article Lone‐actor terrorists who planned their attacks in the US, UK, Europe, or Australia; general population in the US, UK and Western Europe	Participants (*n* = 125 lone‐actor terrorists and *n* = 2108 general population): Age: General population sample 18–50 years old (*M* = 30.06, SD = 8.43), terrorists *M*_age_ = 33.56 (SD = 19.91). Gender: General population: 54.9% females, 45.1% males, terrorists: 97.6% males, 2.4% females. Ethnicity/culture: Among the terrorists, 48.8% were US citizens. Among general population, 52.1% were UK residents, 28.4% were US residents, and 19.5% were Western European residents. SES: Among terrorists, not reported. General population subjective socioeconomic status on a scale from 1 to 10 of *M* = 5.2. General population education level: 1.7% no formal qualification, 16.8% secondary education, 28.7% college A Level, 33.5% undergraduate degree, 16.5% graduate degree, 2.7% doctoral degree. General population employment status: 45.4% full time, 29.4% part time, 2.6% due to start a new job, 14% unemployed, 9.4% not in paid work, and 8.7% other. Procedure: Sample recruited through convenience sampling on the Internet. The study was mainly cross‐sectional, but data for lone‐wolf terrorists were coded based on different sources, including past and present factors. For lone‐wolf terrorists, data were extracted from different documents by three coders. For general population, surveys were administered online using a sample recruited via an online panel.	Family factors measured were: *Grew up in an abusive home, having children, single, grew up in a religious household, family death, spouse involved in wider movement, perpetrator of domestic abuse in adulthood*. For lone‐wolf terrorists, data were extracted from different documents such as sworn affidavits, court reports, first‐hand accounts, news reports via LexisNexis, biographies and scholarly articles. Variables were coded as present or absent. For general population, participants were asked self‐reporting questions derived from the terrorist codebook that were answered as yes/no.	*Lone‐actor terrorists* defined as “an individual who carried out or planned to carry out, alone, an attack in service of some form of ideology, for which they were convicted or died in the attempt.” For lone‐actor terrorists, instruments were not reported.
Cragin et al. (2015) Report Palestinian territories	Participants (*n* = 600): Age: 18–30 years old. Gender: 51.2% male and 48.8% female. Ethnicity/culture: Diverse ethnic‐cultural backgrounds, probably representative for the population given the multistage probability sample. SES: 75% beyond secondary education and 25% with secondary education or above, 49% employed and 50.8% not employed, housewives or students. Procedure: Sample randomly selected from the community with a response rate of 88%. Questionnaires administered cross‐sectionally, face‐to‐face in households by a local research firm.	*Family arrested or detailed* by Israeli security forces measured with direct questions. *Social ties with parents* were measured by asking about the extent to which parents had influence over participants' major life decisions. All self‐reports.	Openness to political violence operationalized through two variables including *attitudes toward suicide attacks against civilians* and *willingness to engage in violent protests*, both measured through a self‐report with a “series of questions” (e.g., “Do you support or oppose suicide attacks against Israeli civilians” in case of the former and “Is it likely or unlikely that you would ever engage in violent protests?” in case of the latter, both answered on a 4‐point scale).
Delia Deckard and Jacobson (2015) Journal article The United Kingdom, Germany, and France	Participants (*n* = 1200): Age: *M*_age_ = 40.39 (SD = 13.71) ranging from 18 to 82. Gender: 52% females, 48% males. Ethnicity/culture: All Muslims. SES: Self‐identified as poor or nearly poor (1.5%), just getting along (20.2%), living reasonably comfortably (47.2%), living very comfortably (26.4%), and prosperous (4.7%). Procedure: Participants selected through randomly dialed/randomly selected telephone numbers, with most interviews in private, and some with another person present. A response rate of 13%. Data collected cross‐sectionally over the phone by a survey company “which subcontracted to local market research firms in each country.”	*Marital status* measured by asking the participants if they were married (coded as 1) or non‐married (coded as 0).	*Radicalism (Islamist)* defined as willingness to engage in violence, self‐reported through a single question: “When do you think resorting to violence is justified? To defend your faith?” with a dichotomous yes/no response.
Dhumad et al. (2020) Journal article Iraq	Participants (*n* = 313): Age: *M*_age_ = 34.06 (SD = 9.79) Gender: 100% males. Ethnicity/culture: All from Iraq SES: Illiterate – 7.72%, primary education – 27.97%, secondary education – 21.54%, preparatory – 14.15%, and university – 28.62%; 96.43% employed. Perceived financial situation: very poor – 7.23%, poor – 23.68%, average – 45.07%, good – 23.03%, and very good – 0.99%. Procedure: Participants selected through convenience sampling. Terrorism convicts (*n* = 160) were recruited in prisons, control community participants (*n* = 88) were recruited “through networking.” A response rate of 66%. Data collected cross‐sectionally through interviews conducted by researchers in private locations in the community or within the prisons.	*Marital status, number of children, family size, authoritarian father, family disintegrated, family member murdered, harsh treatment by parents or others* “Data were collected using a semi‐structured tool designed for the study.” No details on how family factors were measured are provided, these factors are just mentioned as a part of this tool administered as a self‐report.	*Terrorism* defined as a conviction under article 4 of the Antiterrorism Law including “killing civilians (*n* = 60), affiliation with terrorist groups (*n* = 27), planting bombs (*n* = 13), and killing government officials or Iraqi war‐fighters (*n* = 10) and other offences (*n* = 30)” Measured through official records of conviction of terrorism.
European Values Study (2008) Data set Europe (47 countries)	Participants (*n* = 66,281 participants): Age: *M*_age_ = 46.80, SD = 17.80, ranging from 15 to 108 Gender: 44.4% male and 55.6% female Ethnicity/culture *and* SES: Samples are representative for each country Procedure: Participants were randomly selected in households. The study was cross‐sectional and conducted mostly through face‐to‐face surveys by trained interviewers.	*Importance of the family* measured through a question: “How important is family in your life.” *Marital status* indicated in the survey, *number of children* indicated in the survey, *divorce of own children* measured with “have you experienced divorce of own children,” *parental divorce/single parent family* included two items (“have you experienced divorce of own parents,” and “lived with both parents at age 14”), *divorce of other relatives* measured with “have you experienced divorce of a relative,” *family‐member death* (including “have you experienced death of father,” “have you experienced death of mother,” “have you experience death of own children”), *spouse born abroad* measured with “spouse born in (country),” indicated *education level of a partner*, *unemployment of spouse/partner* measured with “spouse/partner experienced unemployment longer than 3 months,” *parental education* measured with “highest education attained by father/mother,” *parental unemployment* measured with “father/mother employed at age 14,” *parents reading books* measured with “mother liked to read books” and “father liked to read books,” *discussing politics with parents* measured with “discussed politics with mother” and “discussed politics with father,” *parents liked to follow the news* measured as “mother liked to follow the news” and “father liked to follow the news,” *parental poverty* measured as “parents had problems making ends meet” and “parents had problems replacing broken things.” All self‐reports.	*Support for terrorism* measured through a self‐reporting question “Terrorism is everyday news. In principle, most people are against it, but there is still room for differences of opinion. Which of these two statements do you tend to agree with?" "There may be certain circumstances where terrorism is justified” and “Terrorism for whatever motive must always be condemned” (also neither, don't know, and no answer).
Fair and Hamza (2018) Journal article Pakistan	Participants (*n* = 14,508): Age: 35.84% were between 18 and 29 years old, 49.71% were between 30 and 49 years old, 14.31% were 50+ years old and 0.14% did not respond Gender: 41.32% were females and 58.68% males Ethnicity/culture: Punjabi (32.86%), Muhajiir (7.06%), Pashtun (34.82%), Sindhi (9.66%), Baloch (10.47%), no response/don't know (0.58%), other (4.56%). Type of Madrassah: Shia (4.14), Sunni (50.96), Deobandi (40.86%), Ahl‐hadith (4.03%) SES: Level of education: less than Primary (38.68%), Primary (11.95%), Middle (13.34%), Matriculate (17.97%), Higher Education (17.18%), don't know/no response (0.88%). Income quartiles: First quartile (35.74%), second quartile (27.16%), third quartile (12.43%), fourth quartile (19.07%), don't know/no response (5.6%). Procedure: The sample was randomly selected and interviewed in households by a major survey firm and the research team (response rate of 71%). The study was cross‐sectional.	*Marital status* including single/never married, married, divorced, widowed, don't know/no answer measured through a self‐report.	*Support for Islamist terrorism* defined as support for two militant groups in Pakistan including Sipah‐e‐Sahaba‐e‐Pakistan (SSP) and Afghan Taliban. Participants were asked “How much do you support Sipah‐e‐Sahaba‐e‐Pakistan (SSP) and their actions?” and “How much do you support the Afghan Taliban and their actions?” answered on a 5‐point scale (“not at all,” “a little,” “a moderate amount,” “a lot,” or a “great deal”).
Goede et al. (2020) Report Germany	Participants (*n* = 6,715): Age: *M* = 14.7 years (all in Grade 9) Gender: 52.6% females; 47.4% males Ethnicity/culture: 43.5% had a second‐generation migration background, 92.8% were born in Germany, 85.4% with German citizenship; 31.3% did not belong to any religion, 28.2% were Protestant, 18.9% were Catholic, 3.4% other Christian denomination, 14.8% were Muslim and 0.6% were Jewish SES: 7.8% received welfare payment, 81% reported a good self‐perceived financial situation and 19% reported a bad self‐perceived financial situation Procedure: The study was cross‐sectional and, although researchers intended to include schools in each federal state, difficulties in recruiting participants at different levels (federal, schools, parental consents) led to what could be described as convenience sampling. Participants filled‐out questionnaires in schools on an internet‐enabled device supervised by a trained test administrator (response rate of 65%).	*Family cohesion* measured with four items (*α* = 0.82) based on Fok et al. (2014) focused on positive family climate (e.g., In our family, we help and support each other). *Parental control* measured with four items (*α* = 0.61) based on Bergmann et al. (2019) focused on parental interest and control (e.g., parents pay attention to how things are going at school). *Dealing with conflicts* based on Bergmann et al. (2019) and Fok et al. (2014) measured with five items (*α* = 0.74) focused on conflicts in the families (e.g., there are a lot of fights in our family). Six *critical family events* measured with one item each, including divorce, moving far away, accident, death of father, mother or another person (e.g., grandma, grandpa).	*Right‐wing extremism* measured with nine items (*α* = 0.86) focused on extremism with a clear rejection of legal norms (free democratic basic order) or humanitarian value systems (general human rights) or approval of deviating value and norm systems including support for violence (e.g., Leftists should not be surprised if they get hit). *Islamist extremism* measured with six items (*α* = 0.79) focused on extremism that contradicts the free democratic basic order or general human rights, including the endorsement of the totalitarian political ideology of the so‐called Islamic State (e.g., “It's a good thing when people go to Syria to join ISIS.”
Groppi (2018) PhD thesis Italy	Participants (*n* = 440): Age: 16–60 years old (263 16–30 years old, 155 30–60 years old) Gender: 69% males. Ethnicity/culture: All Muslim, 116 born in Italy, 209 born in Africa, 64 born in Asia, and 34 in other European countries. Participants lived in different parts of Italy. Among the participants, 365 were Sunnis, 21 Shia, 39 Sunni converts and 1 Shia convert. SES: 188 were employed, 59 business owners, 119 students, 36 unemployed and 30 other; 212 earned less than 1,000 Euros/month, 133 earned 1,000–2,000 Euros, 16 earned more than 2,000; 232 had a high school diploma, 113 a university degree, 88 neither. Procedure: This was a cross‐sectional study with convenience sampling in the community settings. Participants interviewed by the researcher at mosques, Islamic centers, and public places.	*Having children* measured with a question “Do you have any children?,” responded as yes/no.	*Islamist radicalization* measured with three items focused on violence in defense of faith, duty to punish offenders of Islam, Support for Al Qaeda and Support for ISIS, all responded on a 3‐point response scale.
Hagan et al. (1999) Journal article Germany	Participants (*n* = 2.229): Age: Grades 7–10; *M*_age_ in Berlin = 14.53 (SD = 1.15), *M*_age_ in Chemnitz/Siegen = 14.73 (SD = 1.21) Gender: 50.8% females. Ethnicity/culture: 545 from East/West Berlin and 1,684 from Chemnitz/Siegen Procedure: Random sampling in two study sites (two districts of East and West Berlin), not reported for other two study sites. Participants recruited and surveyed in schools from populations described as “demographically representative.” Response rates varied among the study sites from 59% to 71%. This was a cross‐sectional study.	*Parental control* measured through self‐reports with three yes/no items (e.g., being allowed to meet friends after 8 p.m., staying overnight in a friend's house without asking parents). *α* values not reported, but it is stated that instruments in general had *α* values between 0.60 and 0.80.	*Right‐wing extremism* measured through self‐reports with four items answered on a 4‐point scale including “provocative neo‐Nazi slogans,” (e.g., “Germany, the only true future” and “Fuhrer command, we will follow”). *α* values not reported, but it is stated that instruments in general had *α* values between 0.60 and 0.80.
Jahnke et al. (2021) Journal article Germany	Participants (*n* = 6,715): Age: *M*_age_ girls = 14.59, SD = 0.69; *M*_age_ boys = 14.71, SD = 0.74). Gender: 53% were girls Ethnicity/culture: 43%–44% with migration background Procedure: Participants were recruited in schools and answered an online survey during school hours. The study was cross‐sectional.	*Low family cohesion* defined as “families in which members do not feel committed to one another and fail to provide familial support” measured with four items “from the Cohesion subscale of the Brief Family Relationship Scale (Fok et al., 2014),” translated into German, answered on a 5‐point Likert scale (Cronbach's α = 0.82, 95% CI = [0.81, 0.82], McDonald's *ω* = 0.82, 95% CI = [0.81, 0.83]). *Parental violence* measured through five items focused on violent acts perpetrated by parents towards the child (e.g. “grabbed me violently or pushed me”) answered as yes/no (Cronbach's *α* = 0.68, 95% CI = [0.67, 0.69], McDonald's *ω* = 0.70, 95% CI = [0.69, 0.71]). All self‐reports.	Political violence support measured with a four‐item self‐reporting questionnaire from the Zurich Project on the Social Development of Children and Youths (Nivette et al., 2017) answered on a 5‐point Likert scale (*α* = 0.76). An example item is “It's sometimes necessary to use violence, commit attacks, or kidnap people to fight for a better world.”
Kuhn (2004) Journal article Germany	Participants (*n* = 1,309): Age: 18–20 years old Gender: 38% male, 62% female. SES: 44% had at least one parent who graduated from high school. 85% of the sample was in higher education, 15% left school for vocational training or work. Procedure: Sample recruited in schools and households (through mail). Participants who attended schools filled out a paper‐and‐pencil questionnaire in the classroom. Participants who did not attend schools were sent the questionnaire to their home via mail. The study was cross‐sectional.	*Subjective importance of parents* measured by asking how important their father and their mother were, on a 5‐point scale ranging from very important to completely unimportant. There was a strong correlation between these two items (*r* = 0.71). *Leisure time spent with parents* was measured by asking participants how frequently they spent leisure time with family which was responded on a 5‐point scale. *Conflict with parents in everyday life* was measured with nine items asking about disagreements (e.g., regarding “friends,” “going out in the evening,” “untidiness”), responded on a 5‐point scale ranging from never to very often (*α* = 0.75). *Communication with parents about politics* was measured with four items by asking how frequently participants talked about politics with father and mother, and how frequently they had arguments about politics with father and mother. Items were answered on a 5‐point response scale ranging from never to very frequently (*α* = 0.80). *Parental education* was coded as 0 = both parents low and 1 = at least one parent high. All instruments were self‐reports.	*Readiness to use violence in political action* was measured by asking the participants if they “would participate in the three following illegal or violent actions to protest against something or to call attention to something in the public: spray protest slogans; damage road signs, break windows or similar things out of protest; be violent, if required, at protest actions.” The questionnaire was answered on a 5‐point Likert scale (from “I would definitely not participate” to “I would surely participate”) dummy coded as 0 (definitely not participate) and 1 (all the others). This scale had a good Cronbach's *α* = 0.80. Both were self‐reports.
Manzoni et al. (2019) Report Switzerland	Participants (*n* = 8,317): Age: 55.8% aged 17 or 18, 22.5% younger Gender: 49.7% male and 50.3% female Ethnicity/culture: Participants with diverse ethnic‐cultural backgrounds including native Swiss and participants with migration background (52.1%) from Portugal (6.6%), Italy (5.7%), and Kosovo (4.4%). Among the participants, 40.5% were Catholics, 26.3% no religious affiliation, 13% Protestants, 9.6% Muslim, 3.4% Orthodox, 2.6% Evangelical, 2.4% other, and 0.8% Buddhist SES: 52% in vocational school, 12.3% in a technical school or vocational baccalaureate, 26.4% in grammar school and 9.3% in transitional training Procedure: Participants selected through random selection in schools (response rate of 39.1%) Data collected through a cross‐sectional standardized online survey administered in computer rooms at schools by teachers or interviewers.	*Parental education* measured with a question about the school leaving certificate or a degree for mother and father. *Parental affection* measured with three items (e.g., praised me when I did something well, *α* = 0.78), *parental control* measured with three items (e.g., parents know what I do when I am not at home, *α* = .85), *parental inconsistency* measured with three items (e.g. parents promised to bring something and then they did not, *α* = 0.63), all answered on a 5‐point Likert scale. *Violence between parents* measured with three items (e.g., I have witnessed one parent shove or shake the other violently), *punishment by parents* measured with two items (e.g., hit me), and *severe parental violence* measured with two items (e.g., hit me with an object) answered on a 5‐point Likert scale (reliability not provided, combined punishment and parental violence into corporal punishment). *Critical family life events* measured by asking about separation, divorce, serious illness or death of parents. All self‐reports.	*Right‐wing extremism* including dimensions: nationalism, pro‐dictatorship, social Darwinism, racism, xenophobia, Muslim integrity, anti‐Semitism, and willingness to use violence against foreigners and left‐wing extremists. Each dimension was measured with one to three items responded on a 6‐point scale. *Left‐wing extremism* included dimensions: communism, anticapitalism, hostility towards the police and the state, and willingness to use violence against capitalists, police officers and right‐wing extremists. Each dimension included up to four items answered on a 6‐point response scale. *Islamist extremism* included dimensions: introduction of theocracy and Sharia, superiority of Islam, devaluation of western societies, hostility towards nontraditional Muslims, Swiss hostility, and willingness to use violence against non‐Muslims, Terrorism and IS. Each dimension included up to five items responded on a 6‐point scale. Reliability of the scales described as sufficient. All self‐reports.
McCauley (2011) Data set US	Participants (*n* = 429): Age: *M*_age_ = 46.81, SD = 17.37, ranging from 18 to 92 Gender: 52% males and 48% females Ethnicity/culture: White – 69.5%, Black – 11.4%, Other – 4.7%, Hispanic – 12.1%. 2+ races – 2.3%. SES: Education: less than high school—14.7%, high school—33.6%, some college—25.6%, Bachelor's degree or higher—26.1%. Own living quarters—66.2%, rent – 25.9%. Household income less than 30,000 USD—33.1%, between 30,000 and 60,000 USD—35.7%, more than 60,000 USD—31.3%. Procedure: Participants recruited through random selection of households (response rate of 72%) that were randomly dialed and provided with hardware and internet access to fill in the questionnaires online. The study was cross‐sectional.	*Marital status* including married, single, divorced, widowed, and separated, measured with a self‐reporting question about marital status.	*Future radicalism* measured with four questions focused on a self‐reported likeliness of future support of groups that use violence/illegal action to fight for a cause (e.g., “Participate in a public protest against oppression of your group if you thought the protest might turn violent?”), answered on a 4‐point scale (*α* = 0.84). *Past radicalism* measured with two self‐reporting questions focused on engaging in political activities through illegal/violent means (e.g., “Engaged in a political activity in which you suspected there would be a confrontation with the police or possible arrest” with yes/no answer, recoded as 0 (no to all) 1 (yes to at least one).
Moskalenko and McCauley (2009) Study 1: USA Study 2: Ukraine Journal article	Study 1 (USA), Participants (*n* = 140): Age: 17–33 years old. (*M*_age_ = 19.6, SD = 1.78) Gender: 86% females, 13% males Ethnicity/culture: 75% Caucasian, 8% East Asian, 5% African American and 6% were of mixed ethnicity; 29% atheists, 24% Protestant, 20% Catholic, 7% Jewish, and 17% were of other religions. SES: All were university students selected by convenience sampling at a university. Procedure: The study was cross‐sectional, and questionnaires were filled out in cafeteria, library and around the campus supervised by students as a part of a class project. Study 2 (Ukraine), Participants (*n* = 146): Age: 16–28 years old (*M*_age_ = 17.5, SD = 1.2). Gender: 72% females and 26% males. Ethnicity/culture: 98% Ukrainians, 2 were Russian, and 1 was Chinese; 80% were Orthodox Christian, 14% were Atheists, and 6% other. SES: All were university students selected through convenience sampling in a university. Procedure: Questionnaires were filled out in a cafeteria and hallways and supervised by students. The study was cross‐sectional.	Study 1 and 2: *Importance of family* self‐rated from 1 = not at all important to 7 = extremely important.	Study 1: *Radicalism Intention* Scale (RIS) from Activism‐Radicalism Intentions Scale (ARIS) answered on a 7‐point Likert. RIS included six items on radicalism understood as readiness to engage in illegal and violent political action (e.g., “I would continue to support an organization that fights for my group's political and legal rights even if the organization sometimes breaks the law”) and its Cronbach's *α* was 0.83. Questions responded as self‐reports thinking about an organization that the participants felt closest to. *Study 2: Radicalism Intention* Scale (RIS) from Activism‐Radicalism Intentions Scale (ARIS) focused on *radicalism* understood as “readiness to engage in illegal and violent political action,” answered on a 7‐point Likert, with a total number of 8 items. Items focused on self‐reported radicalism in relation to “my country” and “my political party.”
Nivette et al. (2017) Journal article Switzerland	Participants (*n* = 1,214): Age: 15–17 years old Gender: 50% males, 50% females. Ethnicity/culture: 48.3% with two parents born abroad, 27.9% with one parent born abroad, and 23.8% with two parents born in Switzerland. Mother's country of birth: 38.1% Switzerland, 16.2% former Yugoslavia, 6% Sri Lanka, 5.4%Portugal, 5.4% Germany, 4.3% Turkey, 8.1% other West‐European, 5.9% other Asian, 4.9% Latin America, 3.5% Africa, 2.3% other East‐European countries. Fathers' countries of birth “were similar.” Regarding religion, 25.1% were Roman Catholic, 21.6% Protestant, 20% Muslim, 18.9% had no religious affiliation, 7.6% Christian Orthodox, 5.2% Hindu, and 1.7% other. SES: Based on the ISEI scores *M* = 49.82, SD = 19.17 Procedure: Random sampling in schools. Initial sample – 1,675, study sample – 1,214 (attrition = 27.5%). The study was longitudinal, with risk and protective factors measured at wave 1 and radicalization measured two years afterwards. Data collected by “trained study staff” as a paper‐and‐pencil survey at schools.	*Parental involvement* defined as “the extent to which parents are involved in an adolescent's everyday life” measured through self‐reports. Items adapted from the Alabama Parenting Questionnaire (Shelton et al., 1996) and the Parenting Scale from the Criminological Research Institute of Lower Saxony (KFN). It included six items (e.g., “your parents show interest in what you do”) responded on a 5‐point response scale (*α* =0 .76).	*Violent extremist attitudes* defined as attitudes that “encourage, endorse, condone, justify, or support the commission of a violent criminal act to achieve political, ideological, religious, social, or economic goals” (International Association of Chiefs of Police, 2014) Four self‐reporting items that measured support for “using violence to fight against injustice; to defend the values, convictions, or religious beliefs of a group; to support groups that use violence; and to fight for a better world by using violence, committing attacks, or kidnapping people” responded on a 4‐point Likert scale (*α* = 0.80).
Robinson et al. (2017) Report Yemen	Participants (*n* = 1,200): Age: 18–64 years old Gender: 52.6% male and 47.4% female Ethnicity/culture: Participants from diverse ethnic‐cultural backgrounds, representative for the population of Yemen SES: 33.5% were illiterate or read and write, 30.1% with a primary diploma, 36.4% with a Bachelor's or higher Procedure: Sample randomly selected from the community. Surveys administered cross‐sectionally by local partners.	*Social ties with parents* were measured by asking about the extent to which parents had influence over participants' major life decisions. *Family assault* was measured by asking if family members have ever been assaulted by security forces or retaliated against by rival groups or their own groups in the past (one question each). All self‐reports.	*Opposition toward political violence* measured with two questions focused on support of foreign travel to fight occupier (e.g., “Do you support a friend traveling abroad to assist Muslims fighting a foreign occupier?”) answered on a 4‐point Likert scale. *Personal choice to not engage in political violence* measured with “a series of questions related to violent and nonviolent protests” including own willingness, friend's willingness and family willingness (all combined). All self‐reports.
Schbley (1988) PhD thesis Lebanon	Participants (*n* = 60): Age: 14–22 years old (*M* = 18.28, SD = 2.20) Ethnicity/culture: All Shi'a Muslim. Procedure: Participants recruited through convenience sampling selected among “guards manning roadblocks in the various factional territories in Bourj‐El‐Barajnet, a suburb of Beirut.” A researcher collected the data through interviews with gatekeepers of roadblocks, the study was cross‐sectional.	*Imprisonment of family members* measured with a question: “Are there any close members of your family in jail for” responded by choosing “Political reasons,”, “Religious Believes” and “Others.” *Marital status* measured with a question. Data collected through self‐reports.	*Willingness to commit terrorism* measured with a question: “Would you commit acts of terrorism, and/even take the life of innocent people to secure their release” where “their” refers to close family members in jail for the comparison related to having family members in prison. No details regarding the question used to compare married and unmarried participants. Data collected through self‐reports.
Siedler (2006) Report Germany	Study 1: Participants (*n* = 5,736) Age: *M*_age_ = 22.07, SD = 3.87 Gender: 50.6% females Ethnicity/culture: All with German citizenship. SES: Education: no degree – 20.3%, intermediate degree – 39.6%, high school degree – 24.7%, still in school – 15.4%. Mother's highest school degree: no degree or secondary – 46.5%, general school – 37.5%, high school degree – 16%. Father's highest school degree: no degree or secondary – 42.6%, general degree – 32.1%, high school degree – 25.4%. 8.2% reported parental unemployment during childhood Procedure: Data collected through survey. The study was longitudinal, with two waves, 5 years apart. Study 2Participants (*n* = 9200) Age: *M*_age_ = 22.36, SD = 4.29, aged 16–29 Age: *M*_age_ = 22.36, SD = 4.29, aged 16–29 Gender: 48% females Ethnicity/culture: All with German citizenship SES: Education: no degree – 16.2%, intermediate degree – 35.7%, high school degree – 42%, still in school – 6.1%. Mother's highest school degree: no degree or secondary – 46.5%, general school – 38.5%, high school degree – 15%. Father's highest school degree: no degree or secondary – 39.7%, general degree – 33.9%, high school degree = 26.4%. 5.3% reported parental unemployment during childhood. Procedure: Participants randomly selected and interviewed. The study was cross‐sectional.	*Parental unemployment during childhood*, no other details provided.	Study 1: *Participation in skinhead/neo‐Nazi groups* defined as having answered that they were a part of a skinhead group. Perception of “skinheads” measured on a “6‐point scale.” Variables measured through self‐reports. Study 2: *Participation in skinhead/neo‐Nazi groups* defined as either taking an active part in or occasionally attending a right‐wing extremist group measured with a question about attitudes towards “Neo‐Nazis and right‐wing skinheads” and other right‐wing groups (“Nationalistische Gruppierungen”).
van Bergen et al. (2016) Journal article The Netherlands	Participants (*n* = 133) Age: 14–18 years old (*M* = 15.58, SD = 0.95) Gender: 48% boys Ethnicity/culture: 97% were Turkish, 3% were Kurdish. Procedure: Participants selected through convenience sampling in schools (response rate of 97.8%). Data collected in schools (classrooms) through a survey website by the first author of the article with the teacher present during the data collection. The study was cross‐sectional.	Parental ethnic socialization measured with 19 of the 30 items (appropriate for Turkish–Dutch youth) of the self‐reporting ethnic socialization scale (Hughes et al., 2008) answered on a 5‐point scale. An exploratory factor analysis was conducted and found three factors including *cultural socialization* (five items focused on teachings about own culture, e.g., “How often have your parents taken you to places with predominantly people of your ethnic group [e.g., restaurants, language classes]?,” *α* = 0.77), *egalitarianism* (four items focused on stressing that we are all equal, e.g., “How often have your parents said that it is important to appreciate people of diverse ethnic backgrounds?”, *α* = 0.69) and *bias/mistrust* (10 items focused on emphasizing inequality and discrimination and being on guard against the other, e.g., “How often have your parents talked to you about how to handle situations where you are treated unfairly because of your ethnic background?,” *α* = 0.84).	*Attitude toward violent in‐group defense by others* (attitude toward others who use violence in defense of their ethnicity or religion) measured with one item (Doosje et al., 2013): “I can understand people who use violence to defend their ethnic and religious group” answered on a 5‐point Likert scale. *Willingness to use violence in defense of the in‐group* measured with one item (Doosje et al., 2013): “I would use violence to defend my ethnic origin or religion.” answered on a 3‐point scale. All self‐reports.
Victoroff et al. (2010) Journal article Palestinian Autonomous Territory of Gaza	Participants (*n* = 52): Age: 14 years old Gender: 100% boys Ethnicity/culture: Muslim Palestinians living in the al Shati refugee camp outside Gaza City Procedure: Data collected in schools in a refugee camp in groups of one to four, supervised by a Palestinian child psychologist in two randomly selected classes (response rate of 98.11%). The study was cross‐sectional.	*Family members wounded/killed by Israelis*, no other details provided.	*Support for religiously conditioned political aggression* measured through two self‐reporting items: “Religious ends justify any means,” and “Harming civilians is a justifiable tool in a Muslim arsenal.” With a correlation of *r* = 0.34, *p* = 0.015.
Vukčević Marković et al. (2021) Journal article Serbia	Participants (*n* = 271): Age: *M*_age_ = 16.30, SD = 0.69, ranging from 15 to 18 Gender: 72% females Procedure: Participants selected in schools. This was a cross‐sectional study with data collected in a school by a trained psychologist.	*Family dysfunction* measured with six items focused on parental neglect and parental maltreatment (e.g., “Physical and verbal conflicts happened often in my home while I was growing up”), a self‐reporting subscale from Bad socialization scale (Kneževic, 2003; Međedović, 2019) answered on a 5‐point Likert scale. Cronbach's *α* = 0.68.	The *proviolence* subscale from the revised Militant Extremist Mindset scale (Stankov et al., 2010, 2018), with 10 items focused on readiness to use violence to solve social problems (e.g., “Armed struggle is the only way that youths can redeem themselves and their society”) measured with self‐reports. Cronbach's *α* = 0.80.
Wildan and Qibtiyah (2020) Journal article Indonesia	Participants (*n* = 802): Age: 48% in Grade 10, 40% in Grade 11, and 12% in Grade 12 Gender: 36.9% females and 63.1% males Ethnicity/culture: 97% Muslim and 3% other. SES: 40% reported having basic needs mostly fulfilled, 57% reported “few fulfilment,” and 2% not fulfilled. Participants selected in schools by convenience sampling. Data collected in schools in a cross‐sectional study.	Parenting style including self‐reported *permissive parenting* (“parent's attempts to behave in a nonpunitive, acceptant, and affirmative manner toward the child's impulses, desires, and actions”), *authoritarian parenting* (“parent's attempt to shape, control, and evaluate the behavior and attitudes of children in accordance with a set standard, theologically motivated and formulated by a higher authority”), and *authoritative/democratic parenting* (“parents attempts to direct the children's activities in a rational, issues‐oriented manner”). No other details specified.	*Attitude toward violent and extremism ideology* based on Bassam Tibi's concept of Islamism measured through Noorhaidi Hasan's index of Islamism combined with Bassam Tibi's concept of Islamism including extremism (“a belief that political concept should be implemented revolutionarily even through violent actions”) and terrorism (“violent actions in the name of religious values which are conducted systematically”). Each concept measured with self‐reporting “4–6 questions on Islam and politics.”

Among the included documents, 24 were published as journal articles, five as research reports, four as theses and there were two publicly available datasets. Datasets were included and bivariate analyses were conducted including all the available family variables and types of radicalization.

Included studies were highly diverse in terms of geographic location including: Germany (*k* = 9), USA (*k* = 3), Palestinian territories (*k* = 2), Switzerland (*k* = 2), UK (*k* = 2), Australia (*k* = 1), Indonesia (*k* = 1), Iraq (*k* = 1), Italy (*k* = 1), Lebanon (*k* = 1), the Netherlands (*k* = 1), Pakistan (*k* = 1), Serbia (*k* = 1), Ukraine (*k* = 1), and Yemen (*k* = 1). Several other studies were cross‐national including UK, France, Germany and Spain (*k* = 1), UK, Germany and France (*k* = 1), USA and Canada (*k* = 1), USA, UK, EU, and Australia (*k* = 1), and 47 European countries (*k* = 1). Thus, 25 studies were conducted in Western countries and seven studies were conducted in non‐Western countries.

**Figure 2:**
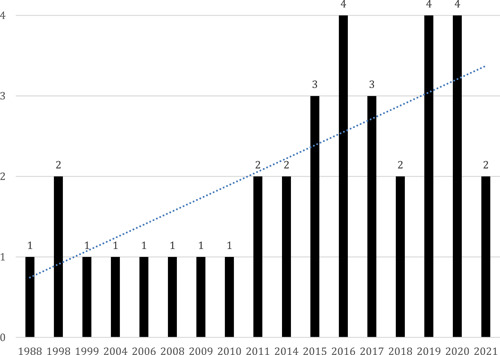
Number of studies per included year

The oldest included document was published in 1988, and the newest included document was published in 2021. Numbers of included documents per year are shown in Figure 2. As can be seen, the number of documents on family‐related risk and protective factors and radicalization increased throughout the history of the field. Only four documents were published before the 21st century, five documents were published in the 2000s and 25 documents were published in the 2010s (among which 22 were published from 2015 to 2021).

Included studies varied greatly in terms of the number of participants. The smallest study included 52 participants (Victoroff et al., 2010), and the largest study included 66,281 participants (European Values Study, 2008). The total number of participants in all the included studies was 148,081, although not all the study participants were included in each analysis because there were some missing data and some analyses only used subsets of participants. When reported, effect sizes included in this systematic review were calculated based on the actual number of participants per analysis, not the total number of participants per project.

Regarding age groups, 22 studies included adults (18 years old and above). One study with participants who were 17–33 years old—*M* = 19.6, one study with participants who were 16–28 years old—*M* = 17.5—were classified as adults given that they were university students and most of them were expected to be over 18 years old (Siedler, 2006). Also, one study with participants aged 14–22 was classified as including adults because the mean age was 18.5 and they were guards manning roadblocks who were mostly considered to be adults (Schbley, 1988). One study with 16–60 years old participants was classified as including adults because most of the participants were above 18 (Groppi, 2018). Eleven studies included adolescents (under 18 years old). Regarding sex, most of the studies included an approximately equal proportion of males and females, although some did not report participants’ sex and two studies (Dhumad et al., 2020; Victoroff et al., 2010) included males only.

Participants of the included studies differed on their ethnic‐cultural background. Some studies included representative samples of populations (e.g., European Values Study, 2008), other studies included university students (e.g., Moskalenko & McCauley, 2009), and several studies included immigrants of Muslim origin in Western countries (e.g., Abdi, 2019; Altunbas & Thornton, 2011). Muslim majority participants were studied in 15 included studies (assuming that participants from Muslim countries were Muslim even if their religion was not explicitly reported), and participants of other 17 studies were mostly non‐Muslim (religious groups other than Muslim were not explicitly studied). Participants had diverse socioeconomic statuses which, in most of the studies, were similar to general population of each geographic area.

There were only three longitudinal studies (Boehnke, Hagan, et al., 1998; Nivette et al., 2017; Siedler, 2006), and all the other studies were cross‐sectional. Participants of 18 studies were randomly selected from the population, ten studies used convenience sampling strategies, and four studies did not report the sampling strategy.

There was a great variety of family‐related risk and protective factors which relation to radicalization was measured in the included studies. Many of the studies included multiple family‐related risk factors. Descriptions of each family‐related factor measured in relation to radicalization in each included study are provided in Table 4. As shown in the summary Table 3, most of the family‐related variables were measured with either single‐item measures or several items designed *ad hoc*. Summary rating scales with calculated reliability coefficients were used only in nine out of the 33 included studies.

**Table 4:** Cambridge quality checklist results of the included studies

Study	Sampling	Response rates	Sample size	Measure of radicalization	Measure of family factor	Total correlate score	Methodology for risk/protective factors	Causality	Overall quality
Acevedo and Chaudhary (2015)	High	High	High	Low	High	High	High	Low	Higher
Abdi (2019)	High	Unclear	Low	Unclear	Unclear	Low	Low	Low	Lower
Altunbas and Thornton (2011)	High	Unclear	High	High	High	High	High	Low	Higher
Baier et al. (2016)	High	Low	High	Unclear	Unclear	Low	Low	Low	Lower
Berger (2016)	High	Unclear	High	Low	High	High	Low/High^a^	Low	Lower/higher^a^
Bhui, Everitt, et al. (2014), Bhui, Warfa, et al. (2014), Bhui et al. (2016)	High	Unclear	High	High	High	High	High	Low	Higher
Boehnke (2017)	Low	Unclear	Low	Low	Low	Low	Low	Low	Lower
Boehnke, Hagan, et al. (1998), Boehnke, Hefler, et al. (1998)	High	Low	High	Low	Low	Low	High	Low	Lower
Cherney and Murphy (2019)	High	Low	High	Low	High	High	High	Low	Higher
Clemmow (2020), Clemmow et al. (2020)	Low	Unclear	High	Unclear	Unclear	Low	Low/high*	Low	Lower
Cragin et al. (2015)	High	High	High	Unclear	Unclear	High	Low	Low	Lower
Delia Deckard and Jacobson (2015)	High	Low	High	Low	High	High	High	Low	Higher
Dhumad et al. (2020)	Low	Low	Low	High	Unclear	Low	Medium/high^a^	Low	Lower
European Values Study (2008)	High	Unclear	High	Low	Low	Low	Low/high^a^	Low	Lower
Fair and Hamza (2018)	High	High	High	Low	High	high	High	Low	Higher
Goede et al. (2020)	Low	Low	High	High	Low	Low	Low/high^a^	Low	Lower
Groppi (2017)	Low	Unclear	High	Low	Low	Low	High	Low	Lower
Hagan et al. (1999)	High	Low	High	Low	Low	Low	Low	Low	lower
Jahnke et al. (2021)	Unclear	Unclear	High	High	Low	Low	Low	Low	Lower
Kuhn (2004)	Unclear	Unclear	High	High	Low	Low	Low	Low	Lower
Manzoni et al. (2019)	High	Low	High	Unclear	Low	Low	Low/high^a^	Low	Lower
McCauley (2011)	High	High	High	Unclear	High	High	High	Low	Higher
Moskalenko and McCauley (2009), Study 1	Low	Unclear	Low	Unclear	Low	Low	Low	Low	Lower
Moskalenko and McCauley (2009), Study 2	Low	Unclear	Low	Unclear	Low	Low	Low	Low	Lower
Nivette et al. (2017)	High	High	High	High	High	High	High	Low	Higher
Robinson et al. (2017)	High	Unclear	High	Unclear	Low	Low	Low	low	Lower
Schbley (1988)	High	Unclear	Low	Low	High	Low	High	Low	Lower
Siedler (2006) Study 1	Unclear	Unclear	high	Unclear	High	Low	Medium	Low	Lower
Siedler (2006) Study 2	High	Unclear	High	Unclear	High	High	Medium	Low	Lower
Van Bergen et al. (2016)	Low	High	Low	Low	Low	Low	Low	Low	Lower
Victoroff et al. (2010)	High	High	Low	Low	High	High	High	Low	Higher
Vukčević Marković et al. (2021)	Unclear	Unclear	Low	High	Low	Low	Low	Low	Lwer
Wildan and Qibtiyah (2020)	Low	Unclear	Low	Unclear	Unclear	Low	Low	Low	Lower

a Higher quality rates were assigned for fixed factors such as marital status, family size and critical family events and were used in the meta‐analyses of these factors whereas the meta‐analyses of other factors included in the study were performed with rates assigned as lower quality.

Different terms and measurement instruments were used for radicalization. Among the included studies, 26 measured support for or expressed willingness to use violence to defend a cause (cognitive radicalization) whereas four measured an actual radical violent behavior. One study mixed cognitive and behavioral radicalization and two studies measured being a part of neo‐Nazi groups without specifying if this referred to cognition/attitudes or behavior. Islamist radicalization was measured in eleven studies, right‐wing extremism was measured in eight studies, left‐wing extremism was measured in two studies,^1^ and several other studies measured unspecified (violence used to defend a cause without clearly specifying the cause; for example, claiming that there may be certain circumstances where terrorism is justified in the European Values Study, 2008) radicalization. Most of the studies measured radicalization with a single‐item question, or several items designed *ad hoc*, with only ten studies based on summary rating scales with calculated reliability coefficients.

##### 6.1.2.1 Description of excluded studies

The number of located studies was high, but most of them were off‐topic and 86,307 were excluded after the title and abstract screening. A conservative approach to excluding studies was taken, and in case of doubts, documents were saved for a full text screening. At the full text screening stage, 117 documents were excluded because they did not provide empirical data. Most of these documents were reviews and qualitative studies. For example, Koehler (2013) described a family counseling intervention program against radicalization, but its evaluation was qualitative and could not be included.

Also, 71 studies were excluded because they did not measure the relation between a family‐factor and radicalization, and they did not include the results of an intervention. Some of these studies did not include any family‐related factors, while other studies did not include results on the relation between these factors and radicalization. For example, Aldegheiry (2019) reported means in items focused on different topics including family role in radicalization but did not perform bivariate analyses.

Twenty‐seven studies were excluded because they did not include any comparator to establish association between family factors and radicalization. Some studies were descriptive and only included prevalence of family issues among a sample of terrorists (e.g., Botha, 2014). Other studies compared radicalized individuals to other types of delinquents. For example, LaFree et al. (2018) compared violent and non‐violent radicals, but they did not include a general population. Altier et al. (2021) compared recidivist terrorists with non‐recidivists while Pyrooz et al. (2018) compared extremists with gang members regarding family factors.

Twenty documents were excluded because they did not meet the definition of radicalization required in this systematic review. Among them, radicalization was defined, for example, in terms of feeling Spanish, Catalan or both (Rico & Jennings, 2016), self‐identifying as socialists (Krout & Stagner, 1939), opposition to the established order in capitalist societies (Førland et al., 2010), voting for radical or populist parties (e.g., Coffé & Voorpostel, 2010) or mixing radicalization with other constructs that did not explicitly imply violence such as xenophobia (e.g., Baier et al., 2010; Klein‐Allermann et al., 1995) and women's rights (El‐Badayneh & ElHasan, 2017). Studies that mixed radicalization with other not explicitly violent behaviors or attitudes were excluded because high scores could be driven by responses to items that did not focus on the use of violence to defend a cause. Thus, these studies did not focus on support or commission of violence to defend a cause, including support for radical groups or terrorism and were therefore excluded.

Four documents were excluded because they reported results from a duplicated data set (Egger & Magni‐Berton, 2021; Kim, 2017; Littler, 2017; Narraina, 2013) without providing any new information. They were all based on a publicly available data set and documentation from the European Values Study (2008). This data set was included as such, together with its documentation, and bivariate analyses including all family‐related factors and radicalization were run.

##### 6.1.2.2 Quality assessment of included studies

The Cambridge Quality Checklist (Murray et al., 2009) was applied to the included studies and detailed results are shown in Table 4. As can be seen in Table 4, Nivette et al. (2017) was the only study with high quality on all the criteria except for causality. Twenty‐one studies scored high on sampling because they used a random sampling procedure. Seven studies reported response rates above 70%, and 24 studies included more than 400 participants. Eight studies were based on radicalization measures with high reliability, and 13 studies used a highly reliable measure of family factors or measured only demographic factors (e.g., marital status, number of children) which were not expected to have a calculated reliability coefficient. Regarding methodological quality for studying risk and protective factors, 17 studies were cross‐sectional (the lowest quality), three studies were retrospective (medium quality), and 13 studies were coded as prospective longitudinal either because they were truly longitudinal or because they measured fixed factors that necessarily preceded the measurement of radicalization (e.g., marital status, number of children). All the included studies scored two out of seven on the “causal risk/protective factors” criterion because none of them controlled for variables measured before the risk factor was measured.

As stated in the methods section, an overall quality was rated based on the correlation criterion (sampling, response rates, sample size, measure of radicalization and measure of family‐factors) and methodology for risk/protective factors criterions. Following Jolliffe et al. (2012) correlation criterion was rated as low if a study received low in one or two criteria and high if a study received high in three or more criteria. Thus, studies that received a high score on the correlation criterion and high in the risk/protective factors criterion were scored as higher quality (otherwise, studies were scored as lower quality). As can be seen in Table 4, nine studies were rated as having an overall higher quality and 24 studies were rated as having an overall lower quality.

#### 6.1.3 Synthesis of results

Included studies focused on various family factors associated with radicalization. Variables studied in each project were carefully coded and grouped into 14 meaningful categories. These factors are described in Table 5, together with variables included in each factor, definitions of each variable, studies where each variable was reported, and computed effect sizes (Fisher's *z*, SE and 95% CI). Although we believe that variables grouped in each factor focus on similar constructs and, therefore, were grouped in a meaningful way, Table 5 also provides a computed effect size per study and per variable if it was measured in more than one study, making it possible to read the results in a more conservative way. In total, 89 primary effect sizes were obtained, focused on 48 variables grouped into the 14 factors. The following sections focus on a meta‐analysis of each factor.

**Table 5:** Family‐related factors, variables included in each factor, definitions, citations to the primary studies and computed effect sizes

Family factor	Variables included	Definitions	Studies where the factor is reported	*Z*, SE (95%CI)
Critical family events	Family disintegrated	Reported “family disintegration”	Dhumad et al. (2020)	−0.011, 0.169 (−0.342, 0.321)
	Single parent family/parental divorce	Growing up in a single parent family and/or parental divorce	European Values Study (2008)	0.092, 0.015 (0.062, 0.123)
	Divorce of own children	Reported divorce of own children	European Values Study (2008)	−0.155, 0.023 (−0.161, −0.069)
	Divorce of family members other than parents and children	Reported divorce of other family member	European Values Study (2008)	0.040, 0.011 (0.018, 0.063)
	Family death	A family member died	Bhui et al. (2016)	−0.555, 0.338 (−1.217, 0.106)
			Clemmow (2020)	−0.197, 0.043 (−0.282, −0.112)
			European Values Study (2008)	−0.090, 0.016 (−0.121, −0.058)
			Total	−0.148, 0.056 (−0.258, −0.04)
	Family member killed	A family member has been killed	Dhumad et al. (2020)	0.174, 0.106 (−0.033, 0.382)
			Victoroff et al. (2010)	0.321, 0.143 (0.041, 0.601)
			Total	0.226, 0.085 (0.059, 0.393)
	Family member detained/imprisoned	Family arrested or detained by the outgroup security forces	Cragin et al. (2015)	Inconsistent, no data for the calculation of ES
			Robinson et al. (2017)	Inconsistent, no data for the calculation of ES
			Schbley (1988)	0.234, 0.146 (−0.052, 0.520)
	Critical family life events	Separation, divorce, serious illness or death of parents.	Goede et al. (2020)	0.025, 0.0134 (−0.001, 0.051)
			Manzoni et al. (2019)	0.003, 0.029 (−0.053, 0.060)
			Total	0.0211, 0.012 (−0.003, 0.045)
			TOTAL	−0.014, 0.034 (−0.081, 0.005)
Extremist family members	Parental right‐wing extremism		Boehnke (2017)	0.293, 0.083 (0.130, 0.457)
	Spouse involved in a wider movement		Clemmow (2020)	0.249, 0.056 (0.139, 0.360)
			TOTAL	0.263, 0.047 (0.172, 0.355)
Family commitment	Importance of family	Feeling that family is important	European Values Study (2008)	−0.048, 0.004 (−0.056, −0.040)
			Moskalenko and McCauley (2009), study 1	−0.070, 0.085 (−0.238, 0.097)
			Moskalenko and McCauley (2009), study 2	−0.050, 0.084 (−0.214, 0.114)
			Total	−0.05, 0.004 (−0.056, −0.040)
	Importance of parents	Feeling that parents are important	Cragin et al. (2015)	Inconsistent, no data for the calculation of ES
			Kuhn (2004)	0.050, 0.028 (−0.104, 0.004)
			Robinson et al. (2017)	Inconsistent, no data for the calculation of ES
	Family cohesion	Commitment and support from the family	Goede et al. (2020)	−0.040, 0.013 (−0.066,−0.014)
			Jahnke et al. (2021)	−0.001, 0.012 (−0.025, 0.024)
			Total	−0.020, 0.020 (−0.059, 0.019)
	Leisure time with parents	Leisure time spent in family with parents	Kuhn (2004)	−0.182, 0.028 (−0.236, −0.128)
	Parental involvement	Parental involved in adolescent's everyday life	Nivette et al. (2017)	0.182. 0.029 (0.26, 0.238)
	Parental care	Being praised by parents	Baier et al. (2016)	−0.097, 0.033 (−0.161, −0.033)
			Manzoni et al. (2019)	−0.074, 0.029 (−0.130, −0.017)
			Total	−0.060, 0.016 (−0.090, −0.030)
	Democratic/authoritative parents	Parents educate children in a rational, issues‐oriented manner	Wildan and Qibtiyah (2020)	0.055, 0.035 (−0.015, 0.124)
			TOTAL	−0.064, 0.019 (−0.101, −0.027)
Family conflict	Conflict with parents	Disagreements with parents about different topics	Kuhn (2004)	0.161, 0.028 (0.107, 0.216)
	Family conflict	Arguments	Abdi (2019)	0.060, 0.060 (−0.058, 0.178)
			Goede et al. (2020)	0.085, 0.013 (0.059, 0.111)
			Total	0.084, 0.013 (0.058, 0.110)
	Parental inconsistency	Inconsistent education, not doing what was promised to be done	Manzoni et al. (2019)	0.127, 0.029 (0.071, 0.184)
			TOTAL	0.113, 0.022 (0.071, 0.155)
Family size	Number of children	Participants report number of children	Dhumad et al. (2020)	−0.052, 0.063 (−0.177, 0.072)
			European Values Study (2008)	−0.046, 0.004 (−0.054, −0.038)
			Groppi (2018)	Nonsignificant, no data for the ES calculation
			Total	−0.046, 0.004 (−0.054, −0.038)
	Having children	Participants report having children	Clemmow (2020)	−0.065, 0.029 (−0.121, −0.009)
	Family size	Family size reported by the participants	Dhumad et al. (2020)	0.148, 0.070 (0.011, 0.286)
			TOTAL	−0.046, 0.012 (−0.069, −0.023)
Family socioeconomic factors	Parental education	Level of education completed by parent/s	European Values Study (2008)	0.022, 0.004 (0.014, 0.030)
			Kuhn (2004)	0.000, 0.028 (−0.054, 0.054)
			Manzoni et al. (2019)	−0.087, 0.029 (−0.143, −0.031)
			Total	−0.018, 0.032 (−0.080, 0.045)
	Education level of spouse	Reported education level of spouse	European Values Study (2008)	0.018, 0.005 (0.008, 0.028)
	Unemployment of spouse^a^	Reported spousal unemployment	European Values Study (2008)	0.065, 0.015 (0.036, 0.093)
	Parental unemployment^a^	Reported parental unemployment	European Values Study (2008)	0.072, 0.016 (0.041, 0.103)
			Siedler (2006), study 1	0.024, 0.007 (0.011, 0.036)
			Siedler (2006), study 2	0.033, 0.005 (0.024, 0.042)
			Total	0.037, 0.009 (0.020, 0.054)
	Parents reading books	Parents liked reading books	European Values Study (2008)	0.022, 0.011 (−0.000, 0.044)
	Parental poverty^a^	Difficulties in replacing things or making ends meet	European Values Study (2008)	−0.009, 0.013 (−0.034, 0.016)
	Parental unemployment or poverty^a^	Unemployed or recipients of social assistance	Baier et al. (2016)	0.033, 0.033 (−0.031, 0.097)
	Parents followed the news	Parents liked to follow the news	European Values Study (2008)	0.014, 0.011 (−0.006, 0.035)
			TOTAL	−0.028, 0.008 (−0.045, −0.012)
Family violence	Parental violence/harsh treatment/abusive home	Violent acts perpetrated by parents towards children	Baier et al. (2016)	0.080, 0.033 (0.016, 0.144)
			Clemmow (2020)	−0.182, 0.057 (−0.294, −0.069)
			Dhumad et al. (2020)	−0.301, 0.090 (−0.478, −0.124)
			Jahnke et al. (2021)	0.134, 0.012 (0.110, 0.158)
			Manzoni et al. (2019)	0.109, 0.013 (0.084, 0.135)
			Vukčević Marković et al. (2021)	0.224, 0.061 (0.104, 0.343)
			Total	0.045, 0.036 (−0.026, 0.116)
	Violence between parents	Witnessing violence between parents	Manzoni et al. (2019)	0.094, 0.029 (0.038, 0.150)
	Perpetrator of domestic abuse in adulthood	Participants perpetrate domestic abuse	Clemmow (2020)	0.007, 0.063 (−0.116, 0.130)
			TOTAL	0.054, 0.041 (−0.026, 0.133)
Immigrant spouse	Spouse from another country		European Values Study (2008)	0.012, 0.023 (0.032, −0.056)
			TOTAL	0.012, 0.023 (0.032, −0.056)
Marital status	Married	Being married	Acevedo and Chaudhary (2015)^1^	−0.025, 0.035 (−0.093, 0.044)
			Altunbas and Thornton (2011)	−0.098, 0.030 (−0.156, −0.039)
			Cherney and Murphy (2019)	−0.040, 0.035 (−0.110, 0.029)
			Delia Deckard and Jacobson (2015)^1^	0.151, 0.036 (0.081, 0.220)
			European Values Study (2008)	−0.075, 0.010 (−0.094, −0.057)
			Fair and Hamza (2018)^1^	−0.015, 0.008 (−0.031, 0.001)
			McCauley (2011)	−0.080, 0.086 (−0.248, 0.089)
			TOTAL	−0.025, 0.024 (−0.071, 0.022)
			Total unadjusted	−0.075, 0.009 (−0.092, −0.058)
	Single^a^	Being single	Berger (2016)^b^	0.088, 0.059 (−0.027, 0.204)
			Bhui, Warfa, et al. (2014)	0.201, 0.148 (−0.089, 0.490)
			Clemmow (2020)	0.041, 0.024 (−0.005, 0.087)
			Dhumad et al. (2020)	−0.109, 0.074 (−0.254, 0.037)
			European Values Study (2008)	0.143, 0.010 (0.124, 0.163)
			McCauley (2011)	0.145, 0.086 (−0.025, 0.314)
			Schbley (1988)	0.097, 0.311 (−0.512, 0.705)
			Total	0.078, 0.037 (0.047, 0.151)
			Total unadjusted	0.075, 0.043 (0.009, 0.160)
	Divorced^a^	Being divorced	Bhui, Everitt, et al. (2014)	−0.176, 0.323 (−0.810, 0.458)
			European Values Study (2008)	−0.010, 0.019 (−0.047, 0.027)
			Fair and Hamza (2018)^1^	−0.002, 0.018 (−0.039, 0.035)
			McCauley (2011)	−0.080, 0.150 (−0.374, 0.214)
			Total	−0.006, 0.012 (−0.028, 0.017)
			Total unadjusted	−0.012, 0.019 (−0.048, 0.025)
	Widowed^a^	Being widowed	European Values Study (2008)	−0.127, 0.018 (−0.163, −0.091)
			Fair and Hamza (2018)^b^	−0.031, 0.018 (−0.066, 0.004)
			McCauley (2011)	−0.071, 0.021 (−0.472, 0.330)
			Total	−0.079, 0.045 (−0.167, 0.009)
			Total unadjusted	−0.127, 0.018 (−0.162, −0.091)
			TOTAL	−0.028, 0.019 (−0.065, 0.010)
			TOTAL UNADJUSTED	−0.062, 0.015 (−0.091, −0.033)
Parental control	Parental control	Not being allowed to perform activities according to child's will without asking for permission/parental knowledge	Boehnke, Hefler, et al. (1998)	−0.04, 0.041 (−0.121, 0.041)
			Goede et al. (2020)	−0.095, 0.013 (−0.122, −0.069)
			Hagan et al. (1999)^b^^,^ c	−0.03, 0.021 (−0.072, 0.012)
			Manzoni et al. (2019)	−0.125, 0.029 (−0.181, −0.068)
			Total	−0.075, 0.022 (−0.118, −0.032)
			Total unadjusted/unimputed	−0.095, 0.017 (−0.128, −0.062)
	Authoritarian parent/s	Parents overly control child´s behavior and shape it according to a set standard	Dhumad et al. (2020)	0.113, 0.091 (−0.066, 0.291)
			Wildan and Qibtiyah (2020)	0.067, 0.035 (−0.003, 0.136)
			Total	0.073, 0.033 (0.008, 0.137)
	Permissive parents^a^	Parents accept and are permissive towards child's impulses, desires, and actions	Wildan and Qibtiyah (2020)	0.028, 0.035 (−0.041, 0.097)
			TOTAL	−0.048, 0.025 (−0.097, 0.000)
			TOTAL UNADJUSTED/UNIMPUTED	−0.051, 0.030 (−0.110, 0.085)
Parental “elbow mentality”	Parental hierarchical self‐interest	Parental engagement in an "elbow mentality" (e.g., Machiavellianism, acceptance of social inequality, competitive orientation)	Boehnke (2017)	0.055, 0.083 (−0.108, 0.218)
			TOTAL	0.055, 0.083 (−0.108, 0.218)
Parental politics communication	Communication with parents about politics	Talking with parents about politics	Kuhn (2004)	0.00, 0.028 (−0.054, 0.054)
			European Values Study (2008)	0.133, 0.016 (0.102, 0.165)
			TOTAL	0.069, 0.067 (−0.062, 0.199)
Religious household	Religious household	Grew up in a religious household	Clemmow (2020)	−0.042,0.024 (−0.089, 0.006)
			TOTAL	−0.042,0.024 (−0.089, 0.006)
Parental ethnic socialization	Parental bias/mistrust	Being on guard against other ethnicities, emphasizing discrimination	Van Bergen et al. (2016)	0.350, 0.088 (0.178, 0.522)
	Parental cultural socialization	Teaching predominantly about own culture	Van Bergen et al. (2016)	0.279, 0.088 (0.108, 0.451)
	Parental egalitarian socialization^a^	Teaching that people are all equal	Van Bergen et al. (2016)	−0.167, 0.088 (−0.338, 0.005)
			TOTAL	0.265, 0.051 (0.166, 0.364)

^a^ Reversed in the family factor analyses.

^b^ Based on adjusted data.

c Imputed values.

Among the studies that did not provide enough data for the calculation of the effects, Cragin et al. (2015) reported only marginal effects for regression analyses, Robinson et al. (2017) did not specify the type of effect included in the regression analyses, and Groppi (2018) only reported the *p* values, without other coefficients.

Each meta‐analysis included moderator analyses. Moderators used in the meta‐analyses are described in Table 6. These included publication years, locations, Muslim versus non‐Muslim participants, age groups, types of radicalization, ideologies and quality scores.

**Table 6:** Moderators used in the meta‐analyses

Study	Publication year	Location	Participants	Age group	Type of radicalization	Ideology	Overall quality
Acevedo and Chaudhary (2015)	2015	Western	Muslim	Adults	Cognitive	Islamist	Higher
Abdi (2019)	2019	Western	Muslim	Adults	Cognitive	Unspecified	Lower
Altunbas and Thornton (2011)	2011	Western	Muslim	Adults	Behavioral	Islamist	Higher
Baier et al. (2016)	2016	Western	Other	Adolescents	Behavioral	Right‐wing, Left‐wing, Islamist	Lower
Berger (2016)	2016	Western	Muslim	Adults	Cognitive	Islamist	Lower/Higher^a^
Bhui, Everitt, et al. (2014), Bhui, Warfa, et al. (2014), Bhui et al. (2016)	2015	Western	Muslim	Adults	Cognitive	Unspecified	Higher
Boehnke (2017)	2017	Western	Other	Adults	Cognitive/behavioral	Right‐wing	Lower
Boehnke, Hagan, et al. (1998), Boehnke, Hefler, et al. (1998)	1998	Western	Other	Adolescents	Cognitive	Right‐wing	Lower
Cherney and Murphy (2019)	2019	Western	Muslim	Adults	Cognitive	Islamist	Higher
Clemmow (2020), Clemmow et al., (2020)	2020	Western	Other	Adults	Behavioral	Unspecified	Lower
Cragin et al. (2015)	2015	Non‐Western	Muslim	Adults	Cognitive	Unspecified	Lower
Delia Deckard and Jacobson (2015)	2015	Western	Muslim	Adults	Cognitive	Islamist	Higher
Dhumad et al. (2020)	2019	Non‐Western	Muslim	Adults	Behavioral	Unspecified	Lower
European Values Study (2008)	2008	Western	Other	Adults	Cognitive	Unspecified	Lower
Fair and Hamza (2018)	2018	Non‐Western	Muslim	Adults	Cognitive	Islamist	Higher
Goede et al. (2020)	2020	Western	Other	Adolescents	Cognitive	Right‐wing, Islamist	Lower
Groppi (2018)	2018	Western	Muslim	Adults	Cognitive	Islamist	Lower
Hagan et al. (1999)	1999	Western	Other	Adolescents	Cognitive	Right‐wing	Lower
Jahnke et al. (2021)	2021	Western	Other	Adolescents	Cognitive	Unspecified	Lower
Kuhn (2004)	2004	Western	Other	Adults	Cognitive	Unspecified	Lower
Manzoni et al. (2019)	2019	Western	Other	Adolescents	Cognitive	Right‐wing, Left‐wing, Islamist	Lower
McCauley (2011)	2011	Western	Other	Adults	Cognitive	Unspecified	Higher
Moskalenko and McCauley (2009), Study 1	2009	Western	Other	Adults	Cognitive	Unspecified	Lower
Moskalenko and McCauley (2009), Study 2	2009	Western	Other	Adults	Cognitive	Unspecified	Lower
Nivette et al. (2017)	2017	Western	Other	Adolescents	Cognitive	Unspecified	Higher
Robinson et al. (2017)	2017	Non‐Western	Muslim	Adults	Cognitive	Unspecified	Lower
Schbley (1988)	1988	Non‐Western	Muslim	Adults	Cognitive	Unspecified	Lower
Siedler (2006), Study 1	2006	Western	Other	Adults	Unspecified	Right‐wing	Lower
Siedler (2006), Study 2	2006	Western	Other	Adults	Unspecified	Right‐wing	Lower
Van Bergen et al. (2016)	2016	Western	Muslim	Adolescents	Cognitive	Unspecified	Lower
Victoroff et al. (2010)	2010	Non‐Western	Muslim	Adolescents	Cognitive	Islamist	Higher
Vukčević Marković et al. (2021)	2021	Western	Other	Adolescents	Cognitive	Unspecified	Lower
Wildan and Qibtiyah (2020)	2020	Non‐Western	Muslim	Adolescents	Cognitive	Islamist	Lower

a Higher rates were assigned for fixed factors such as marital status, family size and critical family events.

##### 6.1.3.1 Critical family events

**Figure 3:**
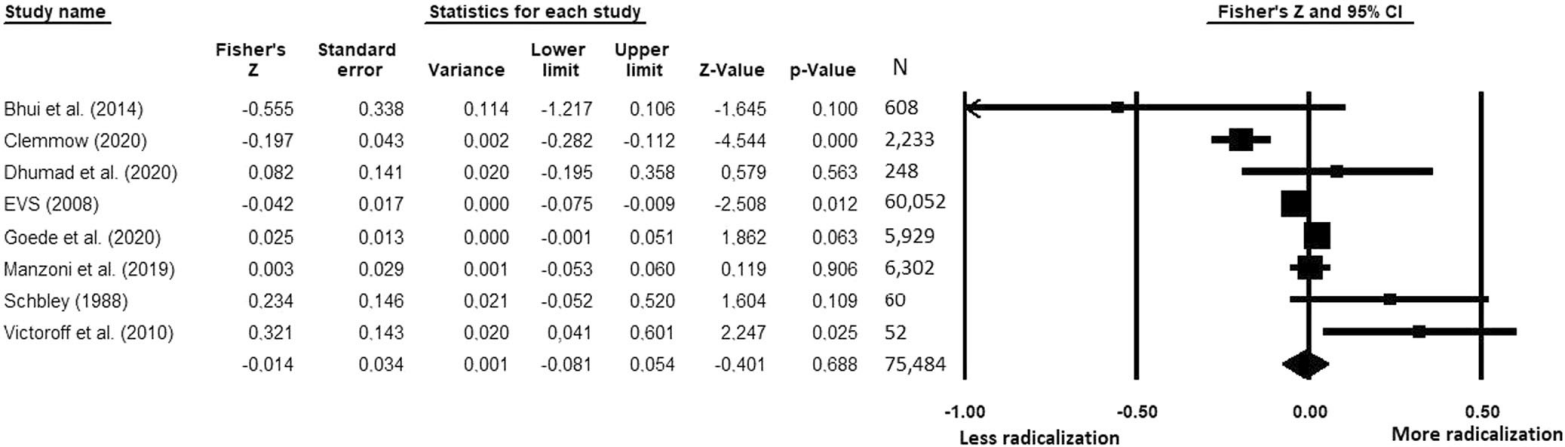
A forest‐plot with the meta‐analysis of the relation between critical family events and radicalization

Critical family events referred to disintegrated family (Dhumad et al., 2020), having grown up in a single parent family, divorce of own children and other family members (European Values Study, 2008), death of a family member (Bhui, et al., 2016; Clemmow, 2020; European Values Study, 2008), family member killed (Dhumad et al., 2020; Victoroff et al., 2010), family member detained or arrested (Schbley, 1988) and a critical family event such as separation, divorce, serious illness or death (Goede et al., 2020; Manzoni et al., 2019). Figure 3 shows a forest plot with the results of a meta‐analysis of the studies focused on critical family events. The overall effect size was nonsignificant (*z* = −0.014, 95% CI = −0.081, 0.054, *p* = 0.688), and studies were heterogeneous (*Q* = 40.42, *df* = 7, *p* < 0.001, *I* ^2^ = 82.68). Duval and Tweedie´s trim and fill trimmed one study to left of mean with an adjusted effect of *z* = −0.030 (95% CI = −0.100, 0.040). The effect did not become significant after removing any studies.

A moderator analysis is shown in Table 7. Studies were divided into older and newer based on median. The study location was a significant moderator, with studies located in non‐Western countries that showed a significant relation between having experienced critical family events and higher radicalization (*z* = 0.211, 95% CI = 0.049, 0.373). This significant effect is based on three studies. Among them, in Iraq, Dhumad et al. (2020) reported a nonsignificant relation between “family disintegration” and radicalization and a nonsignificant relation between family members having been murdered and radicalization. In Palestinian territories, Victoroff et al. (2010) found that having family members wounded or killed by Israelis was related to more radicalization. In Lebanon, Schbley (1988) reported a nonsignificant relation between having family members imprisoned for political or religious reasons and willingness to commit terrorism.

**Table 7:** A Moderator analysis of studies on critical family events

	*K (N)*	*z*	95% CI	*Q* between	*p*
Publication year					
Newer	5 (15,320)	−0.047	−0.147, 0.052	1.746	0.186
Older	3 (60,164)	0.141	−0.120, 0.402		
Location					
Non‐Western	3 (360)	0.211	0.049, 0.373	8.366	0.004
Western	4 (74,516)	−0.049	−0.116, 0.019		
Participants					
Muslim	4 (968)	0.131	−0.111, 0.373	1.840	0.175
Other	4 (76,516)	−0.043	−0.110, 0.024		
Age group					
Adolescents	3 (12,283)	0.028	−0.030, 0.086	1.224	0.269
Adults	5 (63,201)	−0.055	−0.190, 0.080		
Type of radicalization					
Behavioral	2 (2,481)	−0.090	−0.356, 0.176	0.546	0.460
Cognitive	6 (73,003)	0.013	−0.045, 0.070		
Ideology^a^					
Islamist	3 (6,484)	0.037	−0.070, 0.144	23.835	<0.001
Left‐wing	1 (6,302)	0.080	0.055, 0.105		
Right‐wing	2 (8,471)	−0.003	−0.032, 0.026		
Unspecified/other	5 (63,201)	−0.039	−0.106, 0.028		
Quality					
Higher	2 (660)	−0.064	−0.916, 0.789	0.008	0.930
Lower	6 (74,824)	−0.026	−0.090, 0.038		

a Given that some studies provided separate effect sizes for various ideologies, all the selected outcomes were used assuming independence. This is a conservative approach that artificially increases the *p* value.

The number of studies to compare extremist ideologies was low and does not allow for a meaningful comparison. Based on one study and one effect (Manzoni et al., 2019), having experienced a critical family event was related to more left‐wing radicalization.

##### 6.1.3.2 Extremist family

**Figure 4:**
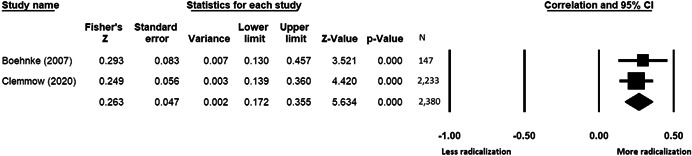
A Forest‐plot with the meta‐analysis of the relation between having extremist family members and radicalization

Only two studies focused on the relation between having extremist family members and own radicalization. These studies included parental right‐wing extremism (Boehnke, 2017), and spouse involved in a wider movement (Clemmow, 2020). Boehnke (2017) focused on right‐wing cognitive and behavioral radicalization with Western adults while Clemmow (2020) focused on lone‐wolf terrorism in Western countries. Both showed a significant relation between family members‘ radicalization and own radicalization, with a significant overall effect size (*z* = 0.263, 95%CI 0.172, 0.355). A forest plot with the included studies is shown in Figure 4. Given that only two studies were included, no moderator analysis or publication bias analysis was run.

##### 6.1.3.3 Family commitment

**Figure 5:**
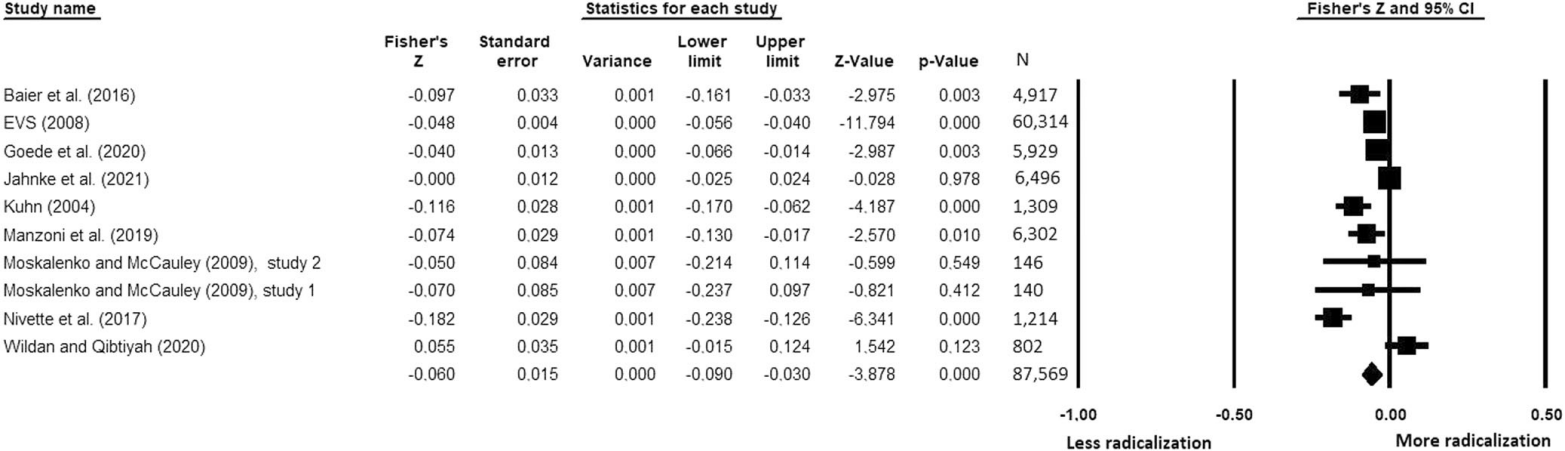
A forest‐plot with the meta‐analysis of the relation between family commitment and radicalization

Overall, high family commitment was related to less radicalization (*z* = −0.060, 95% CI = −0.090, −0.030). As shown in Figure 5, 10 studies reported on variables related to family commitment. These studies were heterogeneous (*Q* = 54.34, *df* = 9, *p* < 0.001, *I* ^2^ = 83.44). Variables included in this factor were importance of family (European Values Study, 2008; Moskalenko & McCauley, 2009; studies 1 and 2), feeling that parents are important (Kuhn, 2004), family cohesion (Goede et al., 2020; Jahnke et al., 2021), leisure time with parents (Kuhn, 2004), parental involvement (Nivette et al., 2017), parental care (Baier et al., 2016; Manzoni et al., 2019), and democratic parenting (Wildan & Qibtiyah, 2020). Duval and Tweedie´s trim and fill did not trim any studies, and the effect was not reduced to nonsignificant after removing any of the included studies. No study removal brought the effect to nonsignificant.

The number of studies was too small for a meta‐regression analysis, and thus, separate moderator analyses were run (see Table 8). Moderator analyses showed that the relation between high family commitment and low radicalization was only true in Western (non‐Muslim) countries, although there was only one non‐Western (Muslim) country comparison study and results need to be interpreted with caution. Regarding ideology, family commitment was related to lower right‐wing, left‐wing and unspecified/other radicalization, but not to Islamist radicalization. Quality could not be used as a moderator because all the studies were rated as lower quality.

**Table 8:** A Moderator analysis for family commitment

	*k (N)*	*Z*	95%CI	*Q* between	*p*
Publication year					
Newer	6 (72,755)	−0.059	−0.080, −0.037	0.031	0.861
Older	4 (14,814)	−0.051	−0.141, 0.040		
Location/Participants					
Non‐Western/Muslim	1 (802)	0.055	−0.015, 0.124	10.451	0.001
Western/Other	9 (86, 767)	−0.070	−0.100, −0.040		
Age group					
Adolescents	6 (25,660)	−0.056	−0.108, −0.003	0.186	0.666
Adults	4 (61,909)	−0.071	−0.116, −0.025		
Type of radicalization					
Behavioral	1 (4,917)	−0.097	−0.161, −0.033	1.253	0.263
Cognitive	9 (82,652)	−0.056	−0.088, −0.024		
Ideology^a^					
Islamist	4 (7,610)	−0.005	−0.058, 0.047	15.361	0.002
Left‐wing	2 (9,627)	−0.118	−0.148, −0.088		
Right‐wing	3 (13,388)	−0.062	−0.090, −0.034		
Unspecified/other	6 (69,619)	−0.070	−0.129, −0.011		

a Given that several studies provided separate effect sizes for various ideologies, all the selected outcomes were used assuming independence. This is a conservative approach that artificially increases the *p* value.

##### 6.1.3.4 Family conflict

**Figure 6:**
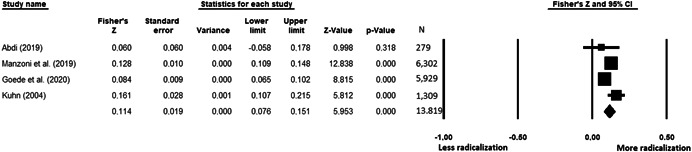
A forest‐plot with the meta‐analysis of the relation between family conflict and radicalization

Only four studies reported results on the relation between family conflict and radicalization. Among them, Kuhn (2004) focused on conflict with parents, Abdi (2019) and Goede et al. (2020) on family conflict in general, and Manzoni et al. (2019) on parental inconsistency. A forest plot with the effect sizes of these studies and an overall effect size is shown in Figure 6. Family conflict was related to more radicalization (*z* = 0.113, 95% CI = 0.071, 0.155). There was no evidence of heterogeneity (*Q* = 7.400, *df* = 3, *p* = 0.060, *I* ^2^ = 59.459). Duval and Tweedie's trim and fill publication bias trimmed one study to right of mean, showing the adjusted values of *z* = 0.119 (95% CI = 0.078, 0.160). No study removal brought the effect size to nonsignificant.

A moderator analysis is shown in Table 9. Given that only four studies were available, the moderator analysis did not provide very meaningful results. The ideology was a significant moderator, with a weaker relation between family conflict, right‐wing and left‐wing radicalization. One older study (Kuhn, 2004) had a bigger effect size than the newer studies. All the studies focused on family conflict and radicalization were conducted in Western countries, measured cognitive radicalization and were rated as lower quality.

**Table 9:** Moderator analysis for family conflict

	*k (N)*	*z*	95%CI	*Q* between	*p*
Publication year					
Newer	3 (12,510)	0.092	0.067, 0.117	5.142	0.023
Older	1 (1,309)	0.161	0.107, 0.215		
Participants					
Muslim	1 (279)	0.060	−0.058, 0.178	0.841	0.359
Other	3 (13,540)	0.120	0.072, 0.168		
Age group					
Adolescents	2 (12,231)	0.099	0.060, 0.137	0.248	0.618
Adults	2 (1,588)	0.125	0.030, 0.220		
Ideology^a^					
Islamist	2 (6,432)	0.095	0.009, 0.182	8.238	0.041
Left‐wing	1 (6,302)	0.151	0.126, 0.176		
Right‐wing	2 (8471)	0.097	0.068, 0.126		
Unspecified/other	2 (1588)	0.125	0.030, 0.220		

a Given that several studies provided separate effect sizes for various ideologies, all the selected outcomes were used assuming independence. This is a conservative approach that artificially increases the *p* value.

##### 6.1.3.5 Family size

**Figure 7:**
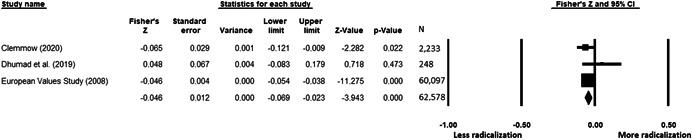
A forest‐plot with the meta‐analysis of the relation between family size and radicalization

Three studies measured the relation between family size and radicalization. Among them, Dhumad et al. (2020) focused on the number of children and family size, European Values Study (2008) on the number of children and Clemmow (2020) on having children. An overall effect size (see Figure 7) shows that a bigger family size was related to less radicalization (*z* = −0.046, 95% CI = −0.054, −0.038). There was no evidence of heterogeneity (*Q* = 2.419, *df* = 2, *p* = 0.298, *I* ^2^ = 17.314). Duval and Tweedie's trim and fill publication bias analysis did not trim any studies but removing the European Values Study (2008) from the analysis brought the effect size to nonsignificant.

A moderator analysis did not find any significant moderators (see Table 10). All participants were adults, and all ideologies were unspecified. Quality could not be used in the moderator analysis because all the studies were rated as lower quality.

**Table 10:** A Moderator analysis for family size

	*k (N)*	*Z*	95%CI	*Q* between	*p*
Publication year					
Newer	2 (2,481)	−0.025	−0.131, 0.081	0.153	0.695
Older	1 (60,091)	−0.046	−0.054, −0.038		
Location/Participants					
Non‐Western/Muslim	1 (248)	0.048	−0.083, 0.179	1.981	0.159
Western/Other	2 (62,330)	−0.046	−0.054, −0.038		
Type of radicalization					
Behavioral	2 (2481)	−0.025	−0.131, 0.081	0.153	0.695
Cognitive	1 (60,097)	−0.046	−0.054, −0.038		

##### 6.1.3.6 Family socioeconomic factors

**Figure 8:**
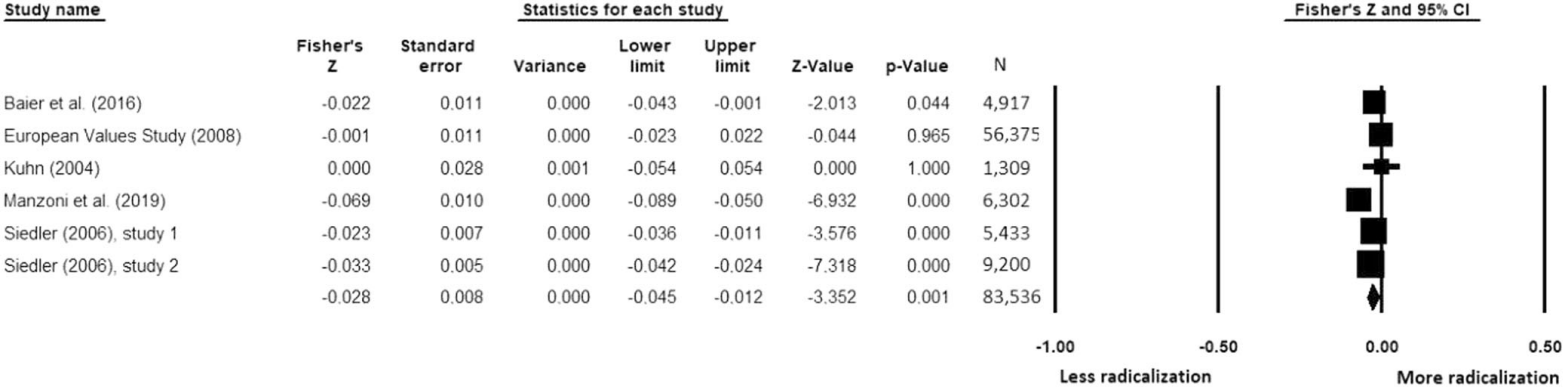
A forest‐plot with the meta‐analysis of the relation between family socioeconomic factors and radicalization

Family socioeconomic factors analysis included variables such as parental education (European Values Study, 2008; Kuhn, 2004; Manzoni et al., 2019), education level and unemployment of the spouse (European Values Study, 2008), parental unemployment (European Values Study, 2008; Siedler, 2006 study 1 and 2), parents liked to read books (European Values Study, 2008), parental poverty (European Values Study, 2008), parental unemployment or reception of social assistance (Baier et al., 2016) and parents liked to follow the news (European Values Study, 2008). An overall effect size (see Figure 8) showed that high family socioeconomic status was related to less radicalization (*z* = −0.028, 95% CI = −0.045, −0.012) with heterogeneous effect sizes (*Q* = 25.220, *df* = 5, *p* < 0.001, *I* ^2^ = 80.174). Duval and Tweedie's trim and fill publication bias analysis trimmed one study to left of mean with an adjusted effect size of *z* = −0.030 (95% CI = −0.045, −0.014). A removal of any study did not bring the effect to nonsignificant.

A moderator analysis is shown in Table 11. The only significant moderator was ideology, where high socioeconomic family status was found to be related to less Islamist, left‐wing and right‐wing ideology, but not to unspecified ideology (e.g., supporting terrorism in general, without specifying anything else). All the studies were rated as lower quality, so quality could not be used as a moderator.

**Table 11:** Moderator analysis for family socioeconomic status

	*k (N)*	*z*	95% CI	*Q* between	*p*
Publication year/age group					
Newer/adolescents	2 (11,219)	−0.046	−0.092, 0.001	0.999	0.318
Older/adults	4 (72,317)	−0.021	−0.035, −0.006		
Type of radicalization					
Behavioral	1 (4,917)	−0.022	−0.043, −0.001	0.445	0.800
Cognitive	3 (63,986)	−0.025	−0.079, 0.028		
Unspecified	2 (14,633)	−0.030	−0.039, −0.020		
Ideology^a^					
Islamist	2 (879)	−0.089	−0.155, −0.023	19.063	<0.001
Left‐wing	2 (9,627)	−0.037	−0.066, −0.008		
Right‐wing	4 (22,846)	−0.038	−0.053, −0.023		
Unspecified/other	2 (57,684)	0.002	−0.013, 0.018		

a Given that several studies provided separate effect sizes for various ideologies, all the selected outcomes were used assuming independence. This is a conservative approach that artificially increases the *p* value.

##### 6.1.3.7 Family violence

**Figure 9:**
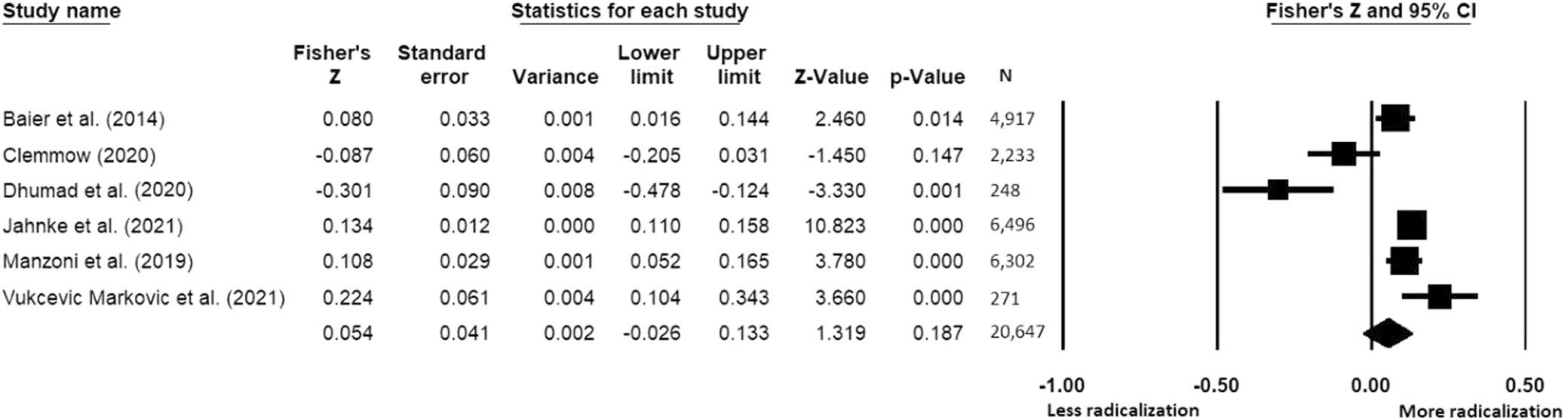
A forest‐plot with the meta‐analysis of the relation between family violence and radicalization.

Family violence included variables such as parental violence and harsh treatment (Baier et al., 2016; Clemmow, 2020; Dhumad et al., 2020; Jahnke et al., 2021; Vukcevic Markovic et al., 2021), violence between parents (Manzoni et al., 2019) and being a perpetrator of domestic abuse (Clemmow, 2020). A meta‐analysis of these studies (see Figure 9) showed that the relation between family violence and radicalization was nonsignificant (*z* = 0.052, 95% CI = −0.032, 0.135), with heterogeneous studies (*Q* = 39.217, *df* = 5, *p* < 0.001, *I* ^2^ = 87.250). The effect became significant (*z* = 0.098, 95% CI = 0.039, 0.158) after the removal of Dhumad et al. (2020). Duval and Tweedie's trim and fill analysis did not trim any studies.

There were several significant moderators of the relation between family violence and radicalization (see Table 12). Only one study on the relation between family violence and radicalization in non‐Western Muslim countries was included (Dhumad et al., 2020). Although the effect based on Western samples showed that family violence was related to more radicalization, Dhumad et al. (2020) who studied convicted terrorists in Iraq found that harsh treatment by parents was more common among controls than among terrorists. Family violence was related to more radicalization in adolescents, but this relation was not significant in adults. Also, family violence was related to more cognitive radicalization, but there was no evidence of this relation for behavioral radicalization. Regarding ideology, family violence was related to Islamist, right‐wing and left‐wing radicalization, but there was no evidence of this relation for unspecified radicalization. Quality could not be used as a moderator because all the studies were rated as lower quality.

**Table 12:** A Moderator analysis of studies on family violence

	*k (N)*	*z*	95% CI	*Q* between	*p*
Publication year					
Newer	3 (9,000)	0.093	−0.047, 0.234	0.905	0.341
Older	3 (11,521)	−0.004	−0.149, 0.140		
Location/participants					
Non‐Western/Muslim	1 (248)	−0.301	−0.478, −0.124	17.521	<0.001
Western/Other	5 (20,219)	0.098	0.039, 0.158		
Age group					
Adolescents	4 (17,986)	0.124	0.087, 0.160	8.096	0.004
Adults	2 (2,481)	−0.183	−0.392, 0.025		
Type of radicalization					
Behavioral	3 (7,398)	−0.089	−0.291, 0.114	4.521	0.033
Cognitive	3 (13,069)	0.134	0.098, 0.170		
Ideology^a^					
Islamist	2 (879)	0.097	0.052, 0.142	14.435	0.002
Left‐wing	2 (9,627)	0.138	0.104, 0.171		
Right‐wing	2 (8,159)	0.068	0.050, 0.087		
Unspecified/other	4 (9,248)	0.023	−0.078, 0.124		

a Given that one study provided separate effect sizes for various ideologies, all the selected outcomes were used assuming independence. This is a conservative approach that artificially increases the *p* value.

##### 6.1.3.8 Immigrant spouse

There was only one study focused on having an immigrant spouse (European Values Study, 2008), and its relation to radicalization was nonsignificant (*z* = 0.012, 95% CI = 0.032, −0.056).

##### 6.1.3.9 Marital status

Seven studies reported results on the relation between radicalization and being married (Acevedo & Chaudhary, 2015; Altunbas & Thornton, 2011; Cherney & Murphy, 2019; Delia Deckhard & Jacobson, 2015; European Values Study, 2008; Fair & Hamza, 2018; McCauley, 2011). Also, seven studies measured the relation between being single and radicalization (Berger, 2016; Bhui, Warfa, et al., 2014; Clemmow, 2020; Dhumad et al., 2020; European Values Study, 2008; McCauley, 2011; Schbley, 1988). Four studies measured the relation between being divorced and radicalization (Bhui, Warfa, et al., 2014; European Values Study, 2008; Fair & Hamza, 2018; McCauley, 2011), and three studies focused on the relation between being widowed and radicalization (European Values Study, 2008; Fair & Hamza, 2018; McCauley, 2011).

**Figure 10:**
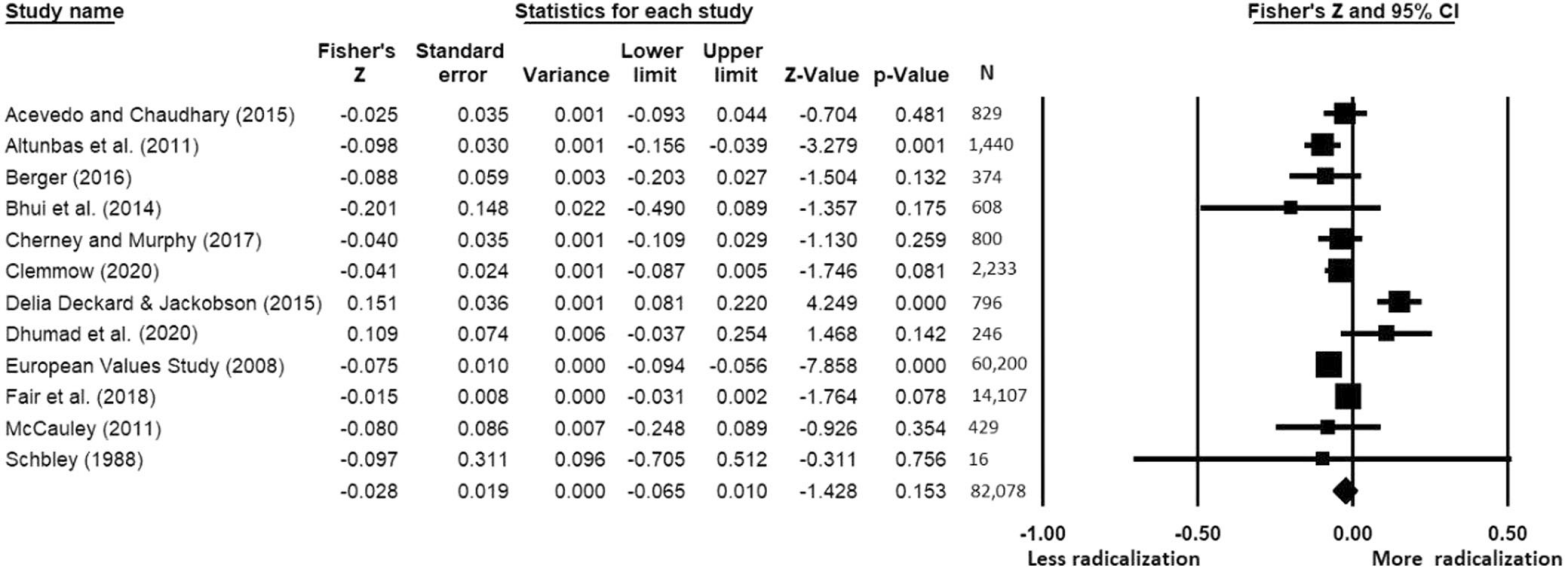
A forest‐plot with the meta‐analysis of the relation between being married and radicalization

An overall effect size was calculated for being married (see Figure 10). This was done by entering only the effect for being married for the studies that measured different marital statuses (e.g., European Values Study, 2008), and reversed coding the effects of being single for the studies that did not measure being married (e.g., Bhui, Warfa, et al., 2014). An overall effect size for marital status was nonsignificant (*z* = −0.028, 95% CI = −0.065, 0.010). Included effects were heterogeneous (*Q* = 61.28, *df* = 11, *p* < 0.001, *I* ^2^ = 82.050).

Duval and Tweedie's trim and fill publication bias analysis trimmed three studies to right of mean, with an adjusted effect of *z* = −0.015 (95% CI = −0.052, 0.022). Removal of Delia Deckhard and Jacobson (2015) produced a significant effect between being married and lower radicalization. A sensitivity analysis that included only unadjusted studies found that being married was related to lower radicalization (*z* = −0.062, 95% CI = −0.091, −0.033).

A moderator analysis for the relation between marital status and radicalization is shown in Table 13. There were no significant moderators. All participants were adults, and therefore, no moderator analysis for age groups was performed.

**Table 13:** Moderator analysis for marital status

	*k (N)*	*z*	95%CI	Q between	*p*
Publication year					
Newer	5 (17,760)	−0.024	−0.053, 0.005	0.050	0.823
Older	7 (64,318)	−0.034	−0.112, 0.045		
Location					
Non‐western	3 (14,369)	0.011	−0.070, 0.092	1.046	0.306
Western	9 (67,790)	−0.038	−0.088, 0.011		
Participants					
Muslim	9 (19,216)	−0.013	−0.067, 0.042	4,009	0.045
Other	3 (62,862)	−0.070	−0.088, −0.053		
Type of radicalization					
Behavioral	3 (3919)	−0.036	−0.114, 0.043	0.048	0.827
Cognitive	9 (78,159)	−0.023	−0.070, 0.023		
Ideology					
Islamist	6 (18,346)	−0.017	−0.074, 0.041	0.955	0.329
Unspecified/other	6 (63,732)	−0.053	−0.096, −0.009		
Quality					
Higher	8 (19,383)	−0.026	−0.080, 0.028	0.192	0.661
Lower	4 (62,695)	−0.043	−0.096, 0.010		

##### 6.1.3.10 Parental control

**Figure 11:**
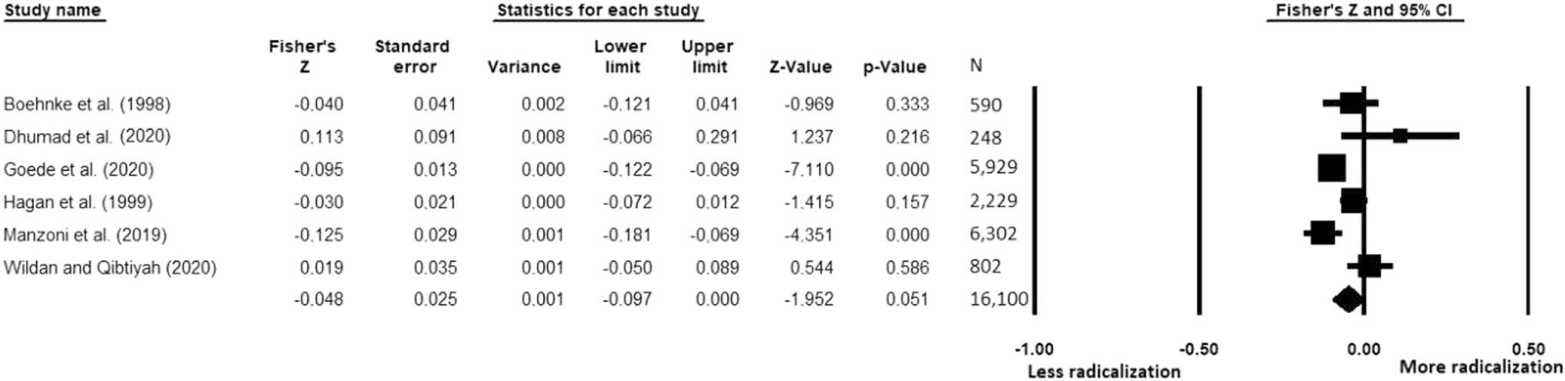
A forest‐plot with the meta‐analysis of the relation between parental control and radicalization

Parental control meta‐analysis included studies focused on parental control (Boehnke, Hefler, et al., 1998; Goede et al., 2020; Hagan et al., 1999; Manzoni et al., 2019), authoritarian parenting (Dhumad et al., 2020; Wildan & Qibtiyah, 2020) and reversed permissive parenting (Wildan & Qibtiyah, 2020). Overall, the relation between parental control and radicalization was nonsignificant (*z* = −0.048, 95% CI = −0.097, 0.0001; see Figure 11), and studies were heterogeneous (*Q* = 21.70, *df* = 5, *p* = 0.001, *I* ^2^ = 76.96). Duval and Tweedie's trim and fill analysis trimmed one study to left of mean with an adjusted effect of *z* = −0.058 (95% CI = −0.106, −0.009). Removal of Wildan and Qibtiyah (2020) and Dhumad et al. (2020) resulted in a significant relation between low parental control and more radicalization.

A moderator analysis for the relation between parental control and radicalization is shown in Table 14. Location was a significant moderator. Studies conducted in Western countries found a relation between low parental control and higher radicalization. Ideology was also a significant moderator. It was found that low parental control was related to higher right‐wing and left‐wing radicalization, and there was no evidence of the relation between parental control and Islamist or unspecified radicalization. All the studies were rated as lower quality and quality could not be used as a moderator.

**Table 14:** Moderator analysis for parental control and radicalization

	*k (N)*	*z*	95%CI	*Q* between	*p*
Publication year					
Newer	4 (13,304)	−0.051	−0.122, 0.021	0.202	0.653
Older	2 (2,819)	−0.032	−0.069, 0.005		
Location/participants					
Non‐western/Muslim	2 (1,050)	0.032	−0.033, 0.096	7.215	0.007
Western/Other	4 (15,050)	−0.075	−0.118, −0.032		
Age/Type of radicalization					
Adolescents/Cognitive	5 (15,852)	−0.058	−0.105, −0.012	3.304	0.069
Adults/Behavioral	1 (248)	0.113	−0.066, 0.291		
Ideology^a^					
Islamist	3 (7,234)	−0.010	−0.063, 0.043	76.87	<0.001
Left‐wing	1 (6,302)	−0.224	−0.249, −0.199		
Right‐wing	4 (11,290)	−0.074	−0.125, −0.025		
Unspecified	1 (248)	0.113	−0.066, 0.291		

a Given that one study provided separate effect sizes for various ideologies, all the selected outcomes were used assuming independence. This is a conservative approach that artificially increases the *p* value.

##### 6.1.3.11 Parental “elbow mentality”

Parental “elbow mentality” was only measured in one study (Boehnke, 2017) focused on cognitive and behavioral right‐wing radicalization in Western adults. “Elbow mentality” was defined as parental Machiavellianism, acceptance of social inequality and competitive orientation. The relation between parental “elbow mentality” and radicalization was nonsignificant (*z* = 0.055, 95% CI = −0.108, 0.218).

##### 6.1.3.12 Parental politics communication

**Figure 12:**
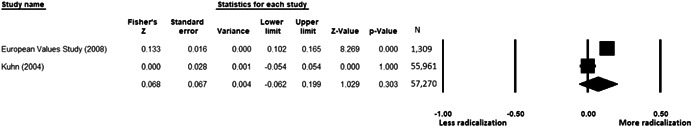
A forest‐plot with the meta‐analysis of the relation between parental politics communication and radicalization

Parental politics communication defined as talking about politics with parents was measured in two studies (European Values Study, 2008; Kuhn, 2004). Both studies focused on a cognitive unspecified radicalization in Western adults. The relation was nonsignificant (*z* = 0.069, 95% CI = −0.062, 0.199), as shown in Figure 12.

##### 6.1.3.13 Religious household

Only one study (Clemmow, 2020) focused on the relation between having grown up in a religious household and radicalization, with nonsignificant results (*z* = −0.042, 95% CI = −0.089, 0.006). Clemmow (2020) studied lone‐actor terrorists in Western countries, with no specific radicalization ideology.

##### 6.1.3.14 Parental ethnic socialization

Parental ethnic socialization was defined as bias and mistrust induced by parents towards other cultures, teaching predominantly about own culture, and teaching that people are all equal (reversed), and it was measured in one study (Van Bergen et al., 2016). Van Bergen e al. (2016) focused on Western adolescent Muslims and measured cognitive unspecified radicalization. It was found that parental ethnic socialization was related to more radicalization (*z* = 0.265, 95% CI = 0.166, 0.364).

##### 6.1.3.15 Comparison of the effect sizes among the family factors

**Figure 13:**
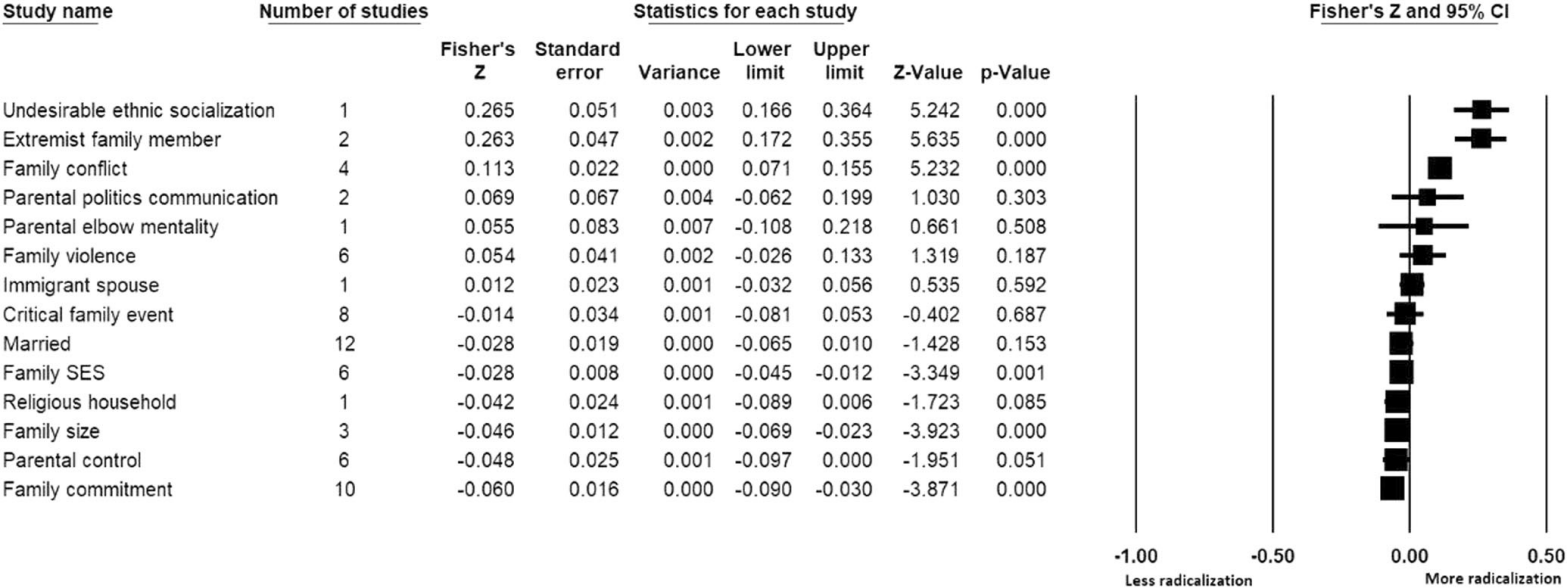
The relation between each family‐factor included in this systematic review and radicalization

Figure 13 shows the relation between each family‐factor included in this systematic review and radicalization. Parental ethnic socialization, extremist family members and family conflict were significant risk factors for radicalization. High family socioeconomic status, larger family size, and high family commitment were significant protective factors against radicalization.

**Figure 14:**
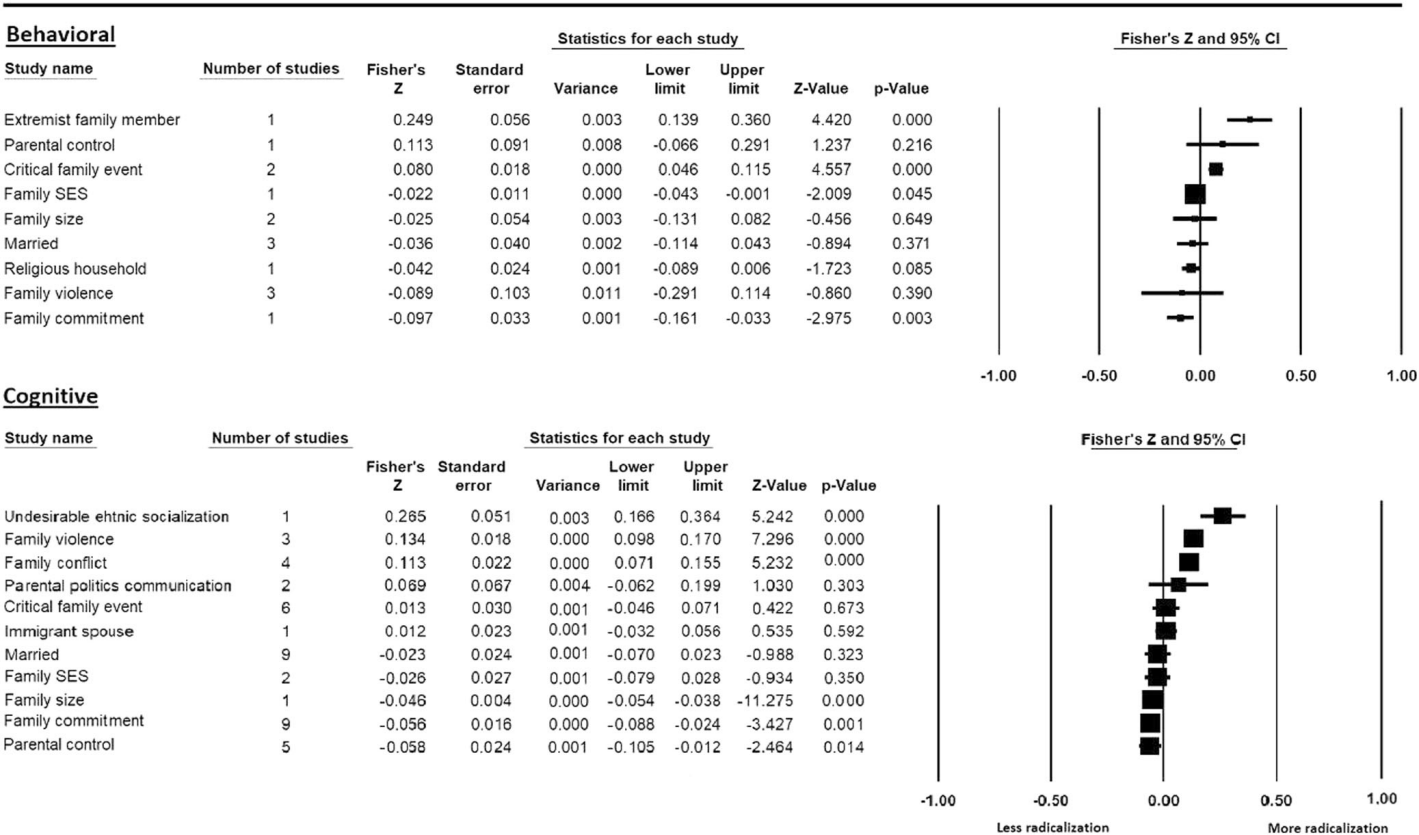
The relation between each family‐factor included in this systematic review and behavioral and cognitive radicalization

Figure 14 shows the results separately for behavioral and cognitive radicalization. Having extremist family members and critical family events were found to be risk factors for behavioral radicalization. High family SES and family commitment were protective against behavioral radicalization. Parental ethnic socialization, family violence and conflict were risk factors for cognitive radicalization, and bigger family size, family commitment, and parental control were protective.

**Figure 15:**
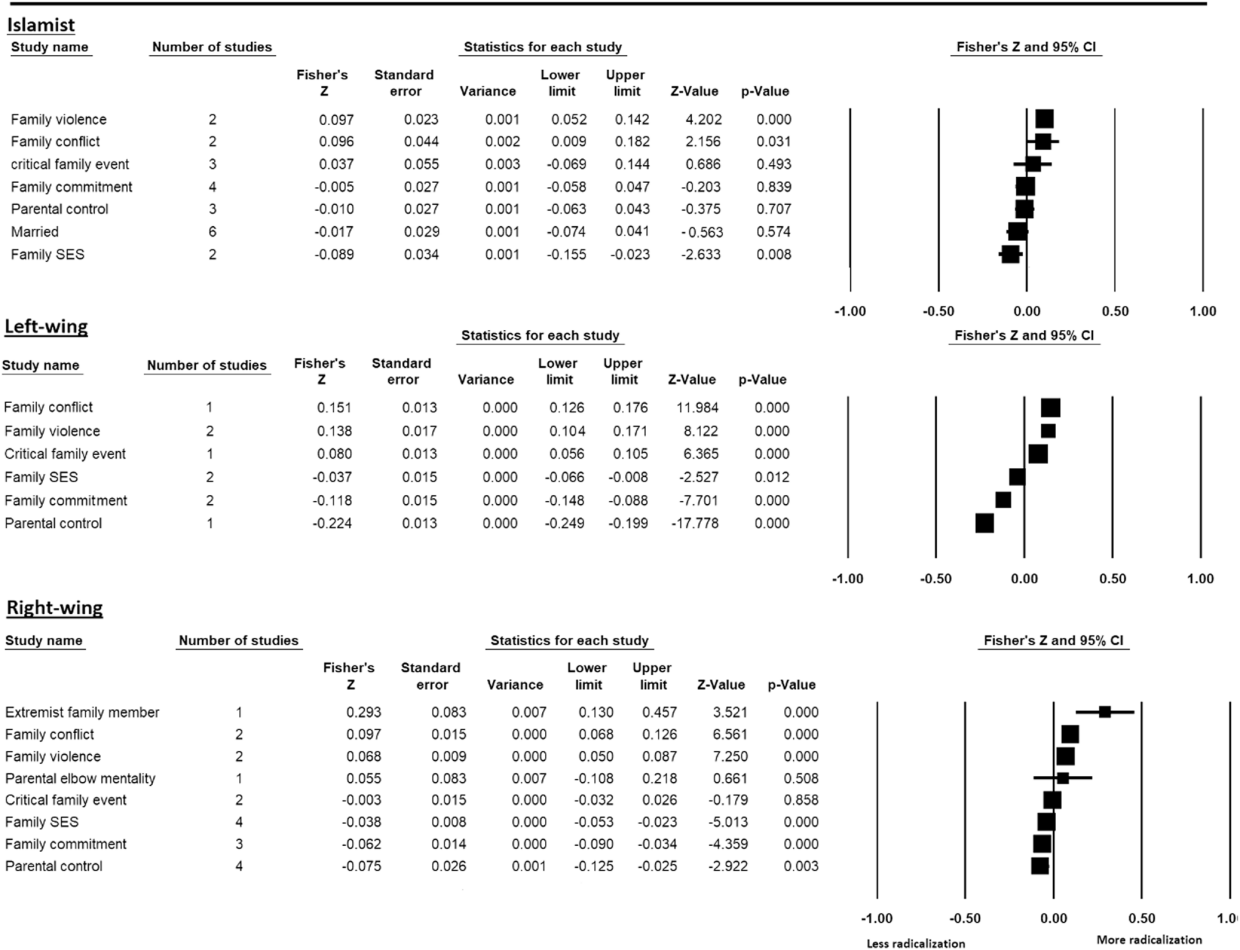
The relation between each family‐factor included in this systematic review and different radical ideologies

Figure 15 shows the relation between family factors and different ideologies, including Islamist, right‐wing and left‐wing radicalization. Family conflict and violence were risk factors for Islamist radicalization, whereas high family socioeconomic status was protective. Regarding left‐wing radicalization, family conflict, family violence and critical family events were risks, and high family SES, family commitment and parental control were protective. Having an extremist family member, family conflict and violence were risk factors for right‐wing radicalization, and high family SES, family commitment and parental control were protective.

### 6.2 Sensitivity analysis

A sensitivity analysis treating outcomes as uncorrelated and therefore independent was performed. As can be seen in Table 15, only minor variations with respect to the previously described results were observed. The relation between family‐size and radicalization was no longer significant when outcomes such as number of children and family size were treated as uncorrelated. Nevertheless, it is reasonable to suggest that, in most of the cases, these outcomes are highly correlated, and therefore, probably the analysis which treats them as such is more accurate. Another variation was found in the relation between family violence and radicalization that became significant if treated as independent. For family violence, combined outcomes included different ideologies (Islamist, right‐wing and left‐wing), and types of violence (e.g., violence between parents and punishment of children). These outcomes could indeed be relatively independent and, therefore, it is possible that family violence is indeed related to more radicalization.

**Table 15:** Sensitivity analysis treating all the outcomes as uncorrelated and independent

	Fisher's Z	SE	95% CI	
Critical family events	−0.012	0.025	−0.060	0.037
Extremist family	0.270	0.041	0.191	0.350
Family commitment	−0.061	0.014	−0.088	−0.034
Family conflict	0.114	0.019	0.076	0.151
Family size	−0.031	0.027	−0.084	0.021
Family SES	−0.022	0.007	−0.035	−0.008
Family violence	0.090	0.015	0.060	0.119
Marital status	−0.033	0.018	−0.067	0.002
Parental control	−0.046	0.027	−0.099	0.007

## 7 DISCUSSION

### 7.1 Summary of main results

This systematic review was conducted to summarize evidence on families and radicalization. Specifically, its first objective was to describe family‐related risk and protective factors for radicalization. The second objective was to analyze the impact of radicalization on families and the third objective was to discover if family‐based interventions were effective against radicalization. Although there are some previous systematic reviews focused on risk or protective factors for radicalization in general (Emmelkamp et al., 2020; Lösel et al., 2018; Wolfowicz et al., 2021) none of them focused specifically on families and radicalization. Thus, this is the first comprehensive systematic review conducted to describe the scientific field of quantitative research on families and radicalization.

This systematic review did not locate any studies on consequences of radicalization for families or family‐focused interventions that would meet its inclusion and exclusion criteria. Some qualitative studies on the impact of radicalization were located (e.g., Guru, 2012a, 2012b), but this systematic review included only quantitative studies. Also, some qualitative evaluations of family‐focused intervention programs were located (e.g., Koehler, 2013), but again, these were excluded according to our inclusion and exclusion criteria.

A systematic review of family‐related risk and protective factors for radicalization included a total number of 33 studies published in 35 documents. Among the included studies, 30 provided sufficient information for the calculation of effects, and given that many studies focused on the relation between various family‐related risk and protective factors and radicalization, the current systematic review included 89 effect sizes and 48 variables grouped into 14 family‐related factors.

The current systematic review showed that parental ethnic socialization, extremist family members and family conflict were significant risk factors for radicalization whereas high family socioeconomic status, bigger family size, and high family commitment were significant protective factors against radicalization. Thus, several family‐related risk and protective factors for radicalization have been described, although many were based on a limited number of studies and results should be taken with caution. Moreover, this systematic review was intended to focus on relations with different family members including parents, siblings, children, spouses, and extended family. However, it was not possible to study the relation between the exact nature of the family‐relation and radicalization because the number of included studies per family factor was not high enough. It would have been desirable to compare, for example, family‐related risk and protective factors for parent‐child relations to those that exist among siblings, grandparents and grandchildren, and extended family. Future systematic reviews focused on family‐related factors for radicalization should aim at analyzing these possible nuances.

Moderator analyses were conducted when more than two studies per factor were included. It was found that critical family events (such as death, divorce or imprisonment) were a risk factor for radicalization in non‐Western countries, and there was no evidence of their relation to radicalization in Western countries. Based on one study and one effect (Manzoni et al., 2019), having experienced a critical family event was related to more left‐wing radicalization. Family commitment was protective against radicalization in Western countries, and there was no evidence of its relation to radicalization in non‐Western Muslim countries, although only one study in a non‐Western location analyzed the relation between family commitment and radicalization. Based on two studies, the relation between family conflict and radicalization was weaker for the right‐wing ideology and Islamist, compared to left‐wing and unspecified (Goede et al., 2020; Manzoni et al., 2019). High family socioeconomic status was protective against Islamist, left‐wing and right‐wing radicalization, but there was no evidence of the relation between family socioeconomic status and unspecified radicalization.

Family violence was related to more radicalization in Western countries, but the only included study that measured this relation in non‐Western Muslim countries (Dhumad et al., 2020) found that harsh treatment by parents was more common among controls than among terrorists. Family violence was related to more radicalization in adolescents, but there was no evidence of this relation in adults. Also, family violence was related to more cognitive radicalization, but the relation between family violence and behavioral radicalization was not significant. Family violence was related to Islamist, right‐wing and left‐wing radicalization, but there was no evidence of this relation for unspecified radicalization.

There was no evidence of a significant relation between marital status and overall radicalization, although being married was related to less radicalization in non‐Muslim participants. Parental control was related to less radicalization in Western countries, but there was no evidence for this relation in Non‐western Muslim countries. Moreover, there was no evidence of the relation between parental control and Islamist radicalization whereas high parental control was related to less left‐wing radicalization (although only based on one study by Manzoni et al., 2019) and to right‐wing radicalization. Again, moderator analyses results should be taken with caution taking into account the low number of studies in many comparisons.

### 7.2 Overall completeness and applicability of evidence

This systematic review is based on extensive searches in 25 databases, including databases with high‐impact journals (e.g., Web of Science, Scopus) and databases that include gray literature (e.g., ProQuest Dissertations & Theses Global, Google Scholar). Hand searchers were performed in ten leading journals in the field. Searches were also performed on 12 websites of different agencies and professional organizations to locate gray literature. Authors of the included studies and other leading researchers in the field were contacted and asked to provide published and unpublished studies on family and radicalization. They were all provided with the protocol so they could get familiar with all the details of the current systematic review. Reference lists of the included studies and previously published systematic reviews on risk and protective factors for radicalization were screened. There were no restrictions regarding publication year, geographic location, types of participants and publication language. Published and unpublished studies were included, together with two publicly available datasets that were analyzed by the authors of this systematic reviews to provide the most comprehensive review possible. Thus, we believe that the current systematic review includes up‐to‐date evidence of what is known regarding families and radicalization worldwide.

Unfortunately, there were no studies focused on the impact of radicalization on families or family‐based interventions against radicalization. Given our extensive searches, we believe that such studies are yet to be conducted. Although there are some interesting qualitative studies on these topics (e.g., Koehler, 2013), these were not included in the current review. There are many initiatives that focus on working with families to decrease radicalization (Radicalisation Awareness Network, 2021), and it would be useful to quantitatively evaluate their effectiveness.

This review includes 14 family‐related risk and protective factors for radicalization. These factors were extracted from primary studies located in various geographic areas, including Western and non‐Western countries. There were different age groups studied in the primary studies, including adults and adolescents, and different ethnic‐cultural and religious backgrounds such as Muslim and other participants. Studies focused on cognitive and behavioral radicalization, including Islamist, right‐wing, left‐wing and unspecific ideologies. Thus, a broad range of factors in heterogeneous samples and ideologies was included. This systematic review can be therefore considered comprehensive as it shows a whole panorama of research in the field. Nevertheless, the included heterogeneous set of primary studies allowed to include only a limited number of effects per analysis, and very limited moderator analyses. It was not possible to run meta‐regressions because the number of included studies was too low. Meta‐regressions would have been especially important to discover which characteristics of the studies could be uniquely influencing the results. Unfortunately, regarding cultures/ethnicities, only Muslim versus other backgrounds were analyzed because these were the groups present in the primary studies. It would have been desirable to include other ethnic‐cultural and religious backgrounds, but they did not exist in the primary studies. These are important limitations of the current systematic review.

Only three included studies were longitudinal (Boehnke, Hagan, et al., 1998; Nivette et al., 2017; Siedler, 2006), and each longitudinal study focused on different family‐related factors. Thus, it was not meaningful to run a moderator analysis with cross‐sectional versus longitudinal studies. Family‐related factors were studied as risk and protective for radicalization on a theoretical basis, but it is not possible to establish causal relations or even the order of appearance of factors and radicalization in time in most of the studies. Thus, it is possible that some factors are consequences of radicalization instead of risk and protective factors. For example, family conflict was included in the current systematic review as a risk factor for radicalization, but it is also possible to think of situations in which radicalization could cause conflicts within families.

This systematic review included primary studies based on rigorous inclusion and exclusion criteria, providing evidence based on quantitative studies that defined radicalization as the use of or support for violence to defend a cause, including support for radical groups and terrorism. Projects that defined radicalization differently were not included to ensure a comparable set of studies. Nevertheless, some of these studies could be synthesized in separate systematic reviews, focused on, for example, voting for radical parties. Also, a meta‐synthesis of qualitative studies could be especially interesting for future projects.

This systematic review included 14 family‐related risk and protective factors for radicalization, but many other family factors could have a crucial role in radicalization and have not been studied yet. Although this systematic review is likely to have included most of the family‐related factors related to radicalization studied up to date, there are probably many other factors that will be studied in future and could contribute to the understanding of radicalization. There were three studies that did not provide enough information to calculate the effects and were therefore excluded from the meta‐analyses. Taking into account the publication bias analyses, it is unlikely that these studies affected greatly the results. Nevertheless, given a limited number of studies per meta‐analysis, results need to be interpreted with caution.

Thus, the current systematic review includes a complete and comprehensive set of studies focused on family‐based risk and protective factors for radicalization. Although with some limitations, the applicability of the results is high. Knowledge gathered in this systematic review could be used to design future family‐based interventions to decrease risks and increase protective factors for radicalization. Future family‐focused interventions could be evaluated and included in future systematic reviews.

### 7.3 Quality of the evidence

None of the located studies focused on family related consequences of radicalization or family‐focused interventions met the inclusion criteria of the current systematic review. Regarding family‐related risk and protective factors, 33 studies were included, among which, 30 provided enough information to be included in the meta‐analysis. Although the number of included studies was high enough to conduct a high‐quality systematic review and meta‐analysis, family‐related risk and protective factors were heterogeneous, and they were divided into fourteen meaningful categories. Four categories (parental ethnic socialization, parental “elbow mentality,” immigrant spouse, and having grown up in a religious household) included only one study each, so no meta‐analysis could be conducted. The remaining factor categories included at least two studies, and therefore, ten meta‐analyses were conducted. Nevertheless, the number of studies included in each meta‐analysis was relatively low, which is an important limitation regarding the quality of the included evidence, even though no evidence of publication bias was found in most of the meta‐analyses.

An application of the Cambridge Quality Checklist (Murray et al., 2009) to the included studies made it possible to evaluate the quality of the primary data used in this systematic review. The highest quality study was Nivette et al. (2017). All the included studies scored two out of seven on the “causal risk/protective factors” criterion because none of them controlled for variables measured before the risk factor was measured. This is a common limitation in criminological research in general as found in a review of studies on disrupted families and crime where Jolliffe et al. (2012) tested the Cambridge Quality Checklist. Jolliffe et al. (2012) included 60 studies and all of them scored two out of seven on the causality criterion. Thus, factors included in our review cannot be considered causal.

Even though causality or even time ordering cannot be assumed based on the quality of the studies included in this systematic review, other methodological quality criteria of many of the included studies were met. In most of the studies, samples were relatively large, and random selection of participants made it possible to collect the data from several representative samples. Nevertheless, high‐quality validated instruments were rarely used. Thus, there are some limitations regarding the quality of the evidence included in this systematic review, but this synthesis of family‐related risk and protective factors is a steppingstone to understand the role of families in radicalization.

### 7.4 Limitations and potential biases in the review

This systematic review was performed by two independent researchers in all its stages, including searches, screening of titles and abstracts, full text screening, and coding of the included studies. Agreement rates at all these stages were high. Thus, the current review adhered to high standards and the risk of potential biases is expected to be low. Nevertheless, there are two potential sources of bias that need to be acknowledged. One of them is the possibility of having missed some studies focused on one of the three objectives of this systematic review. The number of located studies was high, with 86,591 titles and abstracts screened. Although this was done by two independent researchers, some studies could have been missed among this high number of titles and abstracts. Moreover, searches and screening focused on family and radicalization, but some studies could include some family‐related factors without mentioning them in titles and abstracts. This is especially plausible for demographic factors that are frequently controlled for in the field, but not even mentioned in texts of the articles, such as marital status or having children. Some of these studies were indeed missed during our searches in databases, but hopefully all of them were retrieved after screening the references of the included studies and previous systematic reviews.

Another possible limitation of the current systematic review is the way in which categories of risk and protective factors were created. This systematic review included 48 variables, most of them measured in one study, or a very limited number of studies. Thus, it was necessary to classify these variables into meaningful categories. This was carefully done to only include studies focused on highly similar constructs in each category, and this is a common practice in the field (see, e.g., Emmelkamp et al., 2020; Wolfowicz et al., 2021). Nevertheless, some categories still included outcomes that were not identical (e.g., parental violence, violence between parents and perpetration of domestic abuse in adulthood all classified as family violence). If the number of included studies per variable was much higher, it would have been ideal to focus on each variable separately. It is also possible that other researchers would have grouped these variables in different categories.

Even with these limitations, risk of bias of this systematic review is rather low because of its high transparency. Variables included in each factor are described in detail, together with effects for each included study and overall effects for each variable. A definition of each variable is also provided. Thus, besides using our classification into family‐related factors, readers can easily check what studies are included in each factor and draw conclusions based on each variable separately.

### 7.5 Agreements and disagreements with other reviews

Findings of this systematic review are mostly in agreement with previous reviews, although the number of studies focused on families and radicalization included in this systematic review is much higher in comparison to the previous reviews. Thus, the number of family‐factors studied in this systematic review is also higher. At the same time, some studies focused on family and radicalization that were included in the previous reviews were excluded from the current review based on our inclusion and exclusion criteria. Categories used in the previous systematic reviews differed from our categories probably because they included a lower number of studies.

Based on five studies, Lösel et al. (2018) found that protective family‐related factors against radicalization were positive parenting, non‐violent family members and ownership of a house. In our systematic review, family commitment was found to be related to less radicalization, having extremist family members was related to more radicalization, there was no evidence of the relation between family violence and radicalization, and no studies on ownership of a house were included. Thus, our results are mostly in agreement with Lösel et al. (2018), although our systematic review includes many more studies focused on families and radicalization.

Emmelkamp et al. (2020) included only one domain focused on family and radicalization based on six studies, called negative parenting. They did not find evidence of a significant relation between negative parenting and radicalization, although the effect was in the risk direction. Our systematic review did not include such a category, but we did find that parental ethnic socialization was related to more radicalization, family conflict was also related to more radicalization, and there was no evidence of the relation between family violence and radicalization. Thus, our results regarding family and radicalization are more specific, but they do point out in a similar direction.

Wolfowicz et al. (2021) included several family‐related risk and protective factors for radicalization. They concluded that having children (based on two studies), being married (based on 11 studies), parental academics (based on four studies), parental involvement (based on 12 studies), parental control (based on four studies) were protective against radical attitudes, whereas family violence (based on five studies) and parental abuse (based on six studies) were risk factors. There was no evidence of the relation between marital status (based on six studies) and behavioral radicalization, whereas parental involvement (based on four studies) was related to less behavioral radicalization. Several studies focused on family and radicalization included in Wolfowicz et al. (2021) were excluded from the current meta‐analysis and several other studies not included in Wolfowicz et al. (2021) were included in the current systematic review. Wolfowicz et al. (2021) analyzed separately radical attitudes, intentions and behaviors (and no overall results were calculated), whereas the current systematic review differentiated behavioral and cognitive radicalization. If the results focused on cognitive and behavioral radicalization are compared, similarly to Wolfowicz et al. (2021), there was no evidence of the relation between marital status and behavioral radicalization, but parental involvement as such was not analyzed in relation to behavioral radicalization in our study. Regarding cognitive radicalization, the current systematic review found that parental ethnic socialization, family violence and conflict were risk factors, bigger family size, higher commitment and parental control were protective, and there was no evidence regarding the relation between other factors and radicalization. Thus, our results point out in a similar direction as Wolfowicz et al. (2021), although there are differences regarding the set of primary studies included.

## 8 AUTHORS' CONCLUSIONS

### 8.1 Implications for policy and practice

This systematic review has important implications for policy and practice. Although causal relations between family‐related risk and protective factors based on our results cannot be established, it is reasonable to suggest that policies and practice should aim at decreasing family‐related risks and increasing protective factors for radicalization. Although only based on one study (van Bergen et al., 2016), parental ethnic socialization was found to be related to more radicalization. Thus, decreasing bias against other cultures and ethnicities, teaching about different cultures and teaching that people are all equal would be desirable. Based on two studies (Boehnke, 2017; Clemmow, 2020), having extremist family members is related to more radicalization. It is therefore desirable to counter extremism in families, not only in individuals. A bigger family size was protective. It is possible that having a family, including children and second‐degree family members, is a sign of a better functioning in the society for some individuals, and this should be assessed. Family commitment should also be promoted as it was found to be protective against radicalization. High family socioeconomic status was also protective. Socioeconomic status could be related to structural economic inequality in societies. Thus, policy makers and practitioners should aim at increasing the level of education and social wellbeing to reduce exclusion of less affluent individuals and families.

Tailored interventions that would aim at decreasing specific family‐related risk factors and increasing specific‐family related protective factors described in this systematic review could be useful to decrease radicalization. Besides implications for future tailored interventions, findings of this systematic review have some implications for risk assessment. Given that having extremist family members was found to be related to own radicalization, risk of radicalization in family members of extremists should be evaluated.

### 8.2 Implications for research

This systematic review did not locate any studies focused on the impact of radicalization on families or family‐based interventions. Thus, studying the impact of radicalization on family is urgently needed. This is especially true given that some qualitative studies found that families of radicalized individuals are highly affected (Guru, 2012a, 2012b). Our systematic review included a wide range of family‐related risk and protective factors, showing that families may be important in the process of radicalization. Thus, family‐based interventions need to be urgently designed, implemented and evaluated. It is crucial to discover what works regarding family‐based interventions and conduct such programs in different geographic areas.

The included studies were mostly cross‐sectional, and it was impossible to establish which family‐related factors were causal or preceded radicalization. Thus, longitudinal research is urgently needed. Longitudinal studies, especially controlled nonexperimental studies with a measure of within‐individual change and randomized controlled trials (Murray et al., 2009) could clarify causal relations between family‐factors and radicalization. Given that longitudinal studies are especially difficult to conduct taking into account costs and time needed for these projects, it would be crucial for policy makers to provide funding that would make them possible. Also, more possible family‐related risk and protective factors should be studied in future. Our searches located many qualitative studies, but their synthesis was beyond the scope of the current systematic review. Thus, a meta‐synthesis of qualitative studies could also fill important gaps in knowledge and should be performed in future. The current systematic review shows that family‐factors are important for radicalization, its results can be useful for policy and practice against radicalization, and it opens up new research horizons.

## CONTRIBUTIONS OF AUTHORS

This systematic review will be conducted by a team from University of Cordoba (Spain).

- Content: Zych and Nasaescu
- Systematic review methods: Zych and Nasaescu
- Statistical analysis: Zych and Nasaescu
- Information retrieval: Zych and Nasaescu

Izabela Zych is an Associate Professor in Psychology at the University of Cordoba and has broad experience and expertise in conducting systematic reviews and meta‐analyses focused on different topics related to violence. She has successfully led and published similar studies, including papers in the leading journals in the field. She is a member of Campbell Collaboration Crime and Justice Group Steering Committee.

Elena Nasaescu is a PhD candidate supervised by Izabela Zych, who is currently in the final stage of her PhD. She has a specific training in conducting systematic reviews and meta‐analyses, and excellent methodological skills.

## DECLARATIONS OF INTEREST

The authors declare that there are no conflicts of interests. However, one of the review authors has an internal role within the Campbell Collaboration Crime and Justice Group. Specifically, Izabela Zych is a member of the Campbell Collaboration Crime and Justice Group Steering Committee, including the Campbell/DHS Counter‐Terrorism Systematic Review Advisory Board. Thus, Izabela Zych is not involved in any editorial or internal Campbell Collaboration communications about this review.

## PLANS FOR UPDATING THIS REVIEW

We plan to produce an updated version of this review every 4 years. The lead author (Izabela Zych) will be in charge of coordinating the revised versions.

## Supporting information

Supporting information.

Supporting information.

## Footnotes

1 Note that several studies measured different types of radicalization and are counted in more than once.
