# Supplementary material for: Is radicalization a family issue? A systematic review of family‐related risk and protective factors, consequences, and interventions against radicalization
Source: Campbell Syst Rev. 2022 Jul 20;18(3):e1266. doi: 10.1002/cl2.1266 (PMC9300959; doi:10.1002/cl2.1266)
Supplement: Supplementary file 1 — Supporting information. [file CL2-18-e1266-s001.docx]

**ProQuest:**

**APA PsycArticles®‎ (1894 – current), APA PsycInfo®‎ (1806-current), Health & Medical Collection (1899-current)‎, MEDLINE®‎‎ (1946-current), Periodicals Archive Online (1854-2009)‎, Periodicals Index Online (1756-2021)‎, ProQuest Dissertations & Theses Global (1920-current), Psychology Database‎, (1957-current) and Publicly Available Content Database (1970-current)**

June 21-25, 2021

**noft**(radicali* OR terror* OR extremis* OR "lone wol*" OR lone-wol* OR "foreign fighter*" OR "single issue" OR Jihad* OR Islamis* OR Salaf* OR left-wing OR far-left OR right-wing OR far-right OR neo-nazi* OR communis* OR nationalis* OR supremacist OR anarch* OR indoctrinat*) AND **noft**(family OR families OR familial* OR parent* OR siblin* OR brother* OR sister* OR father* OR mother* OR child* OR son* OR daughter* OR cousin* OR uncle* OR aunt* OR generation* OR maternal OR paternal OR grandparent*) AND **noft**(risk* OR protect* OR factor OR correlat* OR relat* OR predict* OR caus* OR determina* OR consequenc* OR intervent* OR evaluat* OR program* OR treat* OR prevent* OR experiment* OR “cross-section*” OR longitudinal* OR regress*)

**Google Scholar**

**(1792-current)**

May 29-31, 2021

Google Scholar does not recognize truncation symbols, and full terms were included instead.

**allintitle:** radicalization family

**allintitle:** radicalization parent

**allintitle:** radicalization sibling

**allintitle:** radicalization brother

**allintitle:** radicalization sister

**allintitle:** radicalization father

**allintitle:** radicalization mother

**allintitle:** radicalization child

**allintitle:** radicalization son

**allintitle:** radicalization daughter

**allintitle:** radicalization cousin

**allintitle:** radicalization uncle

**allintitle:** radicalization aunt

**allintitle:** radicalization generation

**allintitle:** radicalisation family

**allintitle:** radicalisation parent

**allintitle:** radicalisation sibling

**allintitle:** radicalisation brother

**allintitle:** radicalisation sister

**allintitle:** radicalisation father

**allintitle:** radicalisation mother

**allintitle:** radicalisation child

**allintitle:** radicalisation son

**allintitle:** radicalisation daughter

**allintitle:** radicalisation cousin

**allintitle:** radicalisation uncle

**allintitle:** radicalisation aunt

**allintitle:** radicalisation generation

**allintitle:** terrorist family

**allintitle:** terrorist parent

**allintitle:** terrorist sibling

**allintitle:** terrorist brother

**allintitle:** terrorist sister

**allintitle:** terrorist father

**allintitle:** terrorist mother

**allintitle:** terrorist child

**allintitle:** terrorist son

**allintitle:** terrorist daughter

**allintitle:** terrorist cousin

**allintitle:** terrorist uncle

**allintitle:** terrorist aunt

**allintitle:** terrorist generation

**allintitle:** terrorism family

**allintitle:** terrorism parent

**allintitle:** terrorism sibling

**allintitle:** terrorism brother

**allintitle:** terrorism sister

**allintitle:** terrorism father

**allintitle:** terrorism mother

**allintitle:** terrorism child

**allintitle:** terrorism son

**allintitle:** terrorism daughter

**allintitle:** terrorism cousin

**allintitle:** terrorism uncle

**allintitle:** terrorism aunt

**allintitle:** terrorism generation

**allintitle:** extremist family

**allintitle:** extremist parent

**allintitle:** extremist sibling

**allintitle:** extremist brother

**allintitle:** extremist sister

**allintitle:** extremist father

**allintitle:** extremist mother

**allintitle:** extremist child

**allintitle:** extremist son

**allintitle:** extremist daughter

**allintitle:** extremist cousin

**allintitle:** extremist uncle

**allintitle:** extremist aunt

**allintitle:** extremist generation

**allintitle:** extremism family

**allintitle:** extremism parent

**allintitle:** extremism sibling

**allintitle:** extremism brother

**allintitle:** extremism sister

**allintitle:** extremism father

**allintitle:** extremism mother

**allintitle:** extremism child

**allintitle:** extremism son

**allintitle:** extremism daughter

**allintitle:** extremism cousin

**allintitle:** extremism uncle

**allintitle:** extremism aunt

**allintitle:** extremism generation

**allintitle:** lone-wolf family

**allintitle:** lone-wolf parent

**allintitle:** lone-wolf sibling

**allintitle:** lone-wolf brother

**allintitle:** lone-wolf sister

**allintitle:** lone-wolf father

**allintitle:** lone-wolf mother

**allintitle:** lone-wolf child

**allintitle:** lone-wolf son

**allintitle:** lone-wolf daughter

**allintitle:** lone-wolf cousin

**allintitle:** lone-wolf uncle

**allintitle:** lone-wolf aunt

**allintitle:** lone-wolf generation

**allintitle:** lone-wolves family

**allintitle:** lone-wolves parent

**allintitle:** lone-wolves sibling

**allintitle:** lone-wolves brother

**allintitle:** lone-wolves sister

**allintitle:** lone-wolves father

**allintitle:** lone-wolves mother

**allintitle:** lone-wolves child

**allintitle:** lone-wolves son

**allintitle:** lone-wolves daughter

**allintitle:** lone-wolves cousin

**allintitle:** lone-wolves uncle

**allintitle:** lone-wolves aunt

**allintitle:** lone-wolves generation

**allintitle:** foreign fighter family

**allintitle:** foreign fighter parent

**allintitle:** foreign fighter sibling

**allintitle:** foreign fighter brother

**allintitle:** foreign fighter sister

**allintitle:** foreign fighter father

**allintitle:** foreign fighter mother

**allintitle:** foreign fighter child

**allintitle:** foreign fighter son

**allintitle:** foreign fighter daughter

**allintitle:** foreign fighter cousin

**allintitle:** foreign fighter uncle

**allintitle:** foreign fighter aunt

**allintitle:** foreign fighter generation

**allintitle:** single issue family

**allintitle:** single issue parent

**allintitle:** single issue sibling

**allintitle:** single issue brother

**allintitle:** single issue sister

**allintitle:** single issue father

**allintitle:** single issue mother

**allintitle:** single issue child

**allintitle:** single issue son

**allintitle:** single issue daughter

**allintitle:** single issue cousin

**allintitle:** single issue uncle

**allintitle:** single issue aunt

**allintitle:** single issue generation

**allintitle:** Jihad family

**allintitle:** Jihad parent

**allintitle:** Jihad sibling

**allintitle:** Jihad brother

**allintitle:** Jihad sister

**allintitle:** Jihad father

**allintitle:** Jihad mother

**allintitle:** Jihad child

**allintitle:** Jihad son

**allintitle:** Jihad daughter

**allintitle:** Jihad cousin

**allintitle:** Jihad uncle

**allintitle:** Jihad aunt

**allintitle:** Jihad generation

**allintitle:** Islamist family

**allintitle:** Islamist parent

**allintitle:** Islamist sibling

**allintitle:** Islamist brother

**allintitle:** Islamist sister

**allintitle:** Islamist father

**allintitle:** Islamist mother

**allintitle:** Islamist child

**allintitle:** Islamist son

**allintitle:** Islamist daughter

**allintitle:** Islamist cousin

**allintitle:** Islamist uncle

**allintitle:** Islamist aunt

**allintitle:** Islamist generation

**allintitle:** Salafi family

**allintitle:** Salafi parent

**allintitle:** Salafi sibling

**allintitle:** Salafi brother

**allintitle:** Salafi sister

**allintitle:** Salafi father

**allintitle:** Salafi mother

**allintitle:** Salafi child

**allintitle:** Salafi son

**allintitle:** Salafi daughter

**allintitle:** Salafi cousin

**allintitle:** Salafi uncle

**allintitle:** Salafi aunt

**allintitle:** Salafi generation

**allintitle:** left-wing family

**allintitle:** left-wing parent

**allintitle:** left-wing sibling

**allintitle:** left-wing brother

**allintitle:** left-wing sister

**allintitle:** left-wing father

**allintitle:** left-wing mother

**allintitle:** left-wing child

**allintitle:** left-wing son

**allintitle:** left-wing daughter

**allintitle:** left-wing cousin

**allintitle:** left-wing uncle

**allintitle:** left-wing aunt

**allintitle:** left-wing generation

**allintitle:** right-wing family

**allintitle:** right-wing parent

**allintitle:** right-wing sibling

**allintitle:** right-wing brother

**allintitle:** right-wing sister

**allintitle:** right-wing father

**allintitle:** right-wing mother

**allintitle:** right-wing child

**allintitle:** right-wing son

**allintitle:** right-wing daughter

**allintitle:** right-wing cousin

**allintitle:** right-wing uncle

**allintitle:** right-wing aunt

**allintitle:** right-wing generation

**allintitle:** far-left family

**allintitle:** far-left parent

**allintitle:** far-left sibling

**allintitle:** far-left brother

**allintitle:** far-left sister

**allintitle:** far-left father

**allintitle:** far-left mother

**allintitle:** far-left child

**allintitle:** far-left son

**allintitle:** far-left daughter

**allintitle:** far-left cousin

**allintitle:** far-left uncle

**allintitle:** far-left aunt

**allintitle:** far-left generation

**allintitle:** far-right family

**allintitle:** far-right parent

**allintitle:** far-right sibling

**allintitle:** far-right brother

**allintitle:** far-right sister

**allintitle:** far-right father

**allintitle:** far-right mother

**allintitle:** far-right child

**allintitle:** far-right son

**allintitle:** far-right daughter

**allintitle:** far-right cousin

**allintitle:** far-right uncle

**allintitle:** far-right aunt

**allintitle:** far-right generation

**allintitle:** neo-nazi family

**allintitle:** neo-nazi parent

**allintitle:** neo-nazi sibling

**allintitle:** neo-nazi brother

**allintitle:** neo-nazi sister

**allintitle:** neo-nazi father

**allintitle:** neo-nazi mother

**allintitle:** neo-nazi child

**allintitle:** neo-nazi son

**allintitle:** neo-nazi daughter

**allintitle:** neo-nazi cousin

**allintitle:** neo-nazi uncle

**allintitle:** neo-nazi aunt

**allintitle:** neo-nazi generation

**allintitle:** communist family

**allintitle:** communist parent

**allintitle:** communist sibling

**allintitle:** communist brother

**allintitle:** communist sister

**allintitle:** communist father

**allintitle:** communist mother

**allintitle:** communist child

**allintitle:** communist son

**allintitle:** communist daughter

**allintitle:** communist cousin

**allintitle:** communist uncle

**allintitle:** communist aunt

**allintitle:** communist generation

**allintitle:** nationalist family

**allintitle:** nationalist parent

**allintitle:** nationalist sibling

**allintitle:** nationalist brother

**allintitle:** nationalist sister

**allintitle:** nationalist father

**allintitle:** nationalist mother

**allintitle:** nationalist child

**allintitle:** nationalist son

**allintitle:** nationalist daughter

**allintitle:** nationalist cousin

**allintitle:** nationalist uncle

**allintitle:** nationalist aunt

**allintitle:** nationalist generation

**allintitle:** supremacist family

**allintitle:** supremacist parent

**allintitle:** supremacist sibling

**allintitle:** supremacist brother

**allintitle:** supremacist sister

**allintitle:** supremacist father

**allintitle:** supremacist mother

**allintitle:** supremacist child

**allintitle:** supremacist son

**allintitle:** supremacist daughter

**allintitle:** supremacist cousin

**allintitle:** supremacist uncle

**allintitle:** supremacist aunt

**allintitle:** supremacist generation

**allintitle:** anarchist family

**allintitle:** anarchist parent

**allintitle:** anarchist sibling

**allintitle:** anarchist brother

**allintitle:** anarchist sister

**allintitle:** anarchist father

**allintitle:** anarchist mother

**allintitle:** anarchist child

**allintitle:** anarchist son

**allintitle:** anarchist daughter

**allintitle:** anarchist cousin

**allintitle:** anarchist uncle

**allintitle:** anarchist aunt

**allintitle:** anarchist generation

**allintitle:** indoctrination family

**allintitle:** indoctrination parent

**allintitle:** indoctrination sibling

**allintitle:** indoctrination brother

**allintitle:** indoctrination sister

**allintitle:** indoctrination father

**allintitle:** indoctrination mother

**allintitle:** indoctrination child

**allintitle:** indoctrination son

**allintitle:** indoctrination daughter

**allintitle:** indoctrination cousin

**allintitle:** indoctrination uncle

**allintitle:** indoctrination aunt

**allintitle:** indoctrination generation

**Sage Journals Online (1847-current)**

June 26-27, 2021

for [[Title radicali*] OR [Title terror*] OR [Title extremis*] OR [Title "lone wol*"] OR [Title lone-wol*] OR [Title "foreign fighter*"] OR [Title "single issue"] OR [Title jihad*] OR [Title islamis*] OR [Title salaf*] OR [Title left-wing] OR [Title far-left] OR [Title right-wing] OR [Title far-right] OR [Title neo-nazi*] OR [Title communis*] OR [Title nationalis*] OR [Title supremacist] OR [Title anarch*] OR [Title indoctrinat*]] AND [[Title family] OR [Title families] OR [Title familial*] OR [Title parent*] OR [Title siblin*] OR [Title brother*] OR [Title sister*] OR [Title father*] OR [Title mother*] OR [Title child*] OR [Title son*] OR [Title daughter*] OR [Title cousin*] OR [Title uncle*] OR [Title aunt*] OR [Title generation*] OR [Title maternal] OR [Title paternal] OR [Title grandparent*]] AND [[Keywords risk*] OR [Keywords protect*] OR [Keywords factor*] OR [Keywords correlat*] OR [Keywords relat*] OR [Keywords predict*] OR [Keywords caus*] OR [Keywords determina*] OR [Keywords consequenc*] OR [Keywords interven*] OR [Keywords evaluat*] OR [Keywords program*] OR [Keywords treat*] OR [Keywords prevent*] OR [Keywords experiment*] OR [Keywords "cross-section*"] OR [Keywords longitudinal*] OR [Keywords regress*]]

for [[Title radicali*] OR [Title terror*] OR [Title extremis*] OR [Title "lone wol*"] OR [Title lone-wol*] OR [Title "foreign fighter*"] OR [Title "single issue"] OR [Title jihad*] OR [Title islamis*] OR [Title salaf*] OR [Title left-wing] OR [Title far-left] OR [Title right-wing] OR [Title far-right] OR [Title neo-nazi*] OR [Title communis*] OR [Title nationalis*] OR [Title supremacist] OR [Title anarch*] OR [Title indoctrinat*]] AND [[Keywords family] OR [Keywords families] OR [Keywords familial*] OR [Keywords parent*] OR [Keywords siblin*] OR [Keywords brother*] OR [Keywords sister*] OR [Keywords father*] OR [Keywords mother*] OR [Keywords child*] OR [Keywords son*] OR [Keywords daughter*] OR [Keywords cousin*] OR [Keywords uncle*] OR [Keywords aunt*] OR [Keywords generation*] OR [Keywords maternal] OR [Keywords paternal] OR [Keywords grandparent*]] AND [[Title risk*] OR [Title protect*] OR [Title factor*] OR [Title correlat*] OR [Title relat*] OR [Title predict*] OR [Title caus*] OR [Title determina*] OR [Title consequenc*] OR [Title interven*] OR [Title evaluat*] OR [Title program*] OR [Title treat*] OR [Title prevent*] OR [Title experiment*] OR [Title "cross-section*"] OR [Title longitudinal*] OR [Title regress*]]

for [[Title radicali*] OR [Title terror*] OR [Title extremis*] OR [Title "lone wol*"] OR [Title lone-wol*] OR [Title "foreign fighter*"] OR [Title "single issue"] OR [Title jihad*] OR [Title islamis*] OR [Title salaf*] OR [Title left-wing] OR [Title far-left] OR [Title right-wing] OR [Title far-right] OR [Title neo-nazi*] OR [Title communis*] OR [Title nationalis*] OR [Title supremacist] OR [Title anarch*] OR [Title indoctrinat*]] AND [[Keywords family] OR [Keywords families] OR [Keywords familial*] OR [Keywords parent*] OR [Keywords siblin*] OR [Keywords brother*] OR [Keywords sister*] OR [Keywords father*] OR [Keywords mother*] OR [Keywords child*] OR [Keywords son*] OR [Keywords daughter*] OR [Keywords cousin*] OR [Keywords uncle*] OR [Keywords aunt*] OR [Keywords generation*] OR [Keywords maternal] OR [Keywords paternal] OR [Keywords grandparent*]] AND [[Keywords risk*] OR [Keywords protect*] OR [Keywords factor*] OR [Keywords correlat*] OR [Keywords relat*] OR [Keywords predict*] OR [Keywords caus*] OR [Keywords determina*] OR [Keywords consequenc*] OR [Keywords interven*] OR [Keywords evaluat*] OR [Keywords program*] OR [Keywords treat*] OR [Keywords prevent*] OR [Keywords experiment*] OR [Keywords "cross-section*"] OR [Keywords longitudinal*] OR [Keywords regress*]]

for [[Keywords radicali*] OR [Keywords terror*] OR [Keywords extremis*] OR [Keywords "lone wol*"] OR [Keywords lone-wol*] OR [Keywords "foreign fighter*"] OR [Keywords "single issue"] OR [Keywords jihad*] OR [Keywords islamis*] OR [Keywords salaf*] OR [Keywords left-wing] OR [Keywords far-left] OR [Keywords right-wing] OR [Keywords far-right] OR [Keywords neo-nazi*] OR [Keywords communis*] OR [Keywords nationalis*] OR [Keywords supremacist] OR [Keywords anarch*] OR [Keywords indoctrinat*]] AND [[Title family] OR [Title families] OR [Title familial*] OR [Title parent*] OR [Title siblin*] OR [Title brother*] OR [Title sister*] OR [Title father*] OR [Title mother*] OR [Title child*] OR [Title son*] OR [Title daughter*] OR [Title cousin*] OR [Title uncle*] OR [Title aunt*] OR [Title generation*] OR [Title maternal] OR [Title paternal] OR [Title grandparent*]] AND [[Title risk*] OR [Title protect*] OR [Title factor*] OR [Title correlat*] OR [Title relat*] OR [Title predict*] OR [Title caus*] OR [Title determina*] OR [Title consequenc*] OR [Title interven*] OR [Title evaluat*] OR [Title program*] OR [Title treat*] OR [Title prevent*] OR [Title experiment*] OR [Title "cross-section*"] OR [Title longitudinal*] OR [Title regress*]]

for [[Keywords radicali*] OR [Keywords terror*] OR [Keywords extremis*] OR [Keywords "lone wol*"] OR [Keywords lone-wol*] OR [Keywords "foreign fighter*"] OR [Keywords "single issue"] OR [Keywords jihad*] OR [Keywords islamis*] OR [Keywords salaf*] OR [Keywords left-wing] OR [Keywords far-left] OR [Keywords right-wing] OR [Keywords far-right] OR [Keywords neo-nazi*] OR [Keywords communis*] OR [Keywords nationalis*] OR [Keywords supremacist] OR [Keywords anarch*] OR [Keywords indoctrinat*]] AND [[Keywords family] OR [Keywords families] OR [Keywords familial*] OR [Keywords parent*] OR [Keywords siblin*] OR [Keywords brother*] OR [Keywords sister*] OR [Keywords father*] OR [Keywords mother*] OR [Keywords child*] OR [Keywords son*] OR [Keywords daughter*] OR [Keywords cousin*] OR [Keywords uncle*] OR [Keywords aunt*] OR [Keywords generation*] OR [Keywords maternal] OR [Keywords paternal] OR [Keywords grandparent*]] AND [[Keywords risk*] OR [Keywords protect*] OR [Keywords factor*] OR [Keywords correlat*] OR [Keywords relat*] OR [Keywords predict*] OR [Keywords caus*] OR [Keywords determina*] OR [Keywords consequenc*] OR [Keywords interven*] OR [Keywords evaluat*] OR [Keywords program*] OR [Keywords treat*] OR [Keywords prevent*] OR [Keywords experiment*] OR [Keywords "cross-section*"] OR [Keywords longitudinal*] OR [Keywords regress*]]

for [[Title radicali*] OR [Title terror*] OR [Title extremis*] OR [Title "lone wol*"] OR [Title lone-wol*] OR [Title "foreign fighter*"] OR [Title "single issue"] OR [Title jihad*] OR [Title islamis*] OR [Title salaf*] OR [Title left-wing] OR [Title far-left] OR [Title right-wing] OR [Title far-right] OR [Title neo-nazi*] OR [Title communis*] OR [Title nationalis*] OR [Title supremacist] OR [Title anarch*] OR [Title indoctrinat*]] AND [[Title family] OR [Title families] OR [Title familial*] OR [Title parent*] OR [Title siblin*] OR [Title brother*] OR [Title sister*] OR [Title father*] OR [Title mother*] OR [Title child*] OR [Title son*] OR [Title daughter*] OR [Title cousin*] OR [Title uncle*] OR [Title aunt*] OR [Title generation*] OR [Title maternal] OR [Title paternal] OR [Title grandparent*]] AND [[Title risk*] OR [Title protect*] OR [Title factor*] OR [Title correlat*] OR [Title relat*] OR [Title predict*] OR [Title caus*] OR [Title determina*] OR [Title consequenc*] OR [Title interven*] OR [Title evaluat*] OR [Title program*] OR [Title treat*] OR [Title prevent*] OR [Title experiment*] OR [Title "cross-section*"] OR [Title longitudinal*] OR [Title regress*]]

for [[Title radicali*] OR [Title terror*] OR [Title extremis*] OR [Title "lone wol*"] OR [Title lone-wol*] OR [Title "foreign fighter*"] OR [Title "single issue"] OR [Title jihad*] OR [Title islamis*] OR [Title salaf*] OR [Title left-wing] OR [Title far-left] OR [Title right-wing] OR [Title far-right] OR [Title neo-nazi*] OR [Title communis*] OR [Title nationalis*] OR [Title supremacist] OR [Title anarch*] OR [Title indoctrinat*]] AND [[Title family] OR [Title families] OR [Title familial*] OR [Title parent*] OR [Title siblin*] OR [Title brother*] OR [Title sister*] OR [Title father*] OR [Title mother*] OR [Title child*] OR [Title son*] OR [Title daughter*] OR [Title cousin*] OR [Title uncle*] OR [Title aunt*] OR [Title generation*] OR [Title maternal] OR [Title paternal] OR [Title grandparent*]] AND [[Abstract risk*] OR [Abstract protect*] OR [Abstract factor*] OR [Abstract correlat*] OR [Abstract relat*] OR [Abstract predict*] OR [Abstract caus*] OR [Abstract determina*] OR [Abstract consequenc*] OR [Abstract interven*] OR [Abstract evaluat*] OR [Abstract program*] OR [Abstract treat*] OR [Abstract prevent*] OR [Abstract experiment*] OR [Abstract "cross-section*"] OR [Abstract longitudinal*] OR [Abstract regress*]]

for [[Title radicali*] OR [Title terror*] OR [Title extremis*] OR [Title "lone wol*"] OR [Title lone-wol*] OR [Title "foreign fighter*"] OR [Title "single issue"] OR [Title jihad*] OR [Title islamis*] OR [Title salaf*] OR [Title left-wing] OR [Title far-left] OR [Title right-wing] OR [Title far-right] OR [Title neo-nazi*] OR [Title communis*] OR [Title nationalis*] OR [Title supremacist] OR [Title anarch*] OR [Title indoctrinat*]] AND [[Keywords family] OR [Keywords families] OR [Keywords familial*] OR [Keywords parent*] OR [Keywords siblin*] OR [Keywords brother*] OR [Keywords sister*] OR [Keywords father*] OR [Keywords mother*] OR [Keywords child*] OR [Keywords son*] OR [Keywords daughter*] OR [Keywords cousin*] OR [Keywords uncle*] OR [Keywords aunt*] OR [Keywords generation*] OR [Keywords maternal] OR [Keywords paternal] OR [Keywords grandparent*]] AND [[Abstract risk*] OR [Abstract protect*] OR [Abstract factor*] OR [Abstract correlat*] OR [Abstract relat*] OR [Abstract predict*] OR [Abstract caus*] OR [Abstract determina*] OR [Abstract consequenc*] OR [Abstract interven*] OR [Abstract evaluat*] OR [Abstract program*] OR [Abstract treat*] OR [Abstract prevent*] OR [Abstract experiment*] OR [Abstract "cross-section*"] OR [Abstract longitudinal*] OR [Abstract regress*]]

for [[Abstract radicali*] OR [Abstract terror*] OR [Abstract extremis*] OR [Abstract "lone wol*"] OR [Abstract lone-wol*] OR [Abstract "foreign fighter*"] OR [Abstract "single issue"] OR [Abstract jihad*] OR [Abstract islamis*] OR [Abstract salaf*] OR [Abstract left-wing] OR [Abstract far-left] OR [Abstract right-wing] OR [Abstract far-right] OR [Abstract neo-nazi*] OR [Abstract communis*] OR [Abstract nationalis*] OR [Abstract supremacist] OR [Abstract anarch*] OR [Abstract indoctrinat*]] AND [[Abstract family] OR [Abstract families] OR [Abstract familial*] OR [Abstract parent*] OR [Abstract siblin*] OR [Abstract brother*] OR [Abstract sister*] OR [Abstract father*] OR [Abstract mother*] OR [Abstract child*] OR [Abstract son*] OR [Abstract daughter*] OR [Abstract cousin*] OR [Abstract uncle*] OR [Abstract aunt*] OR [Abstract generation*] OR [Abstract maternal] OR [Abstract paternal] OR [Abstract grandparent*]] AND [[Abstract risk*] OR [Abstract protect*] OR [Abstract factor*] OR [Abstract correlat*] OR [Abstract relat*] OR [Abstract predict*] OR [Abstract caus*] OR [Abstract determina*] OR [Abstract consequenc*] OR [Abstract interven*] OR [Abstract evaluat*] OR [Abstract program*] OR [Abstract treat*] OR [Abstract prevent*] OR [Abstract experiment*] OR [Abstract "cross-section*"] OR [Abstract longitudinal*] OR [Abstract regress*]]

for [[Title radicali*] OR [Title terror*] OR [Title extremis*] OR [Title "lone wol*"] OR [Title lone-wol*] OR [Title "foreign fighter*"] OR [Title "single issue"] OR [Title jihad*] OR [Title islamis*] OR [Title salaf*] OR [Title left-wing] OR [Title far-left] OR [Title right-wing] OR [Title far-right] OR [Title neo-nazi*] OR [Title communis*] OR [Title nationalis*] OR [Title supremacist] OR [Title anarch*] OR [Title indoctrinat*]] AND [[Abstract family] OR [Abstract families] OR [Abstract familial*] OR [Abstract parent*] OR [Abstract siblin*] OR [Abstract brother*] OR [Abstract sister*] OR [Abstract father*] OR [Abstract mother*] OR [Abstract child*] OR [Abstract son*] OR [Abstract daughter*] OR [Abstract cousin*] OR [Abstract uncle*] OR [Abstract aunt*] OR [Abstract generation*] OR [Abstract maternal] OR [Abstract paternal] OR [Abstract grandparent*]] AND [[Abstract risk*] OR [Abstract protect*] OR [Abstract factor*] OR [Abstract correlat*] OR [Abstract relat*] OR [Abstract predict*] OR [Abstract caus*] OR [Abstract determina*] OR [Abstract consequenc*] OR [Abstract interven*] OR [Abstract evaluat*] OR [Abstract program*] OR [Abstract treat*] OR [Abstract prevent*] OR [Abstract experiment*] OR [Abstract "cross-section*"] OR [Abstract longitudinal*] OR [Abstract regress*]]

for [[Abstract radicali*] OR [Abstract terror*] OR [Abstract extremis*] OR [Abstract "lone wol*"] OR [Abstract lone-wol*] OR [Abstract "foreign fighter*"] OR [Abstract "single issue"] OR [Abstract jihad*] OR [Abstract islamis*] OR [Abstract salaf*] OR [Abstract left-wing] OR [Abstract far-left] OR [Abstract right-wing] OR [Abstract far-right] OR [Abstract neo-nazi*] OR [Abstract communis*] OR [Abstract nationalis*] OR [Abstract supremacist] OR [Abstract anarch*] OR [Abstract indoctrinat*]] AND [[Title family] OR [Title families] OR [Title familial*] OR [Title parent*] OR [Title siblin*] OR [Title brother*] OR [Title sister*] OR [Title father*] OR [Title mother*] OR [Title child*] OR [Title son*] OR [Title daughter*] OR [Title cousin*] OR [Title uncle*] OR [Title aunt*] OR [Title generation*] OR [Title maternal] OR [Title paternal] OR [Title grandparent*]] AND [[Abstract risk*] OR [Abstract protect*] OR [Abstract factor*] OR [Abstract correlat*] OR [Abstract relat*] OR [Abstract predict*] OR [Abstract caus*] OR [Abstract determina*] OR [Abstract consequenc*] OR [Abstract interven*] OR [Abstract evaluat*] OR [Abstract program*] OR [Abstract treat*] OR [Abstract prevent*] OR [Abstract experiment*] OR [Abstract "cross-section*"] OR [Abstract longitudinal*] OR [Abstract regress*]]

for [[Abstract radicali*] OR [Abstract terror*] OR [Abstract extremis*] OR [Abstract "lone wol*"] OR [Abstract lone-wol*] OR [Abstract "foreign fighter*"] OR [Abstract "single issue"] OR [Abstract jihad*] OR [Abstract islamis*] OR [Abstract salaf*] OR [Abstract left-wing] OR [Abstract far-left] OR [Abstract right-wing] OR [Abstract far-right] OR [Abstract neo-nazi*] OR [Abstract communis*] OR [Abstract nationalis*] OR [Abstract supremacist] OR [Abstract anarch*] OR [Abstract indoctrinat*]] AND [[Abstract family] OR [Abstract families] OR [Abstract familial*] OR [Abstract parent*] OR [Abstract siblin*] OR [Abstract brother*] OR [Abstract sister*] OR [Abstract father*] OR [Abstract mother*] OR [Abstract child*] OR [Abstract son*] OR [Abstract daughter*] OR [Abstract cousin*] OR [Abstract uncle*] OR [Abstract aunt*] OR [Abstract generation*] OR [Abstract maternal] OR [Abstract paternal] OR [Abstract grandparent*]] AND [[Title risk*] OR [Title protect*] OR [Title factor*] OR [Title correlat*] OR [Title relat*] OR [Title predict*] OR [Title caus*] OR [Title determina*] OR [Title consequenc*] OR [Title interven*] OR [Title evaluat*] OR [Title program*] OR [Title treat*] OR [Title prevent*] OR [Title experiment*] OR [Title "cross-section*"] OR [Title longitudinal*] OR [Title regress*]]

for [[Keywords radicali*] OR [Keywords terror*] OR [Keywords extremis*] OR [Keywords "lone wol*"] OR [Keywords lone-wol*] OR [Keywords "foreign fighter*"] OR [Keywords "single issue"] OR [Keywords jihad*] OR [Keywords islamis*] OR [Keywords salaf*] OR [Keywords left-wing] OR [Keywords far-left] OR [Keywords right-wing] OR [Keywords far-right] OR [Keywords neo-nazi*] OR [Keywords communis*] OR [Keywords nationalis*] OR [Keywords supremacist] OR [Keywords anarch*] OR [Keywords indoctrinat*]] AND [[Keywords family] OR [Keywords families] OR [Keywords familial*] OR [Keywords parent*] OR [Keywords siblin*] OR [Keywords brother*] OR [Keywords sister*] OR [Keywords father*] OR [Keywords mother*] OR [Keywords child*] OR [Keywords son*] OR [Keywords daughter*] OR [Keywords cousin*] OR [Keywords uncle*] OR [Keywords aunt*] OR [Keywords generation*] OR [Keywords maternal] OR [Keywords paternal] OR [Keywords grandparent*]] AND [[Title risk*] OR [Title protect*] OR [Title factor*] OR [Title correlat*] OR [Title relat*] OR [Title predict*] OR [Title caus*] OR [Title determina*] OR [Title consequenc*] OR [Title interven*] OR [Title evaluat*] OR [Title program*] OR [Title treat*] OR [Title prevent*] OR [Title experiment*] OR [Title "cross-section*"] OR [Title longitudinal*] OR [Title regress*]]

for [[Keywords radicali*] OR [Keywords terror*] OR [Keywords extremis*] OR [Keywords "lone wol*"] OR [Keywords lone-wol*] OR [Keywords "foreign fighter*"] OR [Keywords "single issue"] OR [Keywords jihad*] OR [Keywords islamis*] OR [Keywords salaf*] OR [Keywords left-wing] OR [Keywords far-left] OR [Keywords right-wing] OR [Keywords far-right] OR [Keywords neo-nazi*] OR [Keywords communis*] OR [Keywords nationalis*] OR [Keywords supremacist] OR [Keywords anarch*] OR [Keywords indoctrinat*]] AND [[Title family] OR [Title families] OR [Title familial*] OR [Title parent*] OR [Title siblin*] OR [Title brother*] OR [Title sister*] OR [Title father*] OR [Title mother*] OR [Title child*] OR [Title son*] OR [Title daughter*] OR [Title cousin*] OR [Title uncle*] OR [Title aunt*] OR [Title generation*] OR [Title maternal] OR [Title paternal] OR [Title grandparent*]] AND [[Keywords risk*] OR [Keywords protect*] OR [Keywords factor*] OR [Keywords correlat*] OR [Keywords relat*] OR [Keywords predict*] OR [Keywords caus*] OR [Keywords determina*] OR [Keywords consequenc*] OR [Keywords interven*] OR [Keywords evaluat*] OR [Keywords program*] OR [Keywords treat*] OR [Keywords prevent*] OR [Keywords experiment*] OR [Keywords "cross-section*"] OR [Keywords longitudinal*] OR [Keywords regress*]]

for [[Keywords radicali*] OR [Keywords terror*] OR [Keywords extremis*] OR [Keywords "lone wol*"] OR [Keywords lone-wol*] OR [Keywords "foreign fighter*"] OR [Keywords "single issue"] OR [Keywords jihad*] OR [Keywords islamis*] OR [Keywords salaf*] OR [Keywords left-wing] OR [Keywords far-left] OR [Keywords right-wing] OR [Keywords far-right] OR [Keywords neo-nazi*] OR [Keywords communis*] OR [Keywords nationalis*] OR [Keywords supremacist] OR [Keywords anarch*] OR [Keywords indoctrinat*]] AND [[Abstract family] OR [Abstract families] OR [Abstract familial*] OR [Abstract parent*] OR [Abstract siblin*] OR [Abstract brother*] OR [Abstract sister*] OR [Abstract father*] OR [Abstract mother*] OR [Abstract child*] OR [Abstract son*] OR [Abstract daughter*] OR [Abstract cousin*] OR [Abstract uncle*] OR [Abstract aunt*] OR [Abstract generation*] OR [Abstract maternal] OR [Abstract paternal] OR [Abstract grandparent*]] AND [[Abstract risk*] OR [Abstract protect*] OR [Abstract factor*] OR [Abstract correlat*] OR [Abstract relat*] OR [Abstract predict*] OR [Abstract caus*] OR [Abstract determina*] OR [Abstract consequenc*] OR [Abstract interven*] OR [Abstract evaluat*] OR [Abstract program*] OR [Abstract treat*] OR [Abstract prevent*] OR [Abstract experiment*] OR [Abstract "cross-section*"] OR [Abstract longitudinal*] OR [Abstract regress*]]

for [[Abstract radicali*] OR [Abstract terror*] OR [Abstract extremis*] OR [Abstract "lone wol*"] OR [Abstract lone-wol*] OR [Abstract "foreign fighter*"] OR [Abstract "single issue"] OR [Abstract jihad*] OR [Abstract islamis*] OR [Abstract salaf*] OR [Abstract left-wing] OR [Abstract far-left] OR [Abstract right-wing] OR [Abstract far-right] OR [Abstract neo-nazi*] OR [Abstract communis*] OR [Abstract nationalis*] OR [Abstract supremacist] OR [Abstract anarch*] OR [Abstract indoctrinat*]] AND [[Keywords family] OR [Keywords families] OR [Keywords familial*] OR [Keywords parent*] OR [Keywords siblin*] OR [Keywords brother*] OR [Keywords sister*] OR [Keywords father*] OR [Keywords mother*] OR [Keywords child*] OR [Keywords son*] OR [Keywords daughter*] OR [Keywords cousin*] OR [Keywords uncle*] OR [Keywords aunt*] OR [Keywords generation*] OR [Keywords maternal] OR [Keywords paternal] OR [Keywords grandparent*]] AND [[Abstract risk*] OR [Abstract protect*] OR [Abstract factor*] OR [Abstract correlat*] OR [Abstract relat*] OR [Abstract predict*] OR [Abstract caus*] OR [Abstract determina*] OR [Abstract consequenc*] OR [Abstract interven*] OR [Abstract evaluat*] OR [Abstract program*] OR [Abstract treat*] OR [Abstract prevent*] OR [Abstract experiment*] OR [Abstract "cross-section*"] OR [Abstract longitudinal*] OR [Abstract regress*]]

for [[Abstract radicali*] OR [Abstract terror*] OR [Abstract extremis*] OR [Abstract "lone wol*"] OR [Abstract lone-wol*] OR [Abstract "foreign fighter*"] OR [Abstract "single issue"] OR [Abstract jihad*] OR [Abstract islamis*] OR [Abstract salaf*] OR [Abstract left-wing] OR [Abstract far-left] OR [Abstract right-wing] OR [Abstract far-right] OR [Abstract neo-nazi*] OR [Abstract communis*] OR [Abstract nationalis*] OR [Abstract supremacist] OR [Abstract anarch*] OR [Abstract indoctrinat*]] AND [[Abstract family] OR [Abstract families] OR [Abstract familial*] OR [Abstract parent*] OR [Abstract siblin*] OR [Abstract brother*] OR [Abstract sister*] OR [Abstract father*] OR [Abstract mother*] OR [Abstract child*] OR [Abstract son*] OR [Abstract daughter*] OR [Abstract cousin*] OR [Abstract uncle*] OR [Abstract aunt*] OR [Abstract generation*] OR [Abstract maternal] OR [Abstract paternal] OR [Abstract grandparent*]] AND [[Keywords risk*] OR [Keywords protect*] OR [Keywords factor*] OR [Keywords correlat*] OR [Keywords relat*] OR [Keywords predict*] OR [Keywords caus*] OR [Keywords determina*] OR [Keywords consequenc*] OR [Keywords interven*] OR [Keywords evaluat*] OR [Keywords program*] OR [Keywords treat*] OR [Keywords prevent*] OR [Keywords experiment*] OR [Keywords "cross-section*"] OR [Keywords longitudinal*] OR [Keywords regress*]]

for [[Keywords radicali*] OR [Keywords terror*] OR [Keywords extremis*] OR [Keywords "lone wol*"] OR [Keywords lone-wol*] OR [Keywords "foreign fighter*"] OR [Keywords "single issue"] OR [Keywords jihad*] OR [Keywords islamis*] OR [Keywords salaf*] OR [Keywords left-wing] OR [Keywords far-left] OR [Keywords right-wing] OR [Keywords far-right] OR [Keywords neo-nazi*] OR [Keywords communis*] OR [Keywords nationalis*] OR [Keywords supremacist] OR [Keywords anarch*] OR [Keywords indoctrinat*]] AND [[Keywords family] OR [Keywords families] OR [Keywords familial*] OR [Keywords parent*] OR [Keywords siblin*] OR [Keywords brother*] OR [Keywords sister*] OR [Keywords father*] OR [Keywords mother*] OR [Keywords child*] OR [Keywords son*] OR [Keywords daughter*] OR [Keywords cousin*] OR [Keywords uncle*] OR [Keywords aunt*] OR [Keywords generation*] OR [Keywords maternal] OR [Keywords paternal] OR [Keywords grandparent*]] AND [[Abstract risk*] OR [Abstract protect*] OR [Abstract factor*] OR [Abstract correlat*] OR [Abstract relat*] OR [Abstract predict*] OR [Abstract caus*] OR [Abstract determina*] OR [Abstract consequenc*] OR [Abstract interven*] OR [Abstract evaluat*] OR [Abstract program*] OR [Abstract treat*] OR [Abstract prevent*] OR [Abstract experiment*] OR [Abstract "cross-section*"] OR [Abstract longitudinal*] OR [Abstract regress*]]

for [[Keywords radicali*] OR [Keywords terror*] OR [Keywords extremis*] OR [Keywords "lone wol*"] OR [Keywords lone-wol*] OR [Keywords "foreign fighter*"] OR [Keywords "single issue"] OR [Keywords jihad*] OR [Keywords islamis*] OR [Keywords salaf*] OR [Keywords left-wing] OR [Keywords far-left] OR [Keywords right-wing] OR [Keywords far-right] OR [Keywords neo-nazi*] OR [Keywords communis*] OR [Keywords nationalis*] OR [Keywords supremacist] OR [Keywords anarch*] OR [Keywords indoctrinat*]] AND [[Abstract family] OR [Abstract families] OR [Abstract familial*] OR [Abstract parent*] OR [Abstract siblin*] OR [Abstract brother*] OR [Abstract sister*] OR [Abstract father*] OR [Abstract mother*] OR [Abstract child*] OR [Abstract son*] OR [Abstract daughter*] OR [Abstract cousin*] OR [Abstract uncle*] OR [Abstract aunt*] OR [Abstract generation*] OR [Abstract maternal] OR [Abstract paternal] OR [Abstract grandparent*]] AND [[Keywords risk*] OR [Keywords protect*] OR [Keywords factor*] OR [Keywords correlat*] OR [Keywords relat*] OR [Keywords predict*] OR [Keywords caus*] OR [Keywords determina*] OR [Keywords consequenc*] OR [Keywords interven*] OR [Keywords evaluat*] OR [Keywords program*] OR [Keywords treat*] OR [Keywords prevent*] OR [Keywords experiment*] OR [Keywords "cross-section*"] OR [Keywords longitudinal*] OR [Keywords regress*]]

for [[Abstract radicali*] OR [Abstract terror*] OR [Abstract extremis*] OR [Abstract "lone wol*"] OR [Abstract lone-wol*] OR [Abstract "foreign fighter*"] OR [Abstract "single issue"] OR [Abstract jihad*] OR [Abstract islamis*] OR [Abstract salaf*] OR [Abstract left-wing] OR [Abstract far-left] OR [Abstract right-wing] OR [Abstract far-right] OR [Abstract neo-nazi*] OR [Abstract communis*] OR [Abstract nationalis*] OR [Abstract supremacist] OR [Abstract anarch*] OR [Abstract indoctrinat*]] AND [[Keywords family] OR [Keywords families] OR [Keywords familial*] OR [Keywords parent*] OR [Keywords siblin*] OR [Keywords brother*] OR [Keywords sister*] OR [Keywords father*] OR [Keywords mother*] OR [Keywords child*] OR [Keywords son*] OR [Keywords daughter*] OR [Keywords cousin*] OR [Keywords uncle*] OR [Keywords aunt*] OR [Keywords generation*] OR [Keywords maternal] OR [Keywords paternal] OR [Keywords grandparent*]] AND [[Keywords risk*] OR [Keywords protect*] OR [Keywords factor*] OR [Keywords correlat*] OR [Keywords relat*] OR [Keywords predict*] OR [Keywords caus*] OR [Keywords determina*] OR [Keywords consequenc*] OR [Keywords interven*] OR [Keywords evaluat*] OR [Keywords program*] OR [Keywords treat*] OR [Keywords prevent*] OR [Keywords experiment*] OR [Keywords "cross-section*"] OR [Keywords longitudinal*] OR [Keywords regress*]]

for [[Title radicali*] OR [Title terror*] OR [Title extremis*] OR [Title "lone wol*"] OR [Title lone-wol*] OR [Title "foreign fighter*"] OR [Title "single issue"] OR [Title jihad*] OR [Title islamis*] OR [Title salaf*] OR [Title left-wing] OR [Title far-left] OR [Title right-wing] OR [Title far-right] OR [Title neo-nazi*] OR [Title communis*] OR [Title nationalis*] OR [Title supremacist] OR [Title anarch*] OR [Title indoctrinat*]] AND [[Abstract family] OR [Abstract families] OR [Abstract familial*] OR [Abstract parent*] OR [Abstract siblin*] OR [Abstract brother*] OR [Abstract sister*] OR [Abstract father*] OR [Abstract mother*] OR [Abstract child*] OR [Abstract son*] OR [Abstract daughter*] OR [Abstract cousin*] OR [Abstract uncle*] OR [Abstract aunt*] OR [Abstract generation*] OR [Abstract maternal] OR [Abstract paternal] OR [Abstract grandparent*]] AND [[Keywords risk*] OR [Keywords protect*] OR [Keywords factor*] OR [Keywords correlat*] OR [Keywords relat*] OR [Keywords predict*] OR [Keywords caus*] OR [Keywords determina*] OR [Keywords consequenc*] OR [Keywords interven*] OR [Keywords evaluat*] OR [Keywords program*] OR [Keywords treat*] OR [Keywords prevent*] OR [Keywords experiment*] OR [Keywords "cross-section*"] OR [Keywords longitudinal*] OR [Keywords regress*]]

for [[Abstract radicali*] OR [Abstract terror*] OR [Abstract extremis*] OR [Abstract "lone wol*"] OR [Abstract lone-wol*] OR [Abstract "foreign fighter*"] OR [Abstract "single issue"] OR [Abstract jihad*] OR [Abstract islamis*] OR [Abstract salaf*] OR [Abstract left-wing] OR [Abstract far-left] OR [Abstract right-wing] OR [Abstract far-right] OR [Abstract neo-nazi*] OR [Abstract communis*] OR [Abstract nationalis*] OR [Abstract supremacist] OR [Abstract anarch*] OR [Abstract indoctrinat*]] AND [[Title family] OR [Title families] OR [Title familial*] OR [Title parent*] OR [Title siblin*] OR [Title brother*] OR [Title sister*] OR [Title father*] OR [Title mother*] OR [Title child*] OR [Title son*] OR [Title daughter*] OR [Title cousin*] OR [Title uncle*] OR [Title aunt*] OR [Title generation*] OR [Title maternal] OR [Title paternal] OR [Title grandparent*]] AND [[Keywords risk*] OR [Keywords protect*] OR [Keywords factor*] OR [Keywords correlat*] OR [Keywords relat*] OR [Keywords predict*] OR [Keywords caus*] OR [Keywords determina*] OR [Keywords consequenc*] OR [Keywords interven*] OR [Keywords evaluat*] OR [Keywords program*] OR [Keywords treat*] OR [Keywords prevent*] OR [Keywords experiment*] OR [Keywords "cross-section*"] OR [Keywords longitudinal*] OR [Keywords regress*]]

for [[Keywords radicali*] OR [Keywords terror*] OR [Keywords extremis*] OR [Keywords "lone wol*"] OR [Keywords lone-wol*] OR [Keywords "foreign fighter*"] OR [Keywords "single issue"] OR [Keywords jihad*] OR [Keywords islamis*] OR [Keywords salaf*] OR [Keywords left-wing] OR [Keywords far-left] OR [Keywords right-wing] OR [Keywords far-right] OR [Keywords neo-nazi*] OR [Keywords communis*] OR [Keywords nationalis*] OR [Keywords supremacist] OR [Keywords anarch*] OR [Keywords indoctrinat*]] AND [[Abstract family] OR [Abstract families] OR [Abstract familial*] OR [Abstract parent*] OR [Abstract siblin*] OR [Abstract brother*] OR [Abstract sister*] OR [Abstract father*] OR [Abstract mother*] OR [Abstract child*] OR [Abstract son*] OR [Abstract daughter*] OR [Abstract cousin*] OR [Abstract uncle*] OR [Abstract aunt*] OR [Abstract generation*] OR [Abstract maternal] OR [Abstract paternal] OR [Abstract grandparent*]] AND [[Title risk*] OR [Title protect*] OR [Title factor*] OR [Title correlat*] OR [Title relat*] OR [Title predict*] OR [Title caus*] OR [Title determina*] OR [Title consequenc*] OR [Title interven*] OR [Title evaluat*] OR [Title program*] OR [Title treat*] OR [Title prevent*] OR [Title experiment*] OR [Title "cross-section*"] OR [Title longitudinal*] OR [Title regress*]]

for [[Keywords radicali*] OR [Keywords terror*] OR [Keywords extremis*] OR [Keywords "lone wol*"] OR [Keywords lone-wol*] OR [Keywords "foreign fighter*"] OR [Keywords "single issue"] OR [Keywords jihad*] OR [Keywords islamis*] OR [Keywords salaf*] OR [Keywords left-wing] OR [Keywords far-left] OR [Keywords right-wing] OR [Keywords far-right] OR [Keywords neo-nazi*] OR [Keywords communis*] OR [Keywords nationalis*] OR [Keywords supremacist] OR [Keywords anarch*] OR [Keywords indoctrinat*]] AND [[Title family] OR [Title families] OR [Title familial*] OR [Title parent*] OR [Title siblin*] OR [Title brother*] OR [Title sister*] OR [Title father*] OR [Title mother*] OR [Title child*] OR [Title son*] OR [Title daughter*] OR [Title cousin*] OR [Title uncle*] OR [Title aunt*] OR [Title generation*] OR [Title maternal] OR [Title paternal] OR [Title grandparent*]] AND [[Abstract risk*] OR [Abstract protect*] OR [Abstract factor*] OR [Abstract correlat*] OR [Abstract relat*] OR [Abstract predict*] OR [Abstract caus*] OR [Abstract determina*] OR [Abstract consequenc*] OR [Abstract interven*] OR [Abstract evaluat*] OR [Abstract program*] OR [Abstract treat*] OR [Abstract prevent*] OR [Abstract experiment*] OR [Abstract "cross-section*"] OR [Abstract longitudinal*] OR [Abstract regress*]]

for [[Abstract radicali*] OR [Abstract terror*] OR [Abstract extremis*] OR [Abstract "lone wol*"] OR [Abstract lone-wol*] OR [Abstract "foreign fighter*"] OR [Abstract "single issue"] OR [Abstract jihad*] OR [Abstract islamis*] OR [Abstract salaf*] OR [Abstract left-wing] OR [Abstract far-left] OR [Abstract right-wing] OR [Abstract far-right] OR [Abstract neo-nazi*] OR [Abstract communis*] OR [Abstract nationalis*] OR [Abstract supremacist] OR [Abstract anarch*] OR [Abstract indoctrinat*]] AND [[Keywords family] OR [Keywords families] OR [Keywords familial*] OR [Keywords parent*] OR [Keywords siblin*] OR [Keywords brother*] OR [Keywords sister*] OR [Keywords father*] OR [Keywords mother*] OR [Keywords child*] OR [Keywords son*] OR [Keywords daughter*] OR [Keywords cousin*] OR [Keywords uncle*] OR [Keywords aunt*] OR [Keywords generation*] OR [Keywords maternal] OR [Keywords paternal] OR [Keywords grandparent*]] AND [[Title risk*] OR [Title protect*] OR [Title factor*] OR [Title correlat*] OR [Title relat*] OR [Title predict*] OR [Title caus*] OR [Title determina*] OR [Title consequenc*] OR [Title interven*] OR [Title evaluat*] OR [Title program*] OR [Title treat*] OR [Title prevent*] OR [Title experiment*] OR [Title "cross-section*"] OR [Title longitudinal*] OR [Title regress*]]

for [[Abstract radicali*] OR [Abstract terror*] OR [Abstract extremis*] OR [Abstract "lone wol*"] OR [Abstract lone-wol*] OR [Abstract "foreign fighter*"] OR [Abstract "single issue"] OR [Abstract jihad*] OR [Abstract islamis*] OR [Abstract salaf*] OR [Abstract left-wing] OR [Abstract far-left] OR [Abstract right-wing] OR [Abstract far-right] OR [Abstract neo-nazi*] OR [Abstract communis*] OR [Abstract nationalis*] OR [Abstract supremacist] OR [Abstract anarch*] OR [Abstract indoctrinat*]] AND [[Title family] OR [Title families] OR [Title familial*] OR [Title parent*] OR [Title siblin*] OR [Title brother*] OR [Title sister*] OR [Title father*] OR [Title mother*] OR [Title child*] OR [Title son*] OR [Title daughter*] OR [Title cousin*] OR [Title uncle*] OR [Title aunt*] OR [Title generation*] OR [Title maternal] OR [Title paternal] OR [Title grandparent*]] AND [[Title risk*] OR [Title protect*] OR [Title factor*] OR [Title correlat*] OR [Title relat*] OR [Title predict*] OR [Title caus*] OR [Title determina*] OR [Title consequenc*] OR [Title interven*] OR [Title evaluat*] OR [Title program*] OR [Title treat*] OR [Title prevent*] OR [Title experiment*] OR [Title "cross-section*"] OR [Title longitudinal*] OR [Title regress*]]

for [[Title radicali*] OR [Title terror*] OR [Title extremis*] OR [Title "lone wol*"] OR [Title lone-wol*] OR [Title "foreign fighter*"] OR [Title "single issue"] OR [Title jihad*] OR [Title islamis*] OR [Title salaf*] OR [Title left-wing] OR [Title far-left] OR [Title right-wing] OR [Title far-right] OR [Title neo-nazi*] OR [Title communis*] OR [Title nationalis*] OR [Title supremacist] OR [Title anarch*] OR [Title indoctrinat*]] AND [[Abstract family] OR [Abstract families] OR [Abstract familial*] OR [Abstract parent*] OR [Abstract siblin*] OR [Abstract brother*] OR [Abstract sister*] OR [Abstract father*] OR [Abstract mother*] OR [Abstract child*] OR [Abstract son*] OR [Abstract daughter*] OR [Abstract cousin*] OR [Abstract uncle*] OR [Abstract aunt*] OR [Abstract generation*] OR [Abstract maternal] OR [Abstract paternal] OR [Abstract grandparent*]] AND [[Title risk*] OR [Title protect*] OR [Title factor*] OR [Title correlat*] OR [Title relat*] OR [Title predict*] OR [Title caus*] OR [Title determina*] OR [Title consequenc*] OR [Title interven*] OR [Title evaluat*] OR [Title program*] OR [Title treat*] OR [Title prevent*] OR [Title experiment*] OR [Title "cross-section*"] OR [Title longitudinal*] OR [Title regress*]]

**ScienceDirect (1823-current)**

July 3, 2021

Searches conducted in “Title, abstract or author-specified keywords”. This database allows up to eight Boolean connectors per field.

| Radicalization And (family OR families OR familial OR parent OR sibling OR brother OR sister OR father OR mother) |
| --- |
| Radicalization And (child OR son OR daughter OR cousin OR uncle OR aunt OR generation OR maternal OR paternal) |
| Radicalization And grandparent |
| Radicalisation And (family OR families OR familial OR parent OR sibling OR brother OR sister OR father OR mother) |
| Radicalisation And (child OR son OR daughter OR cousin OR uncle OR aunt OR generation OR maternal OR paternal) |
| Radicalisation And grandparent |
| Terrorist And (family OR families OR familial OR parent OR sibling OR brother OR sister OR father OR mother) |
| Terrorist And (child OR son OR daughter OR cousin OR uncle OR aunt OR generation OR maternal OR paternal) |
| Terrorist And grandparent |
| Terrorism And (family OR families OR familial OR parent OR sibling OR brother OR sister OR father OR mother) |
| Terrorism And (child OR son OR daughter OR cousin OR uncle OR aunt OR generation OR maternal OR paternal) |
| Terrorism And grandparent |
| Extremist And (family OR families OR familial OR parent OR sibling OR brother OR sister OR father OR mother) |
| Extremist And (child OR son OR daughter OR cousin OR uncle OR aunt OR generation OR maternal OR paternal) |
| Extremist And grandparent |
| Extremism And (family OR families OR familial OR parent OR sibling OR brother OR sister OR father OR mother) |
| Extremism And (child OR son OR daughter OR cousin OR uncle OR aunt OR generation OR maternal OR paternal) |
| Extremism And grandparent |
| Lone-wolf And (family OR families OR familial OR parent OR sibling OR brother OR sister OR father OR mother) |
| Lone-wolf And (child OR son OR daughter OR cousin OR uncle OR aunt OR generation OR maternal OR paternal) |
| Lone-wolf And grandparent |
| Lone-wolves And (family OR families OR familial OR parent OR sibling OR brother OR sister OR father OR mother) |
| Lone-wolves And (child OR son OR daughter OR cousin OR uncle OR aunt OR generation OR maternal OR paternal) |
| Lone-wolves And grandparent |
| “Foreign fighter” And (family OR families OR familial OR parent OR sibling OR brother OR sister OR father OR mother) |
| “Foreign fighter” And (child OR son OR daughter OR cousin OR uncle OR aunt OR generation OR maternal OR paternal) |
| “Foreign fighter” And grandparent |
| “Single issue” And (family OR families OR familial OR parent OR sibling OR brother OR sister OR father OR mother) |
| “Single issue” And (child OR son OR daughter OR cousin OR uncle OR aunt OR generation OR maternal OR paternal) |
| “Single issue” And grandparent |
| Jihad And (family OR families OR familial OR parent OR sibling OR brother OR sister OR father OR mother) |
| Jihad And (child OR son OR daughter OR cousin OR uncle OR aunt OR generation OR maternal OR paternal) |
| Jihad And grandparent |
| Islamist And (family OR families OR familial OR parent OR sibling OR brother OR sister OR father OR mother) |
| Islamist And (child OR son OR daughter OR cousin OR uncle OR aunt OR generation OR maternal OR paternal) |
| Islamist And grandparent |
| Salafi And (family OR families OR familial OR parent OR sibling OR brother OR sister OR father OR mother) |
| Salafi And (child OR son OR daughter OR cousin OR uncle OR aunt OR generation OR maternal OR paternal) |
| Salafi And grandparent |
| Left-wing And (family OR families OR familial OR parent OR sibling OR brother OR sister OR father OR mother) |
| Left-wing And (child OR son OR daughter OR cousin OR uncle OR aunt OR generation OR maternal OR paternal) |
| Left-wing And grandparent |
| Right-wing And (family OR families OR familial OR parent OR sibling OR brother OR sister OR father OR mother) |
| Right-wing And (child OR son OR daughter OR cousin OR uncle OR aunt OR generation OR maternal OR paternal) |
| Right-wing And grandparent |
| Far-left And (family OR families OR familial OR parent OR sibling OR brother OR sister OR father OR mother) |
| Far-left And (child OR son OR daughter OR cousin OR uncle OR aunt OR generation OR maternal OR paternal) |
| Far-left And grandparent |
| Far-right And (family OR families OR familial OR parent OR sibling OR brother OR sister OR father OR mother) |
| Far-right And (child OR son OR daughter OR cousin OR uncle OR aunt OR generation OR maternal OR paternal) |
| Far-right And grandparent |
| Neo-nazi And (family OR families OR familial OR parent OR sibling OR brother OR sister OR father OR mother) |
| Neo-nazi And (child OR son OR daughter OR cousin OR uncle OR aunt OR generation OR maternal OR paternal) |
| Neo-nazi And grandparent |
| communist And (family OR families OR familial OR parent OR sibling OR brother OR sister OR father OR mother) |
| communist And (child OR son OR daughter OR cousin OR uncle OR aunt OR generation OR maternal OR paternal) |
| communist And grandparent |
| Nationalist And (family OR families OR familial OR parent OR sibling OR brother OR sister OR father OR mother) |
| Nationalist And (child OR son OR daughter OR cousin OR uncle OR aunt OR generation OR maternal OR paternal) |
| Nationalist And grandparent |
| Supremacist And (family OR families OR familial OR parent OR sibling OR brother OR sister OR father OR mother) |
| Supremacist And (child OR son OR daughter OR cousin OR uncle OR aunt OR generation OR maternal OR paternal) |
| Supremacist And grandparent |
| Anarchist And (family OR families OR familial OR parent OR sibling OR brother OR sister OR father OR mother) |
| Anarchist And (child OR son OR daughter OR cousin OR uncle OR aunt OR generation OR maternal OR paternal) |
| Anarchist And grandparent |
| Indoctrination And (family OR families OR familial OR parent OR sibling OR brother OR sister OR father OR mother) |
| Indoctrination And (child OR son OR daughter OR cousin OR uncle OR aunt OR generation OR maternal OR paternal) |
| Indoctrination And grandparent |

**SCOPUS**

**(1841-current)**

June 26, 2021

( TITLE-ABS-KEY ( radicali* OR terror* OR extremis* OR ( “lone AND wol*” ) OR lone-wol* OR ( “foreign AND fighter*” ) OR ( “single AND issue” ) OR jihad* OR islamis* OR salaf* OR left-wing OR far-left OR right-wing OR far-right OR neo-nazi* OR communis* OR nationalis* OR supremacist OR anarch* OR indoctrinat* ) AND TITLE-ABS-KEY ( family OR families OR familial* OR *parent* OR siblin* OR brother* OR sister* OR father* OR mother* OR child* OR son* OR daughter* OR cousin* OR uncle* OR aunt* OR generation* OR maternal OR paternal OR grandparent ) AND TITLE-ABS-KEY ( risk* OR protect* OR factor OR correlat* OR relat* OR predict* OR caus* OR determina* OR consequenc* OR intervent* OR evaluat* OR program* OR treat* OR prevent* OR experiment* OR “cross-section*” OR longitudinal* OR regress* ) ) : 16,914

**Taylor & Francis Online**

**(1798- current)**

July 7-10, 2021

Your search for [[Abstract: radicali*] OR [Abstract: terror*] OR [Abstract: extremis*] OR [Abstract: "lone wol*"] OR [Abstract: lone-wol*] OR [Abstract: "foreign fighter*"] OR [Abstract: "single issue"] OR [Abstract: jihad*] OR [Abstract: islamis*] OR [Abstract: salaf*] OR [Abstract: left-wing] OR [Abstract: far-left] OR [Abstract: right-wing] OR [Abstract: far-right] OR [Abstract: neo-nazi*] OR [Abstract: communis*] OR [Abstract: nationalis*] OR [Abstract: supremacist] OR [Abstract: anarch*] OR [Abstract: indoctrinat*]] AND [[Abstract: family] OR [Abstract: families] OR [Abstract: familial*] OR [Abstract: parent*] OR [Abstract: siblin*] OR [Abstract: brother*] OR [Abstract: sister*] OR [Abstract: father*] OR [Abstract: mother*] OR [Abstract: child*] OR [Abstract: son*] OR [Abstract: daughter*] OR [Abstract: cousin*] OR [Abstract: uncle*] OR [Abstract: aunt*] OR [Abstract: generation*] OR [Abstract: maternal] OR [Abstract: paternal] OR [Abstract: grandparent*]] AND [[Abstract: risk*] OR [Abstract: protect*] OR [Abstract: factor*] OR [Abstract: correlat*] OR [Abstract: relat*] OR [Abstract: predict*] OR [Abstract: caus*] OR [Abstract: determina*] OR [Abstract: consequenc*] OR [Abstract: interven*] OR [Abstract: evaluat*] OR [Abstract: program*] OR [Abstract: treat*] OR [Abstract: prevent*] OR [Abstract: experiment*] OR [Abstract: "cross-section*"] OR [Abstract: longitudinal*] OR [Abstract: regress*]]

[[Abstract: radicali*] OR [Abstract: terror*] OR [Abstract: extremis*] OR [Abstract: "lone wol*"] OR [Abstract: lone-wol*] OR [Abstract: "foreign fighter*"] OR [Abstract: "single issue"] OR [Abstract: jihad*] OR [Abstract: islamis*] OR [Abstract: salaf*] OR [Abstract: left-wing] OR [Abstract: far-left] OR [Abstract: right-wing] OR [Abstract: far-right] OR [Abstract: neo-nazi*] OR [Abstract: communis*] OR [Abstract: nationalis*] OR [Abstract: supremacist] OR [Abstract: anarch*] OR [Abstract: indoctrinat*]] AND [[Abstract: family] OR [Abstract: families] OR [Abstract: familial*] OR [Abstract: parent*] OR [Abstract: siblin*] OR [Abstract: brother*] OR [Abstract: sister*] OR [Abstract: father*] OR [Abstract: mother*] OR [Abstract: child*] OR [Abstract: son*] OR [Abstract: daughter*] OR [Abstract: cousin*] OR [Abstract: uncle*] OR [Abstract: aunt*] OR [Abstract: generation*] OR [Abstract: maternal] OR [Abstract: paternal] OR [Abstract: grandparent*]] AND [[Keywords: risk*] OR [Keywords: protect*] OR [Keywords: factor*] OR [Keywords: correlat*] OR [Keywords: relat*] OR [Keywords: predict*] OR [Keywords: caus*] OR [Keywords: determina*] OR [Keywords: consequenc*] OR [Keywords: interven*] OR [Keywords: evaluat*] OR [Keywords: program*] OR [Keywords: treat*] OR [Keywords: prevent*] OR [Keywords: experiment*] OR [Keywords: "cross-section*"] OR [Keywords: longitudinal*] OR [Keywords: regress*]]

Your search for [[Abstract: radicali*] OR [Abstract: terror*] OR [Abstract: extremis*] OR [Abstract: "lone wol*"] OR [Abstract: lone-wol*] OR [Abstract: "foreign fighter*"] OR [Abstract: "single issue"] OR [Abstract: jihad*] OR [Abstract: islamis*] OR [Abstract: salaf*] OR [Abstract: left-wing] OR [Abstract: far-left] OR [Abstract: right-wing] OR [Abstract: far-right] OR [Abstract: neo-nazi*] OR [Abstract: communis*] OR [Abstract: nationalis*] OR [Abstract: supremacist] OR [Abstract: anarch*] OR [Abstract: indoctrinat*]] AND [[Keywords: family] OR [Keywords: families] OR [Keywords: familial*] OR [Keywords: parent*] OR [Keywords: siblin*] OR [Keywords: brother*] OR [Keywords: sister*] OR [Keywords: father*] OR [Keywords: mother*] OR [Keywords: child*] OR [Keywords: son*] OR [Keywords: daughter*] OR [Keywords: cousin*] OR [Keywords: uncle*] OR [Keywords: aunt*] OR [Keywords: generation*] OR [Keywords: maternal] OR [Keywords: paternal] OR [Keywords: grandparent*]] AND [[Abstract: risk*] OR [Abstract: protect*] OR [Abstract: factor*] OR [Abstract: correlat*] OR [Abstract: relat*] OR [Abstract: predict*] OR [Abstract: caus*] OR [Abstract: determina*] OR [Abstract: consequenc*] OR [Abstract: interven*] OR [Abstract: evaluat*] OR [Abstract: program*] OR [Abstract: treat*] OR [Abstract: prevent*] OR [Abstract: experiment*] OR [Abstract: "cross-section*"] OR [Abstract: longitudinal*] OR [Abstract: regress*]]

Your search for [[Abstract: radicali*] OR [Abstract: terror*] OR [Abstract: extremis*] OR [Abstract: "lone wol*"] OR [Abstract: lone-wol*] OR [Abstract: "foreign fighter*"] OR [Abstract: "single issue"] OR [Abstract: jihad*] OR [Abstract: islamis*] OR [Abstract: salaf*] OR [Abstract: left-wing] OR [Abstract: far-left] OR [Abstract: right-wing] OR [Abstract: far-right] OR [Abstract: neo-nazi*] OR [Abstract: communis*] OR [Abstract: nationalis*] OR [Abstract: supremacist] OR [Abstract: anarch*] OR [Abstract: indoctrinat*]] AND [[Abstract: family] OR [Abstract: families] OR [Abstract: familial*] OR [Abstract: parent*] OR [Abstract: siblin*] OR [Abstract: brother*] OR [Abstract: sister*] OR [Abstract: father*] OR [Abstract: mother*] OR [Abstract: child*] OR [Abstract: son*] OR [Abstract: daughter*] OR [Abstract: cousin*] OR [Abstract: uncle*] OR [Abstract: aunt*] OR [Abstract: generation*] OR [Abstract: maternal] OR [Abstract: paternal] OR [Abstract: grandparent*]] AND [[Publication Title: risk*] OR [Publication Title: protect*] OR [Publication Title: factor*] OR [Publication Title: correlat*] OR [Publication Title: relat*] OR [Publication Title: predict*] OR [Publication Title: caus*] OR [Publication Title: determina*] OR [Publication Title: consequenc*] OR [Publication Title: interven*] OR [Publication Title: evaluat*] OR [Publication Title: program*] OR [Publication Title: treat*] OR [Publication Title: prevent*] OR [Publication Title: experiment*] OR [Publication Title: "cross-section*"] OR [Publication Title: longitudinal*] OR [Publication Title: regress*]]

Your search for [[Abstract: radicali*] OR [Abstract: terror*] OR [Abstract: extremis*] OR [Abstract: "lone wol*"] OR [Abstract: lone-wol*] OR [Abstract: "foreign fighter*"] OR [Abstract: "single issue"] OR [Abstract: jihad*] OR [Abstract: islamis*] OR [Abstract: salaf*] OR [Abstract: left-wing] OR [Abstract: far-left] OR [Abstract: right-wing] OR [Abstract: far-right] OR [Abstract: neo-nazi*] OR [Abstract: communis*] OR [Abstract: nationalis*] OR [Abstract: supremacist] OR [Abstract: anarch*] OR [Abstract: indoctrinat*]] AND [[Keywords: family] OR [Keywords: families] OR [Keywords: familial*] OR [Keywords: parent*] OR [Keywords: siblin*] OR [Keywords: brother*] OR [Keywords: sister*] OR [Keywords: father*] OR [Keywords: mother*] OR [Keywords: child*] OR [Keywords: son*] OR [Keywords: daughter*] OR [Keywords: cousin*] OR [Keywords: uncle*] OR [Keywords: aunt*] OR [Keywords: generation*] OR [Keywords: maternal] OR [Keywords: paternal] OR [Keywords: grandparent*]] AND [[Keywords: risk*] OR [Keywords: protect*] OR [Keywords: factor*] OR [Keywords: correlat*] OR [Keywords: relat*] OR [Keywords: predict*] OR [Keywords: caus*] OR [Keywords: determina*] OR [Keywords: consequenc*] OR [Keywords: interven*] OR [Keywords: evaluat*] OR [Keywords: program*] OR [Keywords: treat*] OR [Keywords: prevent*] OR [Keywords: experiment*] OR [Keywords: "cross-section*"] OR [Keywords: longitudinal*] OR [Keywords: regress*]]

Your search for [[Abstract: radicali*] OR [Abstract: terror*] OR [Abstract: extremis*] OR [Abstract: "lone wol*"] OR [Abstract: lone-wol*] OR [Abstract: "foreign fighter*"] OR [Abstract: "single issue"] OR [Abstract: jihad*] OR [Abstract: islamis*] OR [Abstract: salaf*] OR [Abstract: left-wing] OR [Abstract: far-left] OR [Abstract: right-wing] OR [Abstract: far-right] OR [Abstract: neo-nazi*] OR [Abstract: communis*] OR [Abstract: nationalis*] OR [Abstract: supremacist] OR [Abstract: anarch*] OR [Abstract: indoctrinat*]] AND [[Keywords: family] OR [Keywords: families] OR [Keywords: familial*] OR [Keywords: parent*] OR [Keywords: siblin*] OR [Keywords: brother*] OR [Keywords: sister*] OR [Keywords: father*] OR [Keywords: mother*] OR [Keywords: child*] OR [Keywords: son*] OR [Keywords: daughter*] OR [Keywords: cousin*] OR [Keywords: uncle*] OR [Keywords: aunt*] OR [Keywords: generation*] OR [Keywords: maternal] OR [Keywords: paternal] OR [Keywords: grandparent*]] AND [[Publication Title: risk*] OR [Publication Title: protect*] OR [Publication Title: factor*] OR [Publication Title: correlat*] OR [Publication Title: relat*] OR [Publication Title: predict*] OR [Publication Title: caus*] OR [Publication Title: determina*] OR [Publication Title: consequenc*] OR [Publication Title: interven*] OR [Publication Title: evaluat*] OR [Publication Title: program*] OR [Publication Title: treat*] OR [Publication Title: prevent*] OR [Publication Title: experiment*] OR [Publication Title: "cross-section*"] OR [Publication Title: longitudinal*] OR [Publication Title: regress*]]

Your search for [[Abstract: radicali*] OR [Abstract: terror*] OR [Abstract: extremis*] OR [Abstract: "lone wol*"] OR [Abstract: lone-wol*] OR [Abstract: "foreign fighter*"] OR [Abstract: "single issue"] OR [Abstract: jihad*] OR [Abstract: islamis*] OR [Abstract: salaf*] OR [Abstract: left-wing] OR [Abstract: far-left] OR [Abstract: right-wing] OR [Abstract: far-right] OR [Abstract: neo-nazi*] OR [Abstract: communis*] OR [Abstract: nationalis*] OR [Abstract: supremacist] OR [Abstract: anarch*] OR [Abstract: indoctrinat*]] AND [[Publication Title: family] OR [Publication Title: families] OR [Publication Title: familial*] OR [Publication Title: parent*] OR [Publication Title: siblin*] OR [Publication Title: brother*] OR [Publication Title: sister*] OR [Publication Title: father*] OR [Publication Title: mother*] OR [Publication Title: child*] OR [Publication Title: son*] OR [Publication Title: daughter*] OR [Publication Title: cousin*] OR [Publication Title: uncle*] OR [Publication Title: aunt*] OR [Publication Title: generation*] OR [Publication Title: maternal] OR [Publication Title: paternal] OR [Publication Title: grandparent*]] AND [[Keywords: risk*] OR [Keywords: protect*] OR [Keywords: factor*] OR [Keywords: correlat*] OR [Keywords: relat*] OR [Keywords: predict*] OR [Keywords: caus*] OR [Keywords: determina*] OR [Keywords: consequenc*] OR [Keywords: interven*] OR [Keywords: evaluat*] OR [Keywords: program*] OR [Keywords: treat*] OR [Keywords: prevent*] OR [Keywords: experiment*] OR [Keywords: "cross-section*"] OR [Keywords: longitudinal*] OR [Keywords: regress*]]

Your search for [[Abstract: radicali*] OR [Abstract: terror*] OR [Abstract: extremis*] OR [Abstract: "lone wol*"] OR [Abstract: lone-wol*] OR [Abstract: "foreign fighter*"] OR [Abstract: "single issue"] OR [Abstract: jihad*] OR [Abstract: islamis*] OR [Abstract: salaf*] OR [Abstract: left-wing] OR [Abstract: far-left] OR [Abstract: right-wing] OR [Abstract: far-right] OR [Abstract: neo-nazi*] OR [Abstract: communis*] OR [Abstract: nationalis*] OR [Abstract: supremacist] OR [Abstract: anarch*] OR [Abstract: indoctrinat*]] AND [[Publication Title: family] OR [Publication Title: families] OR [Publication Title: familial*] OR [Publication Title: parent*] OR [Publication Title: siblin*] OR [Publication Title: brother*] OR [Publication Title: sister*] OR [Publication Title: father*] OR [Publication Title: mother*] OR [Publication Title: child*] OR [Publication Title: son*] OR [Publication Title: daughter*] OR [Publication Title: cousin*] OR [Publication Title: uncle*] OR [Publication Title: aunt*] OR [Publication Title: generation*] OR [Publication Title: maternal] OR [Publication Title: paternal] OR [Publication Title: grandparent*]] AND [[Abstract: risk*] OR [Abstract: protect*] OR [Abstract: factor*] OR [Abstract: correlat*] OR [Abstract: relat*] OR [Abstract: predict*] OR [Abstract: caus*] OR [Abstract: determina*] OR [Abstract: consequenc*] OR [Abstract: interven*] OR [Abstract: evaluat*] OR [Abstract: program*] OR [Abstract: treat*] OR [Abstract: prevent*] OR [Abstract: experiment*] OR [Abstract: "cross-section*"] OR [Abstract: longitudinal*] OR [Abstract: regress*]]

Your search for [[Keywords: radicali*] OR [Keywords: terror*] OR [Keywords: extremis*] OR [Keywords: "lone wol*"] OR [Keywords: lone-wol*] OR [Keywords: "foreign fighter*"] OR [Keywords: "single issue"] OR [Keywords: jihad*] OR [Keywords: islamis*] OR [Keywords: salaf*] OR [Keywords: left-wing] OR [Keywords: far-left] OR [Keywords: right-wing] OR [Keywords: far-right] OR [Keywords: neo-nazi*] OR [Keywords: communis*] OR [Keywords: nationalis*] OR [Keywords: supremacist] OR [Keywords: anarch*] OR [Keywords: indoctrinat*]] AND [[Abstract: family] OR [Abstract: families] OR [Abstract: familial*] OR [Abstract: parent*] OR [Abstract: siblin*] OR [Abstract: brother*] OR [Abstract: sister*] OR [Abstract: father*] OR [Abstract: mother*] OR [Abstract: child*] OR [Abstract: son*] OR [Abstract: daughter*] OR [Abstract: cousin*] OR [Abstract: uncle*] OR [Abstract: aunt*] OR [Abstract: generation*] OR [Abstract: maternal] OR [Abstract: paternal] OR [Abstract: grandparent*]] AND [[Keywords: risk*] OR [Keywords: protect*] OR [Keywords: factor*] OR [Keywords: correlat*] OR [Keywords: relat*] OR [Keywords: predict*] OR [Keywords: caus*] OR [Keywords: determina*] OR [Keywords: consequenc*] OR [Keywords: interven*] OR [Keywords: evaluat*] OR [Keywords: program*] OR [Keywords: treat*] OR [Keywords: prevent*] OR [Keywords: experiment*] OR [Keywords: "cross-section*"] OR [Keywords: longitudinal*] OR [Keywords: regress*]]

Your search for [[Keywords: radicali*] OR [Keywords: terror*] OR [Keywords: extremis*] OR [Keywords: "lone wol*"] OR [Keywords: lone-wol*] OR [Keywords: "foreign fighter*"] OR [Keywords: "single issue"] OR [Keywords: jihad*] OR [Keywords: islamis*] OR [Keywords: salaf*] OR [Keywords: left-wing] OR [Keywords: far-left] OR [Keywords: right-wing] OR [Keywords: far-right] OR [Keywords: neo-nazi*] OR [Keywords: communis*] OR [Keywords: nationalis*] OR [Keywords: supremacist] OR [Keywords: anarch*] OR [Keywords: indoctrinat*]] AND [[Abstract: family] OR [Abstract: families] OR [Abstract: familial*] OR [Abstract: parent*] OR [Abstract: siblin*] OR [Abstract: brother*] OR [Abstract: sister*] OR [Abstract: father*] OR [Abstract: mother*] OR [Abstract: child*] OR [Abstract: son*] OR [Abstract: daughter*] OR [Abstract: cousin*] OR [Abstract: uncle*] OR [Abstract: aunt*] OR [Abstract: generation*] OR [Abstract: maternal] OR [Abstract: paternal] OR [Abstract: grandparent*]] AND [[Abstract: risk*] OR [Abstract: protect*] OR [Abstract: factor*] OR [Abstract: correlat*] OR [Abstract: relat*] OR [Abstract: predict*] OR [Abstract: caus*] OR [Abstract: determina*] OR [Abstract: consequenc*] OR [Abstract: interven*] OR [Abstract: evaluat*] OR [Abstract: program*] OR [Abstract: treat*] OR [Abstract: prevent*] OR [Abstract: experiment*] OR [Abstract: "cross-section*"] OR [Abstract: longitudinal*] OR [Abstract: regress*]]

Your search for [[Abstract: radicali*] OR [Abstract: terror*] OR [Abstract: extremis*] OR [Abstract: "lone wol*"] OR [Abstract: lone-wol*] OR [Abstract: "foreign fighter*"] OR [Abstract: "single issue"] OR [Abstract: jihad*] OR [Abstract: islamis*] OR [Abstract: salaf*] OR [Abstract: left-wing] OR [Abstract: far-left] OR [Abstract: right-wing] OR [Abstract: far-right] OR [Abstract: neo-nazi*] OR [Abstract: communis*] OR [Abstract: nationalis*] OR [Abstract: supremacist] OR [Abstract: anarch*] OR [Abstract: indoctrinat*]] AND [[Publication Title: family] OR [Publication Title: families] OR [Publication Title: familial*] OR [Publication Title: parent*] OR [Publication Title: siblin*] OR [Publication Title: brother*] OR [Publication Title: sister*] OR [Publication Title: father*] OR [Publication Title: mother*] OR [Publication Title: child*] OR [Publication Title: son*] OR [Publication Title: daughter*] OR [Publication Title: cousin*] OR [Publication Title: uncle*] OR [Publication Title: aunt*] OR [Publication Title: generation*] OR [Publication Title: maternal] OR [Publication Title: paternal] OR [Publication Title: grandparent*]] AND [[Publication Title: risk*] OR [Publication Title: protect*] OR [Publication Title: factor*] OR [Publication Title: correlat*] OR [Publication Title: relat*] OR [Publication Title: predict*] OR [Publication Title: caus*] OR [Publication Title: determina*] OR [Publication Title: consequenc*] OR [Publication Title: interven*] OR [Publication Title: evaluat*] OR [Publication Title: program*] OR [Publication Title: treat*] OR [Publication Title: prevent*] OR [Publication Title: experiment*] OR [Publication Title: "cross-section*"] OR [Publication Title: longitudinal*] OR [Publication Title: regress*]]

Your search for [[Keywords: radicali*] OR [Keywords: terror*] OR [Keywords: extremis*] OR [Keywords: "lone wol*"] OR [Keywords: lone-wol*] OR [Keywords: "foreign fighter*"] OR [Keywords: "single issue"] OR [Keywords: jihad*] OR [Keywords: islamis*] OR [Keywords: salaf*] OR [Keywords: left-wing] OR [Keywords: far-left] OR [Keywords: right-wing] OR [Keywords: far-right] OR [Keywords: neo-nazi*] OR [Keywords: communis*] OR [Keywords: nationalis*] OR [Keywords: supremacist] OR [Keywords: anarch*] OR [Keywords: indoctrinat*]] AND [[Abstract: family] OR [Abstract: families] OR [Abstract: familial*] OR [Abstract: parent*] OR [Abstract: siblin*] OR [Abstract: brother*] OR [Abstract: sister*] OR [Abstract: father*] OR [Abstract: mother*] OR [Abstract: child*] OR [Abstract: son*] OR [Abstract: daughter*] OR [Abstract: cousin*] OR [Abstract: uncle*] OR [Abstract: aunt*] OR [Abstract: generation*] OR [Abstract: maternal] OR [Abstract: paternal] OR [Abstract: grandparent*]] AND [[Publication Title: risk*] OR [Publication Title: protect*] OR [Publication Title: factor*] OR [Publication Title: correlat*] OR [Publication Title: relat*] OR [Publication Title: predict*] OR [Publication Title: caus*] OR [Publication Title: determina*] OR [Publication Title: consequenc*] OR [Publication Title: interven*] OR [Publication Title: evaluat*] OR [Publication Title: program*] OR [Publication Title: treat*] OR [Publication Title: prevent*] OR [Publication Title: experiment*] OR [Publication Title: "cross-section*"] OR [Publication Title: longitudinal*] OR [Publication Title: regress*]]

Your search for [[Keywords: radicali*] OR [Keywords: terror*] OR [Keywords: extremis*] OR [Keywords: "lone wol*"] OR [Keywords: lone-wol*] OR [Keywords: "foreign fighter*"] OR [Keywords: "single issue"] OR [Keywords: jihad*] OR [Keywords: islamis*] OR [Keywords: salaf*] OR [Keywords: left-wing] OR [Keywords: far-left] OR [Keywords: right-wing] OR [Keywords: far-right] OR [Keywords: neo-nazi*] OR [Keywords: communis*] OR [Keywords: nationalis*] OR [Keywords: supremacist] OR [Keywords: anarch*] OR [Keywords: indoctrinat*]] AND [[Keywords: family] OR [Keywords: families] OR [Keywords: familial*] OR [Keywords: parent*] OR [Keywords: siblin*] OR [Keywords: brother*] OR [Keywords: sister*] OR [Keywords: father*] OR [Keywords: mother*] OR [Keywords: child*] OR [Keywords: son*] OR [Keywords: daughter*] OR [Keywords: cousin*] OR [Keywords: uncle*] OR [Keywords: aunt*] OR [Keywords: generation*] OR [Keywords: maternal] OR [Keywords: paternal] OR [Keywords: grandparent*]] AND [[Abstract: risk*] OR [Abstract: protect*] OR [Abstract: factor*] OR [Abstract: correlat*] OR [Abstract: relat*] OR [Abstract: predict*] OR [Abstract: caus*] OR [Abstract: determina*] OR [Abstract: consequenc*] OR [Abstract: interven*] OR [Abstract: evaluat*] OR [Abstract: program*] OR [Abstract: treat*] OR [Abstract: prevent*] OR [Abstract: experiment*] OR [Abstract: "cross-section*"] OR [Abstract: longitudinal*] OR [Abstract: regress*]]

Your search for [[Keywords: radicali*] OR [Keywords: terror*] OR [Keywords: extremis*] OR [Keywords: "lone wol*"] OR [Keywords: lone-wol*] OR [Keywords: "foreign fighter*"] OR [Keywords: "single issue"] OR [Keywords: jihad*] OR [Keywords: islamis*] OR [Keywords: salaf*] OR [Keywords: left-wing] OR [Keywords: far-left] OR [Keywords: right-wing] OR [Keywords: far-right] OR [Keywords: neo-nazi*] OR [Keywords: communis*] OR [Keywords: nationalis*] OR [Keywords: supremacist] OR [Keywords: anarch*] OR [Keywords: indoctrinat*]] AND [[Keywords: family] OR [Keywords: families] OR [Keywords: familial*] OR [Keywords: parent*] OR [Keywords: siblin*] OR [Keywords: brother*] OR [Keywords: sister*] OR [Keywords: father*] OR [Keywords: mother*] OR [Keywords: child*] OR [Keywords: son*] OR [Keywords: daughter*] OR [Keywords: cousin*] OR [Keywords: uncle*] OR [Keywords: aunt*] OR [Keywords: generation*] OR [Keywords: maternal] OR [Keywords: paternal] OR [Keywords: grandparent*]] AND [[Keywords: risk*] OR [Keywords: protect*] OR [Keywords: factor*] OR [Keywords: correlat*] OR [Keywords: relat*] OR [Keywords: predict*] OR [Keywords: caus*] OR [Keywords: determina*] OR [Keywords: consequenc*] OR [Keywords: interven*] OR [Keywords: evaluat*] OR [Keywords: program*] OR [Keywords: treat*] OR [Keywords: prevent*] OR [Keywords: experiment*] OR [Keywords: "cross-section*"] OR [Keywords: longitudinal*] OR [Keywords: regress*]]

Your search for [[Keywords: radicali*] OR [Keywords: terror*] OR [Keywords: extremis*] OR [Keywords: "lone wol*"] OR [Keywords: lone-wol*] OR [Keywords: "foreign fighter*"] OR [Keywords: "single issue"] OR [Keywords: jihad*] OR [Keywords: islamis*] OR [Keywords: salaf*] OR [Keywords: left-wing] OR [Keywords: far-left] OR [Keywords: right-wing] OR [Keywords: far-right] OR [Keywords: neo-nazi*] OR [Keywords: communis*] OR [Keywords: nationalis*] OR [Keywords: supremacist] OR [Keywords: anarch*] OR [Keywords: indoctrinat*]] AND [[Keywords: family] OR [Keywords: families] OR [Keywords: familial*] OR [Keywords: parent*] OR [Keywords: siblin*] OR [Keywords: brother*] OR [Keywords: sister*] OR [Keywords: father*] OR [Keywords: mother*] OR [Keywords: child*] OR [Keywords: son*] OR [Keywords: daughter*] OR [Keywords: cousin*] OR [Keywords: uncle*] OR [Keywords: aunt*] OR [Keywords: generation*] OR [Keywords: maternal] OR [Keywords: paternal] OR [Keywords: grandparent*]] AND [[Publication Title: risk*] OR [Publication Title: protect*] OR [Publication Title: factor*] OR [Publication Title: correlat*] OR [Publication Title: relat*] OR [Publication Title: predict*] OR [Publication Title: caus*] OR [Publication Title: determina*] OR [Publication Title: consequenc*] OR [Publication Title: interven*] OR [Publication Title: evaluat*] OR [Publication Title: program*] OR [Publication Title: treat*] OR [Publication Title: prevent*] OR [Publication Title: experiment*] OR [Publication Title: "cross-section*"] OR [Publication Title: longitudinal*] OR [Publication Title: regress*]]

Your search for [[Keywords: radicali*] OR [Keywords: terror*] OR [Keywords: extremis*] OR [Keywords: "lone wol*"] OR [Keywords: lone-wol*] OR [Keywords: "foreign fighter*"] OR [Keywords: "single issue"] OR [Keywords: jihad*] OR [Keywords: islamis*] OR [Keywords: salaf*] OR [Keywords: left-wing] OR [Keywords: far-left] OR [Keywords: right-wing] OR [Keywords: far-right] OR [Keywords: neo-nazi*] OR [Keywords: communis*] OR [Keywords: nationalis*] OR [Keywords: supremacist] OR [Keywords: anarch*] OR [Keywords: indoctrinat*]] AND [[Publication Title: family] OR [Publication Title: families] OR [Publication Title: familial*] OR [Publication Title: parent*] OR [Publication Title: siblin*] OR [Publication Title: brother*] OR [Publication Title: sister*] OR [Publication Title: father*] OR [Publication Title: mother*] OR [Publication Title: child*] OR [Publication Title: son*] OR [Publication Title: daughter*] OR [Publication Title: cousin*] OR [Publication Title: uncle*] OR [Publication Title: aunt*] OR [Publication Title: generation*] OR [Publication Title: maternal] OR [Publication Title: paternal] OR [Publication Title: grandparent*]] AND [[Abstract: risk*] OR [Abstract: protect*] OR [Abstract: factor*] OR [Abstract: correlat*] OR [Abstract: relat*] OR [Abstract: predict*] OR [Abstract: caus*] OR [Abstract: determina*] OR [Abstract: consequenc*] OR [Abstract: interven*] OR [Abstract: evaluat*] OR [Abstract: program*] OR [Abstract: treat*] OR [Abstract: prevent*] OR [Abstract: experiment*] OR [Abstract: "cross-section*"] OR [Abstract: longitudinal*] OR [Abstract: regress*]]

Your search for [[Keywords: radicali*] OR [Keywords: terror*] OR [Keywords: extremis*] OR [Keywords: "lone wol*"] OR [Keywords: lone-wol*] OR [Keywords: "foreign fighter*"] OR [Keywords: "single issue"] OR [Keywords: jihad*] OR [Keywords: islamis*] OR [Keywords: salaf*] OR [Keywords: left-wing] OR [Keywords: far-left] OR [Keywords: right-wing] OR [Keywords: far-right] OR [Keywords: neo-nazi*] OR [Keywords: communis*] OR [Keywords: nationalis*] OR [Keywords: supremacist] OR [Keywords: anarch*] OR [Keywords: indoctrinat*]] AND [[Publication Title: family] OR [Publication Title: families] OR [Publication Title: familial*] OR [Publication Title: parent*] OR [Publication Title: siblin*] OR [Publication Title: brother*] OR [Publication Title: sister*] OR [Publication Title: father*] OR [Publication Title: mother*] OR [Publication Title: child*] OR [Publication Title: son*] OR [Publication Title: daughter*] OR [Publication Title: cousin*] OR [Publication Title: uncle*] OR [Publication Title: aunt*] OR [Publication Title: generation*] OR [Publication Title: maternal] OR [Publication Title: paternal] OR [Publication Title: grandparent*]] AND [[Keywords: risk*] OR [Keywords: protect*] OR [Keywords: factor*] OR [Keywords: correlat*] OR [Keywords: relat*] OR [Keywords: predict*] OR [Keywords: caus*] OR [Keywords: determina*] OR [Keywords: consequenc*] OR [Keywords: interven*] OR [Keywords: evaluat*] OR [Keywords: program*] OR [Keywords: treat*] OR [Keywords: prevent*] OR [Keywords: experiment*] OR [Keywords: "cross-section*"] OR [Keywords: longitudinal*] OR [Keywords: regress*]]

Your search for [[Keywords: radicali*] OR [Keywords: terror*] OR [Keywords: extremis*] OR [Keywords: "lone wol*"] OR [Keywords: lone-wol*] OR [Keywords: "foreign fighter*"] OR [Keywords: "single issue"] OR [Keywords: jihad*] OR [Keywords: islamis*] OR [Keywords: salaf*] OR [Keywords: left-wing] OR [Keywords: far-left] OR [Keywords: right-wing] OR [Keywords: far-right] OR [Keywords: neo-nazi*] OR [Keywords: communis*] OR [Keywords: nationalis*] OR [Keywords: supremacist] OR [Keywords: anarch*] OR [Keywords: indoctrinat*]] AND [[Publication Title: family] OR [Publication Title: families] OR [Publication Title: familial*] OR [Publication Title: parent*] OR [Publication Title: siblin*] OR [Publication Title: brother*] OR [Publication Title: sister*] OR [Publication Title: father*] OR [Publication Title: mother*] OR [Publication Title: child*] OR [Publication Title: son*] OR [Publication Title: daughter*] OR [Publication Title: cousin*] OR [Publication Title: uncle*] OR [Publication Title: aunt*] OR [Publication Title: generation*] OR [Publication Title: maternal] OR [Publication Title: paternal] OR [Publication Title: grandparent*]] AND [[Publication Title: risk*] OR [Publication Title: protect*] OR [Publication Title: factor*] OR [Publication Title: correlat*] OR [Publication Title: relat*] OR [Publication Title: predict*] OR [Publication Title: caus*] OR [Publication Title: determina*] OR [Publication Title: consequenc*] OR [Publication Title: interven*] OR [Publication Title: evaluat*] OR [Publication Title: program*] OR [Publication Title: treat*] OR [Publication Title: prevent*] OR [Publication Title: experiment*] OR [Publication Title: "cross-section*"] OR [Publication Title: longitudinal*] OR [Publication Title: regress*]]

Your search for [[Publication Title: radicali*] OR [Publication Title: terror*] OR [Publication Title: extremis*] OR [Publication Title: "lone wol*"] OR [Publication Title: lone-wol*] OR [Publication Title: "foreign fighter*"] OR [Publication Title: "single issue"] OR [Publication Title: jihad*] OR [Publication Title: islamis*] OR [Publication Title: salaf*] OR [Publication Title: left-wing] OR [Publication Title: far-left] OR [Publication Title: right-wing] OR [Publication Title: far-right] OR [Publication Title: neo-nazi*] OR [Publication Title: communis*] OR [Publication Title: nationalis*] OR [Publication Title: supremacist] OR [Publication Title: anarch*] OR [Publication Title: indoctrinat*]] AND [[Abstract: family] OR [Abstract: families] OR [Abstract: familial*] OR [Abstract: parent*] OR [Abstract: siblin*] OR [Abstract: brother*] OR [Abstract: sister*] OR [Abstract: father*] OR [Abstract: mother*] OR [Abstract: child*] OR [Abstract: son*] OR [Abstract: daughter*] OR [Abstract: cousin*] OR [Abstract: uncle*] OR [Abstract: aunt*] OR [Abstract: generation*] OR [Abstract: maternal] OR [Abstract: paternal] OR [Abstract: grandparent*]] AND [[Keywords: risk*] OR [Keywords: protect*] OR [Keywords: factor*] OR [Keywords: correlat*] OR [Keywords: relat*] OR [Keywords: predict*] OR [Keywords: caus*] OR [Keywords: determina*] OR [Keywords: consequenc*] OR [Keywords: interven*] OR [Keywords: evaluat*] OR [Keywords: program*] OR [Keywords: treat*] OR [Keywords: prevent*] OR [Keywords: experiment*] OR [Keywords: "cross-section*"] OR [Keywords: longitudinal*] OR [Keywords: regress*]]

Your search for [[Publication Title: radicali*] OR [Publication Title: terror*] OR [Publication Title: extremis*] OR [Publication Title: "lone wol*"] OR [Publication Title: lone-wol*] OR [Publication Title: "foreign fighter*"] OR [Publication Title: "single issue"] OR [Publication Title: jihad*] OR [Publication Title: islamis*] OR [Publication Title: salaf*] OR [Publication Title: left-wing] OR [Publication Title: far-left] OR [Publication Title: right-wing] OR [Publication Title: far-right] OR [Publication Title: neo-nazi*] OR [Publication Title: communis*] OR [Publication Title: nationalis*] OR [Publication Title: supremacist] OR [Publication Title: anarch*] OR [Publication Title: indoctrinat*]] AND [[Abstract: family] OR [Abstract: families] OR [Abstract: familial*] OR [Abstract: parent*] OR [Abstract: siblin*] OR [Abstract: brother*] OR [Abstract: sister*] OR [Abstract: father*] OR [Abstract: mother*] OR [Abstract: child*] OR [Abstract: son*] OR [Abstract: daughter*] OR [Abstract: cousin*] OR [Abstract: uncle*] OR [Abstract: aunt*] OR [Abstract: generation*] OR [Abstract: maternal] OR [Abstract: paternal] OR [Abstract: grandparent*]] AND [[Publication Title: risk*] OR [Publication Title: protect*] OR [Publication Title: factor*] OR [Publication Title: correlat*] OR [Publication Title: relat*] OR [Publication Title: predict*] OR [Publication Title: caus*] OR [Publication Title: determina*] OR [Publication Title: consequenc*] OR [Publication Title: interven*] OR [Publication Title: evaluat*] OR [Publication Title: program*] OR [Publication Title: treat*] OR [Publication Title: prevent*] OR [Publication Title: experiment*] OR [Publication Title: "cross-section*"] OR [Publication Title: longitudinal*] OR [Publication Title: regress*]]

Your search for [[Publication Title: radicali*] OR [Publication Title: terror*] OR [Publication Title: extremis*] OR [Publication Title: "lone wol*"] OR [Publication Title: lone-wol*] OR [Publication Title: "foreign fighter*"] OR [Publication Title: "single issue"] OR [Publication Title: jihad*] OR [Publication Title: islamis*] OR [Publication Title: salaf*] OR [Publication Title: left-wing] OR [Publication Title: far-left] OR [Publication Title: right-wing] OR [Publication Title: far-right] OR [Publication Title: neo-nazi*] OR [Publication Title: communis*] OR [Publication Title: nationalis*] OR [Publication Title: supremacist] OR [Publication Title: anarch*] OR [Publication Title: indoctrinat*]] AND [[Keywords: family] OR [Keywords: families] OR [Keywords: familial*] OR [Keywords: parent*] OR [Keywords: siblin*] OR [Keywords: brother*] OR [Keywords: sister*] OR [Keywords: father*] OR [Keywords: mother*] OR [Keywords: child*] OR [Keywords: son*] OR [Keywords: daughter*] OR [Keywords: cousin*] OR [Keywords: uncle*] OR [Keywords: aunt*] OR [Keywords: generation*] OR [Keywords: maternal] OR [Keywords: paternal] OR [Keywords: grandparent*]] AND [[Abstract: risk*] OR [Abstract: protect*] OR [Abstract: factor*] OR [Abstract: correlat*] OR [Abstract: relat*] OR [Abstract: predict*] OR [Abstract: caus*] OR [Abstract: determina*] OR [Abstract: consequenc*] OR [Abstract: interven*] OR [Abstract: evaluat*] OR [Abstract: program*] OR [Abstract: treat*] OR [Abstract: prevent*] OR [Abstract: experiment*] OR [Abstract: "cross-section*"] OR [Abstract: longitudinal*] OR [Abstract: regress*]]

Your search for [[Publication Title: radicali*] OR [Publication Title: terror*] OR [Publication Title: extremis*] OR [Publication Title: "lone wol*"] OR [Publication Title: lone-wol*] OR [Publication Title: "foreign fighter*"] OR [Publication Title: "single issue"] OR [Publication Title: jihad*] OR [Publication Title: islamis*] OR [Publication Title: salaf*] OR [Publication Title: left-wing] OR [Publication Title: far-left] OR [Publication Title: right-wing] OR [Publication Title: far-right] OR [Publication Title: neo-nazi*] OR [Publication Title: communis*] OR [Publication Title: nationalis*] OR [Publication Title: supremacist] OR [Publication Title: anarch*] OR [Publication Title: indoctrinat*]] AND [[Keywords: family] OR [Keywords: families] OR [Keywords: familial*] OR [Keywords: parent*] OR [Keywords: siblin*] OR [Keywords: brother*] OR [Keywords: sister*] OR [Keywords: father*] OR [Keywords: mother*] OR [Keywords: child*] OR [Keywords: son*] OR [Keywords: daughter*] OR [Keywords: cousin*] OR [Keywords: uncle*] OR [Keywords: aunt*] OR [Keywords: generation*] OR [Keywords: maternal] OR [Keywords: paternal] OR [Keywords: grandparent*]] AND [[Keywords: risk*] OR [Keywords: protect*] OR [Keywords: factor*] OR [Keywords: correlat*] OR [Keywords: relat*] OR [Keywords: predict*] OR [Keywords: caus*] OR [Keywords: determina*] OR [Keywords: consequenc*] OR [Keywords: interven*] OR [Keywords: evaluat*] OR [Keywords: program*] OR [Keywords: treat*] OR [Keywords: prevent*] OR [Keywords: experiment*] OR [Keywords: "cross-section*"] OR [Keywords: longitudinal*] OR [Keywords: regress*]]

Your search for [[Publication Title: radicali*] OR [Publication Title: terror*] OR [Publication Title: extremis*] OR [Publication Title: "lone wol*"] OR [Publication Title: lone-wol*] OR [Publication Title: "foreign fighter*"] OR [Publication Title: "single issue"] OR [Publication Title: jihad*] OR [Publication Title: islamis*] OR [Publication Title: salaf*] OR [Publication Title: left-wing] OR [Publication Title: far-left] OR [Publication Title: right-wing] OR [Publication Title: far-right] OR [Publication Title: neo-nazi*] OR [Publication Title: communis*] OR [Publication Title: nationalis*] OR [Publication Title: supremacist] OR [Publication Title: anarch*] OR [Publication Title: indoctrinat*]] AND [[Keywords: family] OR [Keywords: families] OR [Keywords: familial*] OR [Keywords: parent*] OR [Keywords: siblin*] OR [Keywords: brother*] OR [Keywords: sister*] OR [Keywords: father*] OR [Keywords: mother*] OR [Keywords: child*] OR [Keywords: son*] OR [Keywords: daughter*] OR [Keywords: cousin*] OR [Keywords: uncle*] OR [Keywords: aunt*] OR [Keywords: generation*] OR [Keywords: maternal] OR [Keywords: paternal] OR [Keywords: grandparent*]] AND [[Publication Title: risk*] OR [Publication Title: protect*] OR [Publication Title: factor*] OR [Publication Title: correlat*] OR [Publication Title: relat*] OR [Publication Title: predict*] OR [Publication Title: caus*] OR [Publication Title: determina*] OR [Publication Title: consequenc*] OR [Publication Title: interven*] OR [Publication Title: evaluat*] OR [Publication Title: program*] OR [Publication Title: treat*] OR [Publication Title: prevent*] OR [Publication Title: experiment*] OR [Publication Title: "cross-section*"] OR [Publication Title: longitudinal*] OR [Publication Title: regress*]]

Your search for [[Publication Title: radicali*] OR [Publication Title: terror*] OR [Publication Title: extremis*] OR [Publication Title: "lone wol*"] OR [Publication Title: lone-wol*] OR [Publication Title: "foreign fighter*"] OR [Publication Title: "single issue"] OR [Publication Title: jihad*] OR [Publication Title: islamis*] OR [Publication Title: salaf*] OR [Publication Title: left-wing] OR [Publication Title: far-left] OR [Publication Title: right-wing] OR [Publication Title: far-right] OR [Publication Title: neo-nazi*] OR [Publication Title: communis*] OR [Publication Title: nationalis*] OR [Publication Title: supremacist] OR [Publication Title: anarch*] OR [Publication Title: indoctrinat*]] AND [[Publication Title: family] OR [Publication Title: families] OR [Publication Title: familial*] OR [Publication Title: parent*] OR [Publication Title: siblin*] OR [Publication Title: brother*] OR [Publication Title: sister*] OR [Publication Title: father*] OR [Publication Title: mother*] OR [Publication Title: child*] OR [Publication Title: son*] OR [Publication Title: daughter*] OR [Publication Title: cousin*] OR [Publication Title: uncle*] OR [Publication Title: aunt*] OR [Publication Title: generation*] OR [Publication Title: maternal] OR [Publication Title: paternal] OR [Publication Title: grandparent*]] AND [[Abstract: risk*] OR [Abstract: protect*] OR [Abstract: factor*] OR [Abstract: correlat*] OR [Abstract: relat*] OR [Abstract: predict*] OR [Abstract: caus*] OR [Abstract: determina*] OR [Abstract: consequenc*] OR [Abstract: interven*] OR [Abstract: evaluat*] OR [Abstract: program*] OR [Abstract: treat*] OR [Abstract: prevent*] OR [Abstract: experiment*] OR [Abstract: "cross-section*"] OR [Abstract: longitudinal*] OR [Abstract: regress*]]

Your search for [[Publication Title: radicali*] OR [Publication Title: terror*] OR [Publication Title: extremis*] OR [Publication Title: "lone wol*"] OR [Publication Title: lone-wol*] OR [Publication Title: "foreign fighter*"] OR [Publication Title: "single issue"] OR [Publication Title: jihad*] OR [Publication Title: islamis*] OR [Publication Title: salaf*] OR [Publication Title: left-wing] OR [Publication Title: far-left] OR [Publication Title: right-wing] OR [Publication Title: far-right] OR [Publication Title: neo-nazi*] OR [Publication Title: communis*] OR [Publication Title: nationalis*] OR [Publication Title: supremacist] OR [Publication Title: anarch*] OR [Publication Title: indoctrinat*]] AND [[Publication Title: family] OR [Publication Title: families] OR [Publication Title: familial*] OR [Publication Title: parent*] OR [Publication Title: siblin*] OR [Publication Title: brother*] OR [Publication Title: sister*] OR [Publication Title: father*] OR [Publication Title: mother*] OR [Publication Title: child*] OR [Publication Title: son*] OR [Publication Title: daughter*] OR [Publication Title: cousin*] OR [Publication Title: uncle*] OR [Publication Title: aunt*] OR [Publication Title: generation*] OR [Publication Title: maternal] OR [Publication Title: paternal] OR [Publication Title: grandparent*]] AND [[Keywords: risk*] OR [Keywords: protect*] OR [Keywords: factor*] OR [Keywords: correlat*] OR [Keywords: relat*] OR [Keywords: predict*] OR [Keywords: caus*] OR [Keywords: determina*] OR [Keywords: consequenc*] OR [Keywords: interven*] OR [Keywords: evaluat*] OR [Keywords: program*] OR [Keywords: treat*] OR [Keywords: prevent*] OR [Keywords: experiment*] OR [Keywords: "cross-section*"] OR [Keywords: longitudinal*] OR [Keywords: regress*]]

**Web of Science**

**(1900-current)**

June 25, 2021

|  | TS  =  (radicali* OR terror* OR extremis* OR "lone wol*" OR lone-wol* OR "foreign fighter*" OR "single issue" OR Jihad* OR Islamis* OR Salaf* OR left-wing OR far-left OR right-wing OR far-right OR neo-nazi* OR communis* OR nationalis* OR supremacist OR anarch* OR indoctrinat*)  AND  TS  =  (family OR families OR familal* OR parent* OR siblin* OR brother* OR sister* OR father* OR mother* OR child* OR son* OR daughter* OR cousin* OR uncle* OR aunt* OR generation* OR maternal OR paternal OR grandparent*)  AND  TS  =  (risk* OR protect* OR factor OR correlat* OR relat* OR predict* OR caus* OR determina* OR consequenc* OR intervent* OR evaluat* OR program* OR treat*OR prevent* OR experiment* OR "cross-section*" OR longitudinal* OR regress*)  Databases= WOS, CCC, DIIDW, KJD, MEDLINE, RSCI, SCIELO Time=All years  Language=Auto |
| --- | --- |

**Wiley (1799- current)**

July 14-29, 2021

"radicali* OR terror* OR extremis* OR"lone wolf" OR “lone-wolf” OR "foreign fighter*" OR "single issue" OR jihad* OR islamis* OR salaf* OR “left-wing” OR “far-left” OR “right-wing” OR “far-right” OR “neo-nazi” OR communis* OR nationalis* OR supremacist OR anarch* OR indoctrinat* AND family OR families OR familial* OR parent* OR siblin* OR brother* OR sister* OR father* OR mother* OR child* OR son* OR daughter* OR cousin* OR uncle* OR aunt* OR generation* OR maternal OR paternal OR grandparent* AND risk* OR protect* OR factor* OR correlat* OR relat* OR predict* OR caus* OR determina* OR consequenc* OR interven* OR evaluat* OR program* OR treat* OR prevent* OR experiment* OR “cross-section*” OR longitudinal* OR regress*" in Abstract

"radicali* OR terror* OR extremis* OR"lone wolf" OR “lone-wolf” OR "foreign fighter*" OR "single issue" OR jihad* OR islamis* OR salaf* OR “left-wing” OR “far-left” OR “right-wing” OR “far-right” OR “neo-nazi” OR communis* OR nationalis* OR supremacist OR anarch* OR indoctrinat*" in Abstract and "family OR families OR familial* OR parent* OR siblin* OR brother* OR sister* OR father* OR mother* OR child* OR son* OR daughter* OR cousin* OR uncle* OR aunt* OR generation* OR maternal OR paternal OR grandparent*" in Abstract and "risk* OR protect* OR factor* OR correlat* OR relat* OR predict* OR caus* OR determina* OR consequenc* OR interven* OR evaluat* OR program* OR treat* OR prevent* OR experiment* OR “cross-section*” OR longitudinal* OR regress*" in Keywords

"radicali* OR terror* OR extremis* OR"lone wolf" OR “lone-wolf” OR "foreign fighter*" OR "single issue" OR jihad* OR islamis* OR salaf* OR “left-wing” OR “far-left” OR “right-wing” OR “far-right” OR “neo-nazi” OR communis* OR nationalis* OR supremacist OR anarch* OR indoctrinat*" in Abstract and "family OR families OR familial* OR parent* OR siblin* OR brother* OR sister* OR father* OR mother* OR child* OR son* OR daughter* OR cousin* OR uncle* OR aunt* OR generation* OR maternal OR paternal OR grandparent*" in Abstract and "risk* OR protect* OR factor* OR correlat* OR relat* OR predict* OR caus* OR determina* OR consequenc* OR interven* OR evaluat* OR program* OR treat* OR prevent* OR experiment* OR “cross-section*” OR longitudinal* OR regress*" in Title

"radicali* OR terror* OR extremis* OR"lone wolf" OR “lone-wolf” OR "foreign fighter*" OR "single issue" OR jihad* OR islamis* OR salaf* OR “left-wing” OR “far-left” OR “right-wing” OR “far-right” OR “neo-nazi” OR communis* OR nationalis* OR supremacist OR anarch* OR indoctrinat*" in Abstract and "family OR families OR familial* OR parent* OR siblin* OR brother* OR sister* OR father* OR mother* OR child* OR son* OR daughter* OR cousin* OR uncle* OR aunt* OR generation* OR maternal OR paternal OR grandparent*" in Keywords and "risk* OR protect* OR factor* OR correlat* OR relat* OR predict* OR caus* OR determina* OR consequenc* OR interven* OR evaluat* OR program* OR treat* OR prevent* OR experiment* OR “cross-section*” OR longitudinal* OR regress*" in Abstract

"radicali* OR terror* OR extremis* OR"lone wolf" OR “lone-wolf” OR "foreign fighter*" OR "single issue" OR jihad* OR islamis* OR salaf* OR “left-wing” OR “far-left” OR “right-wing” OR “far-right” OR “neo-nazi” OR communis* OR nationalis* OR supremacist OR anarch* OR indoctrinat*" in Abstract and "family OR families OR familial* OR parent* OR siblin* OR brother* OR sister* OR father* OR mother* OR child* OR son* OR daughter* OR cousin* OR uncle* OR aunt* OR generation* OR maternal OR paternal OR grandparent*" in Keywords and "risk* OR protect* OR factor* OR correlat* OR relat* OR predict* OR caus* OR determina* OR consequenc* OR interven* OR evaluat* OR program* OR treat* OR prevent* OR experiment* OR “cross-section*” OR longitudinal* OR regress*" in Keywords

"radicali* OR terror* OR extremis* OR"lone wolf" OR “lone-wolf” OR "foreign fighter*" OR "single issue" OR jihad* OR islamis* OR salaf* OR “left-wing” OR “far-left” OR “right-wing” OR “far-right” OR “neo-nazi” OR communis* OR nationalis* OR supremacist OR anarch* OR indoctrinat*" in Abstract and "family OR families OR familial* OR parent* OR siblin* OR brother* OR sister* OR father* OR mother* OR child* OR son* OR daughter* OR cousin* OR uncle* OR aunt* OR generation* OR maternal OR paternal OR grandparent*" in Keywords and "risk* OR protect* OR factor* OR correlat* OR relat* OR predict* OR caus* OR determina* OR consequenc* OR interven* OR evaluat* OR program* OR treat* OR prevent* OR experiment* OR “cross-section*” OR longitudinal* OR regress*" in Title

"radicali* OR terror* OR extremis* OR"lone wolf" OR “lone-wolf” OR "foreign fighter*" OR "single issue" OR jihad* OR islamis* OR salaf* OR “left-wing” OR “far-left” OR “right-wing” OR “far-right” OR “neo-nazi” OR communis* OR nationalis* OR supremacist OR anarch* OR indoctrinat*" in Abstract and "family OR families OR familial* OR parent* OR siblin* OR brother* OR sister* OR father* OR mother* OR child* OR son* OR daughter* OR cousin* OR uncle* OR aunt* OR generation* OR maternal OR paternal OR grandparent*" in Title and "risk* OR protect* OR factor* OR correlat* OR relat* OR predict* OR caus* OR determina* OR consequenc* OR interven* OR evaluat* OR program* OR treat* OR prevent* OR experiment* OR “cross-section*” OR longitudinal* OR regress*" in Abstract

"radicali* OR terror* OR extremis* OR"lone wolf" OR “lone-wolf” OR "foreign fighter*" OR "single issue" OR jihad* OR islamis* OR salaf* OR “left-wing” OR “far-left” OR “right-wing” OR “far-right” OR “neo-nazi” OR communis* OR nationalis* OR supremacist OR anarch* OR indoctrinat*" in Abstract and "family OR families OR familial* OR parent* OR siblin* OR brother* OR sister* OR father* OR mother* OR child* OR son* OR daughter* OR cousin* OR uncle* OR aunt* OR generation* OR maternal OR paternal OR grandparent*" in Title and "risk* OR protect* OR factor* OR correlat* OR relat* OR predict* OR caus* OR determina* OR consequenc* OR interven* OR evaluat* OR program* OR treat* OR prevent* OR experiment* OR “cross-section*” OR longitudinal* OR regress*" in Keywords

"radicali* OR terror* OR extremis* OR"lone wolf" OR “lone-wolf” OR "foreign fighter*" OR "single issue" OR jihad* OR islamis* OR salaf* OR “left-wing” OR “far-left” OR “right-wing” OR “far-right” OR “neo-nazi” OR communis* OR nationalis* OR supremacist OR anarch* OR indoctrinat*" in Abstract and "family OR families OR familial* OR parent* OR siblin* OR brother* OR sister* OR father* OR mother* OR child* OR son* OR daughter* OR cousin* OR uncle* OR aunt* OR generation* OR maternal OR paternal OR grandparent*" in Title and "risk* OR protect* OR factor* OR correlat* OR relat* OR predict* OR caus* OR determina* OR consequenc* OR interven* OR evaluat* OR program* OR treat* OR prevent* OR experiment* OR “cross-section*” OR longitudinal* OR regress*" in Title

"radicali* OR terror* OR extremis* OR"lone wolf" OR “lone-wolf” OR "foreign fighter*" OR "single issue" OR jihad* OR islamis* OR salaf* OR “left-wing” OR “far-left” OR “right-wing” OR “far-right” OR “neo-nazi” OR communis* OR nationalis* OR supremacist OR anarch* OR indoctrinat*" in Keywords and "family OR families OR familial* OR parent* OR siblin* OR brother* OR sister* OR father* OR mother* OR child* OR son* OR daughter* OR cousin* OR uncle* OR aunt* OR generation* OR maternal OR paternal OR grandparent*" in Abstract and "risk* OR protect* OR factor* OR correlat* OR relat* OR predict* OR caus* OR determina* OR consequenc* OR interven* OR evaluat* OR program* OR treat* OR prevent* OR experiment* OR “cross-section*” OR longitudinal* OR regress*" in Abstract

"radicali* OR terror* OR extremis* OR"lone wolf" OR “lone-wolf” OR "foreign fighter*" OR "single issue" OR jihad* OR islamis* OR salaf* OR “left-wing” OR “far-left” OR “right-wing” OR “far-right” OR “neo-nazi” OR communis* OR nationalis* OR supremacist OR anarch* OR indoctrinat*" in Keywords and "family OR families OR familial* OR parent* OR siblin* OR brother* OR sister* OR father* OR mother* OR child* OR son* OR daughter* OR cousin* OR uncle* OR aunt* OR generation* OR maternal OR paternal OR grandparent*" in Abstract and "risk* OR protect* OR factor* OR correlat* OR relat* OR predict* OR caus* OR determina* OR consequenc* OR interven* OR evaluat* OR program* OR treat* OR prevent* OR experiment* OR “cross-section*” OR longitudinal* OR regress*" in Keywords

"radicali* OR terror* OR extremis* OR"lone wolf" OR “lone-wolf” OR "foreign fighter*" OR "single issue" OR jihad* OR islamis* OR salaf* OR “left-wing” OR “far-left” OR “right-wing” OR “far-right” OR “neo-nazi” OR communis* OR nationalis* OR supremacist OR anarch* OR indoctrinat*" in Keywords and "family OR families OR familial* OR parent* OR siblin* OR brother* OR sister* OR father* OR mother* OR child* OR son* OR daughter* OR cousin* OR uncle* OR aunt* OR generation* OR maternal OR paternal OR grandparent*" in Abstract and "risk* OR protect* OR factor* OR correlat* OR relat* OR predict* OR caus* OR determina* OR consequenc* OR interven* OR evaluat* OR program* OR treat* OR prevent* OR experiment* OR “cross-section*” OR longitudinal* OR regress*" in Title

"radicali* OR terror* OR extremis* OR"lone wolf" OR “lone-wolf” OR "foreign fighter*" OR "single issue" OR jihad* OR islamis* OR salaf* OR “left-wing” OR “far-left” OR “right-wing” OR “far-right” OR “neo-nazi” OR communis* OR nationalis* OR supremacist OR anarch* OR indoctrinat*" in Keywords and "family OR families OR familial* OR parent* OR siblin* OR brother* OR sister* OR father* OR mother* OR child* OR son* OR daughter* OR cousin* OR uncle* OR aunt* OR generation* OR maternal OR paternal OR grandparent*" in Keywords and "risk* OR protect* OR factor* OR correlat* OR relat* OR predict* OR caus* OR determina* OR consequenc* OR interven* OR evaluat* OR program* OR treat* OR prevent* OR experiment* OR “cross-section*” OR longitudinal* OR regress*" in Abstract

"radicali* OR terror* OR extremis* OR"lone wolf" OR “lone-wolf” OR "foreign fighter*" OR "single issue" OR jihad* OR islamis* OR salaf* OR “left-wing” OR “far-left” OR “right-wing” OR “far-right” OR “neo-nazi” OR communis* OR nationalis* OR supremacist OR anarch* OR indoctrinat*" in Keywords and "family OR families OR familial* OR parent* OR siblin* OR brother* OR sister* OR father* OR mother* OR child* OR son* OR daughter* OR cousin* OR uncle* OR aunt* OR generation* OR maternal OR paternal OR grandparent*" in Keywords and "risk* OR protect* OR factor* OR correlat* OR relat* OR predict* OR caus* OR determina* OR consequenc* OR interven* OR evaluat* OR program* OR treat* OR prevent* OR experiment* OR “cross-section*” OR longitudinal* OR regress*" in Keywords

"radicali* OR terror* OR extremis* OR"lone wolf" OR “lone-wolf” OR "foreign fighter*" OR "single issue" OR jihad* OR islamis* OR salaf* OR “left-wing” OR “far-left” OR “right-wing” OR “far-right” OR “neo-nazi” OR communis* OR nationalis* OR supremacist OR anarch* OR indoctrinat*" in Keywords and "family OR families OR familial* OR parent* OR siblin* OR brother* OR sister* OR father* OR mother* OR child* OR son* OR daughter* OR cousin* OR uncle* OR aunt* OR generation* OR maternal OR paternal OR grandparent*" in Keywords and "risk* OR protect* OR factor* OR correlat* OR relat* OR predict* OR caus* OR determina* OR consequenc* OR interven* OR evaluat* OR program* OR treat* OR prevent* OR experiment* OR “cross-section*” OR longitudinal* OR regress*" in Title

"radicali* OR terror* OR extremis* OR"lone wolf" OR “lone-wolf” OR "foreign fighter*" OR "single issue" OR jihad* OR islamis* OR salaf* OR “left-wing” OR “far-left” OR “right-wing” OR “far-right” OR “neo-nazi” OR communis* OR nationalis* OR supremacist OR anarch* OR indoctrinat*" in Keywords and "family OR families OR familial* OR parent* OR siblin* OR brother* OR sister* OR father* OR mother* OR child* OR son* OR daughter* OR cousin* OR uncle* OR aunt* OR generation* OR maternal OR paternal OR grandparent*" in Title and "risk* OR protect* OR factor* OR correlat* OR relat* OR predict* OR caus* OR determina* OR consequenc* OR interven* OR evaluat* OR program* OR treat* OR prevent* OR experiment* OR “cross-section*” OR longitudinal* OR regress*" in Abstract

"radicali* OR terror* OR extremis* OR"lone wolf" OR “lone-wolf” OR "foreign fighter*" OR "single issue" OR jihad* OR islamis* OR salaf* OR “left-wing” OR “far-left” OR “right-wing” OR “far-right” OR “neo-nazi” OR communis* OR nationalis* OR supremacist OR anarch* OR indoctrinat*" in Keywords and "family OR families OR familial* OR parent* OR siblin* OR brother* OR sister* OR father* OR mother* OR child* OR son* OR daughter* OR cousin* OR uncle* OR aunt* OR generation* OR maternal OR paternal OR grandparent*" in Title and "risk* OR protect* OR factor* OR correlat* OR relat* OR predict* OR caus* OR determina* OR consequenc* OR interven* OR evaluat* OR program* OR treat* OR prevent* OR experiment* OR “cross-section*” OR longitudinal* OR regress*" in Keywords

"radicali* OR terror* OR extremis* OR"lone wolf" OR “lone-wolf” OR "foreign fighter*" OR "single issue" OR jihad* OR islamis* OR salaf* OR “left-wing” OR “far-left” OR “right-wing” OR “far-right” OR “neo-nazi” OR communis* OR nationalis* OR supremacist OR anarch* OR indoctrinat*" in Keywords and "family OR families OR familial* OR parent* OR siblin* OR brother* OR sister* OR father* OR mother* OR child* OR son* OR daughter* OR cousin* OR uncle* OR aunt* OR generation* OR maternal OR paternal OR grandparent*" in Title and "risk* OR protect* OR factor* OR correlat* OR relat* OR predict* OR caus* OR determina* OR consequenc* OR interven* OR evaluat* OR program* OR treat* OR prevent* OR experiment* OR “cross-section*” OR longitudinal* OR regress*" in Title

"radicali* OR terror* OR extremis* OR"lone wolf" OR “lone-wolf” OR "foreign fighter*" OR "single issue" OR jihad* OR islamis* OR salaf* OR “left-wing” OR “far-left” OR “right-wing” OR “far-right” OR “neo-nazi” OR communis* OR nationalis* OR supremacist OR anarch* OR indoctrinat*" in Title and "family OR families OR familial* OR parent* OR siblin* OR brother* OR sister* OR father* OR mother* OR child* OR son* OR daughter* OR cousin* OR uncle* OR aunt* OR generation* OR maternal OR paternal OR grandparent*" in Abstract and "risk* OR protect* OR factor* OR correlat* OR relat* OR predict* OR caus* OR determina* OR consequenc* OR interven* OR evaluat* OR program* OR treat* OR prevent* OR experiment* OR “cross-section*” OR longitudinal* OR regress*" in Abstract

"radicali* OR terror* OR extremis* OR"lone wolf" OR “lone-wolf” OR "foreign fighter*" OR "single issue" OR jihad* OR islamis* OR salaf* OR “left-wing” OR “far-left” OR “right-wing” OR “far-right” OR “neo-nazi” OR communis* OR nationalis* OR supremacist OR anarch* OR indoctrinat*" in Title and "family OR families OR familial* OR parent* OR siblin* OR brother* OR sister* OR father* OR mother* OR child* OR son* OR daughter* OR cousin* OR uncle* OR aunt* OR generation* OR maternal OR paternal OR grandparent*" in Abstract and "risk* OR protect* OR factor* OR correlat* OR relat* OR predict* OR caus* OR determina* OR consequenc* OR interven* OR evaluat* OR program* OR treat* OR prevent* OR experiment* OR “cross-section*” OR longitudinal* OR regress*" in Keywords

"radicali* OR terror* OR extremis* OR"lone wolf" OR “lone-wolf” OR "foreign fighter*" OR "single issue" OR jihad* OR islamis* OR salaf* OR “left-wing” OR “far-left” OR “right-wing” OR “far-right” OR “neo-nazi” OR communis* OR nationalis* OR supremacist OR anarch* OR indoctrinat*" in Title and "family OR families OR familial* OR parent* OR siblin* OR brother* OR sister* OR father* OR mother* OR child* OR son* OR daughter* OR cousin* OR uncle* OR aunt* OR generation* OR maternal OR paternal OR grandparent*" in Abstract and "risk* OR protect* OR factor* OR correlat* OR relat* OR predict* OR caus* OR determina* OR consequenc* OR interven* OR evaluat* OR program* OR treat* OR prevent* OR experiment* OR “cross-section*” OR longitudinal* OR regress*" in Title

"radicali* OR terror* OR extremis* OR"lone wolf" OR “lone-wolf” OR "foreign fighter*" OR "single issue" OR jihad* OR islamis* OR salaf* OR “left-wing” OR “far-left” OR “right-wing” OR “far-right” OR “neo-nazi” OR communis* OR nationalis* OR supremacist OR anarch* OR indoctrinat*" in Title and "family OR families OR familial* OR parent* OR siblin* OR brother* OR sister* OR father* OR mother* OR child* OR son* OR daughter* OR cousin* OR uncle* OR aunt* OR generation* OR maternal OR paternal OR grandparent*" in Keywords and "risk* OR protect* OR factor* OR correlat* OR relat* OR predict* OR caus* OR determina* OR consequenc* OR interven* OR evaluat* OR program* OR treat* OR prevent* OR experiment* OR “cross-section*” OR longitudinal* OR regress*" in Abstract

"radicali* OR terror* OR extremis* OR"lone wolf" OR “lone-wolf” OR "foreign fighter*" OR "single issue" OR jihad* OR islamis* OR salaf* OR “left-wing” OR “far-left” OR “right-wing” OR “far-right” OR “neo-nazi” OR communis* OR nationalis* OR supremacist OR anarch* OR indoctrinat*" in Title and "family OR families OR familial* OR parent* OR siblin* OR brother* OR sister* OR father* OR mother* OR child* OR son* OR daughter* OR cousin* OR uncle* OR aunt* OR generation* OR maternal OR paternal OR grandparent*" in Keywords and "risk* OR protect* OR factor* OR correlat* OR relat* OR predict* OR caus* OR determina* OR consequenc* OR interven* OR evaluat* OR program* OR treat* OR prevent* OR experiment* OR “cross-section*” OR longitudinal* OR regress*" in Keywords

"radicali* OR terror* OR extremis* OR"lone wolf" OR “lone-wolf” OR "foreign fighter*" OR "single issue" OR jihad* OR islamis* OR salaf* OR “left-wing” OR “far-left” OR “right-wing” OR “far-right” OR “neo-nazi” OR communis* OR nationalis* OR supremacist OR anarch* OR indoctrinat*" in Title and "family OR families OR familial* OR parent* OR siblin* OR brother* OR sister* OR father* OR mother* OR child* OR son* OR daughter* OR cousin* OR uncle* OR aunt* OR generation* OR maternal OR paternal OR grandparent*" in Keywords and "risk* OR protect* OR factor* OR correlat* OR relat* OR predict* OR caus* OR determina* OR consequenc* OR interven* OR evaluat* OR program* OR treat* OR prevent* OR experiment* OR “cross-section*” OR longitudinal* OR regress*" in Title

"radicali* OR terror* OR extremis* OR"lone wolf" OR “lone-wolf” OR "foreign fighter*" OR "single issue" OR jihad* OR islamis* OR salaf* OR “left-wing” OR “far-left” OR “right-wing” OR “far-right” OR “neo-nazi” OR communis* OR nationalis* OR supremacist OR anarch* OR indoctrinat*" in Title and "family OR families OR familial* OR parent* OR siblin* OR brother* OR sister* OR father* OR mother* OR child* OR son* OR daughter* OR cousin* OR uncle* OR aunt* OR generation* OR maternal OR paternal OR grandparent*" in Title and "risk* OR protect* OR factor* OR correlat* OR relat* OR predict* OR caus* OR determina* OR consequenc* OR interven* OR evaluat* OR program* OR treat* OR prevent* OR experiment* OR “cross-section*” OR longitudinal* OR regress*" in Abstract

"radicali* OR terror* OR extremis* OR"lone wolf" OR “lone-wolf” OR "foreign fighter*" OR "single issue" OR jihad* OR islamis* OR salaf* OR “left-wing” OR “far-left” OR “right-wing” OR “far-right” OR “neo-nazi” OR communis* OR nationalis* OR supremacist OR anarch* OR indoctrinat*" in Title and "family OR families OR familial* OR parent* OR siblin* OR brother* OR sister* OR father* OR mother* OR child* OR son* OR daughter* OR cousin* OR uncle* OR aunt* OR generation* OR maternal OR paternal OR grandparent*" in Title and "risk* OR protect* OR factor* OR correlat* OR relat* OR predict* OR caus* OR determina* OR consequenc* OR interven* OR evaluat* OR program* OR treat* OR prevent* OR experiment* OR “cross-section*” OR longitudinal* OR regress*" in Keywords

"radicali* OR terror* OR extremis* OR"lone wolf" OR “lone-wolf” OR "foreign fighter*" OR "single issue" OR jihad* OR islamis* OR salaf* OR “left-wing” OR “far-left” OR “right-wing” OR “far-right” OR “neo-nazi” OR communis* OR nationalis* OR supremacist OR anarch* OR indoctrinat*" in Title and "family OR families OR familial* OR parent* OR siblin* OR brother* OR sister* OR father* OR mother* OR child* OR son* OR daughter* OR cousin* OR uncle* OR aunt* OR generation* OR maternal OR paternal OR grandparent*" in Title and "risk* OR protect* OR factor* OR correlat* OR relat* OR predict* OR caus* OR determina* OR consequenc* OR interven* OR evaluat* OR program* OR treat* OR prevent* OR experiment* OR “cross-section*” OR longitudinal* OR regress*" in Title

**EBSCO (Criminal Justice Abstracts)**

**(1910-current)**

July 14, 2021

(radicali* OR terror* OR extremis* OR “lone wol*” OR lone-wol* OR “foreign fighter*” OR “single issue” OR Jihad* OR Islamis* OR Salaf* OR left-wing OR far-left OR right-wing OR far-right OR neo-nazi* OR communis* OR nationalis* OR supremacist OR anarch* OR indoctrinat*) AND (family OR families OR familial OR parent* OR siblin* OR brother* OR sister* OR father* OR mother* OR child* OR son* OR daughter* OR cousin* OR uncle* OR aunt* OR generation* OR maternal OR paternal OR grandparent*) AND (risk* OR protect* OR factor* OR correlat* OR relat* OR predict* OR caus* OR determina* OR consequenc* OR interven* OR evaluat* OR program* OR treat* OR prevent* OR experiment* OR “cross-section*” OR longitudinal* OR regress*)
