# Supplementary material for: Is radicalization a family issue? A systematic review of family‐related risk and protective factors, consequences, and interventions against radicalization
Source: Campbell Syst Rev. 2022 Jul 20;18(3):e1266. doi: 10.1002/cl2.1266 (PMC9300959; doi:10.1002/cl2.1266)
Supplement: Supplementary file 2 — Supporting information. [file CL2-18-e1266-s002.docx]

Appendix 2: Funnel plots

Figure 15. Funnel for the relation between critical family events and radicalization

Figure 16. Funnel for the relation between family commitment and radicalization

Figure 17. Funnel for the relation between family conflict and radicalization

Figure 18. Funnel for the relation between family size and radicalization

Figure 19. Funnel for the relation between family socioeconomic factors and radicalization

Figure 20. Funnel for the relation between family violence and radicalization

Figure 21. Funnel for the relation between being married and radicalization

Figure 22. Funnel for the relation between parental control and radicalization
